# Multiomic analysis of malignant pleural mesothelioma identifies molecular axes and specialized tumor profiles driving intertumor heterogeneity

**DOI:** 10.1038/s41588-023-01321-1

**Published:** 2023-03-16

**Authors:** Lise Mangiante, Nicolas Alcala, Alexandra Sexton-Oates, Alex Di Genova, Abel Gonzalez-Perez, Azhar Khandekar, Erik N. Bergstrom, Jaehee Kim, Xiran Liu, Ricardo Blazquez-Encinas, Colin Giacobi, Nolwenn Le Stang, Sandrine Boyault, Cyrille Cuenin, Severine Tabone-Eglinger, Francesca Damiola, Catherine Voegele, Maude Ardin, Marie-Cecile Michallet, Lorraine Soudade, Tiffany M. Delhomme, Arnaud Poret, Marie Brevet, Marie-Christine Copin, Sophie Giusiano-Courcambeck, Diane Damotte, Cecile Girard, Veronique Hofman, Paul Hofman, Jérôme Mouroux, Charlotte Cohen, Stephanie Lacomme, Julien Mazieres, Vincent Thomas de Montpreville, Corinne Perrin, Gaetane Planchard, Nathalie Rousseau, Isabelle Rouquette, Christine Sagan, Arnaud Scherpereel, Francoise Thivolet, Jean-Michel Vignaud, Didier Jean, Anabelle Gilg Soit Ilg, Robert Olaso, Vincent Meyer, Anne Boland-Auge, Jean-Francois Deleuze, Janine Altmuller, Peter Nuernberg, Alejandro Ibáñez-Costa, Justo P. Castaño, Sylvie Lantuejoul, Akram Ghantous, Charles Maussion, Pierre Courtiol, Hector Hernandez-Vargas, Christophe Caux, Nicolas Girard, Nuria Lopez-Bigas, Ludmil B. Alexandrov, Françoise Galateau-Salle, Matthieu Foll, Lynnette Fernandez-Cuesta

**Affiliations:** 1 Rare Cancers Genomics Team, Genomic Epidemiology Branch, International Agency for Research on Cancer/World Health Organization, Lyon, France; 2 Department of Medicine, Stanford University, Stanford, CA USA; 3 Instituto de Ciencias de la Ingeniería, Universidad de O’Higgins, Rancagua, Chile; 4 Centro de Modelamiento Matemático UMI-CNRS 2807, Universidad de Chile, Santiago, Chile; 5 Institute for Research in Biomedicine (IRB Barcelona), The Barcelona Institute of Science and Technology, Barcelona, Spain; 6 Centro de Investigación Biomédica en Red en Cáncer, Instituto de Salud Carlos III, Madrid, Spain; 7 Department of Cellular and Molecular Medicine, Department of Bioengineering and Moores Cancer Center, University of California, San Diego, La Jolla, CA USA; 8 Institute for Computational and Mathematical Engineering, Stanford University, Stanford, CA USA; 9 Department of Computational Biology, Cornell University, Ithaca, NY USA; 10 Maimonides Biomedical Research Institute of Cordoba, Córdoba, Spain; 11 Department of Cell Biology, Physiology and Immunology, University of Cordoba, Córdoba, Spain; 12 Reina Sofia University Hospital, Córdoba, Spain; 13 CIBER Fisiopatología de la Obesidad y Nutrición, Córdoba, Spain; 14 UMR INSERM 1052, CNRS 5286, Cancer Research Center of Lyon, MESOPATH-MESOBANK, Department of Biopathology, Cancer Centre Léon Bérard, Lyon, France; 15 Cancer Genomic Platform, Translational Research and Innovation Department, Centre Léon Bérard, Lyon, France; 16 EpiGenomics and Mechanisms Branch, International Agency for Research on Cancer/World Health Organization, Lyon, France; 17 Tumor Escape, Resistance and Immunity Department, Centre de Recherche en Cancérologie de Lyon, Centre Léon Bérard, Université de Lyon, Université Claude Bernard Lyon 1, INSERM 1052, CNRS 5286, Lyon, France; 18 Cypath and Cypath-rb, Villeurbanne, France; 19 University of Lille, Centre Hospitalier Universitaire Lille, Institut de Pathologie, Tumorothèque du Centre de Référence Régional en Cancérologie, Lille, France; 20 Department of Pathology, Centre Hospitalier Universitaire Nord, Marseille, France; 21 Centre de Recherche des Cordeliers, Inflammation, Complement and Cancer Team, Sorbonne Université, INSERM, Université de Paris, Paris, France; 22 Department of Pathology, Hôpitaux Universitaire Paris Centre, Tumorothèque/CRB Cancer, Cochin Hospital, Assistance Publique–Hôpitaux de Paris, Paris, France; 23 Tumorothèque Centre Hospitalier Universitaire de Nantes, Nantes, France; 24 Université Côte d’Azur, Laboratory of Clinical and Experimental Pathology, Nice Center Hospital, FHU OncoAge, Biobank BB-0033-00025 and IRCAN Inserm U1081/CNRS 7284, Nice, France; 25 Université Côte d’Azur, Department of Thoracic Surgery, Nice Center Hospital, FHU OncoAge and IRCAN Inserm U1081/CNRS 7284, Nice, France; 26 Department of Thoracic Surgery, FHU OncoAge, Nice Pasteur Hospital, Université Côte d’Azur, Nice, France; 27 Nancy Regional University Hospital, Centre Hospitalier Régional Universitaire, CRB BB-0033-00035, INSERM U1256, Nancy, France; 28 Toulouse University Hospital, Université Paul Sabatier, Toulouse, France; 29 Department of Pathology, Marie Lannelongue Hospital, Le Plessis Robinson, France; 30 Hospices Civils de Lyon, Institut de Pathologie, Centre de Ressources Biologiques des HCL, Tissu-Tumorothèque Est, Lyon, France; 31 Centre Hospitalier Universitaire de Caen, MESOPATH Regional Center, Caen, France; 32 Centre de Pathologie des Côteaux, Centre de Ressources Biologiques (CRB Cancer), IUCT Oncopole, Toulouse, France; 33 University of Lille, Centre Hospitalier Universitaire Lille, INSERM, OncoThAI, NETMESO Network, Lille, France; 34 Department of Biopathology, Centre Hospitalier Régional Universitaire de Nancy, Vandoeuvre-les-Nancy, France; 35 BRC, BB-0033-00035, Centre Hospitalier Régional Universitaire de Nancy, Vandoeuvre-les-Nancy, France; 36 Centre de Recherche des Cordeliers, INSERM, Sorbonne Université, Université de Paris, Functional Genomics of Solid Tumors, Paris, France; 37 Direction Santé Environnement Travail, Santé Publique France, Paris, France; 38 Université Paris-Saclay, CEA, Centre National de Recherche en Génomique Humaine, Evry, France; 39 Cologne Centre for Genomics, Cologne, Germany; 40 Grenoble Alpes University, Saint-Martin-d’Hères, France; 41 Owkin, New York, NY USA; 42 UMR INSERM 1052, CNRS 5286, UCBL1, Centre Léon Bérard, Lyon, France; 43 Centre de Recherche en Cancérologie de Lyon, Lyon, France; 44 Institut Curie, Institut du Thorax Curie Montsouris, Paris, France; 45 Université de Versailles Saint-Quentin-en-Yvelines, Université Paris-Saclay, Versailles, France; 46 Institució Catalana de Recerca i Estudis Avançats, Barcelona, Spain

**Keywords:** Mesothelioma, Genomics

## Abstract

Malignant pleural mesothelioma (MPM) is an aggressive cancer with rising incidence and challenging clinical management. Through a large series of whole-genome sequencing data, integrated with transcriptomic and epigenomic data using multiomics factor analysis, we demonstrate that the current World Health Organization classification only accounts for up to 10% of interpatient molecular differences. Instead, the MESOMICS project paves the way for a morphomolecular classification of MPM based on four dimensions: ploidy, tumor cell morphology, adaptive immune response and CpG island methylator profile. We show that these four dimensions are complementary, capture major interpatient molecular differences and are delimited by extreme phenotypes that—in the case of the interdependent tumor cell morphology and adapted immune response—reflect tumor specialization. These findings unearth the interplay between MPM functional biology and its genomic history, and provide insights into the variations observed in the clinical behavior of patients with MPM.

## Abstract (web summary)

Genomic, transcriptomic and epigenetic analyses of malignant pleural mesothelioma identify molecular axes and specialized tumor profiles underlying intertumoral heterogeneity.

## Main

Malignant pleural mesothelioma (MPM) is a rare and aggressive disease associated with asbestos exposure^1^. The World Health Organization (WHO) histological classification distinguishes three major types with prognostic value: epithelioid (MME), biphasic (MMB) and sarcomatoid (MMS)^2^. In the past decade, genomic studies uncovered molecular profiles (clusters) related to MPM’s histopathological classification, each enriched for somatic alterations in known cancer genes (for example, *BAP1* in MME and *TP53* in MMS)^3–5^. We and others undertook unsupervised analyses of these data, revealing a molecular continuum of types that explained the prognosis of the disease more accurately than any reported discrete cluster^6,7^. MPM interpatient heterogeneity at the biological and clinical level is therefore expected to be sufficiently explained by the histopathological classification, with phenotypes ranging from MME to MMS^8,9^.

Nevertheless, the full extent of MPM phenotypes and the mechanisms by which they evolved are poorly understood. Histopathological features (such as architectural subtypes) and molecular features (such as aneuploidy and immune infiltration) were shown to be independent of histopathological type^8,9^, suggesting that there are additional sources of heterogeneity that remain unexplained. In addition, although malignant transformation and cancer development can depend on a wide range of genomic aberrations^10–12^, genomic events have not been fully described in MPM as previous efforts have been restricted to profiling only exomes or a reduced representation of genomes^3–5,13^. As a result, biological functions performed by tumor cells, and the role of genomic events in shaping these functions, remain largely unknown, hindering any meaningful progress in the diagnosis, classification and treatment of the disease^8^.

We designed the MESOMICS study to uncover the main sources of molecular variation explaining MPM intertumoral heterogeneity, and to identify the underlying biological functions. Using multiomic analyses combining genomic, transcriptomic and epigenomic data on a novel cohort of 120 MPM tumors (Supplementary Tables 1–3), we show that the current histopathological classification only explains a fraction of the molecular heterogeneity of the disease, while ploidy, adaptive immune response and CpG island methylation are as important. Taking advantage of a large cohort of whole-genome sequencing (WGS) data, we map the molecular landscape of 120 MPMs and elucidate the link between genotype and phenotype.

## Results

### Multiomic analyses uncover four axes of molecular variation

**Fig. 1:**
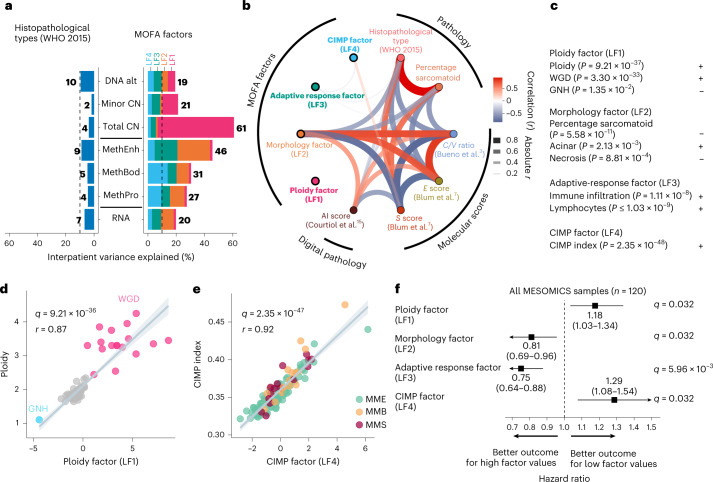
MOFA of whole genomes, transcriptomes and methylomes of the MESOMICS cohort (*n* = 120). **a**, Proportion of interpatient variance within a molecular layer explained by WHO-defined histopathological type (left) and MOFA latent factors 1–4 (right). For example, 7% of variation present in RNA expression can be explained by mesothelioma types, in contrast with 20% explained by integrative MOFA. CN, segmental copy number; DNA alt, rearrangements and mutations; MethBod, DNA methylation level at body regions; MethEnh, DNA methylation level at enhancer regions; MethPro, DNA methylation level at promoter regions; RNA, gene expression level. **b**, Network of the correlations between latent factors, tumor histopathology and previously published molecular scores. The arc colors, widths and transparency correspond to Pearson correlation coefficients. Features uncorrelated with any other features are highlighted in bold. AI, artificial intelligence; C/V ratio, log2 ratio of *CLDN15* to *VIM* gene expression; S score, sarcomatoid gene expression score; E score, epithelioid gene expression score. **c**, Interpretation of MOFA latent factors. Plus signs indicate positive correlations and minus signs indicate negative correlations. **d**, Correlation between the ploidy factor (LF1) and ploidy. **e**, Correlation between the CIMP factor (LF4) and CIMP index. The samples are colored by histological type. **f**, Forest plot of the hazard ratios of MOFA latent factors for overall survival. The squares correspond to estimated hazard ratios and the segments correspond to their 95% confidence intervals. In **b**–**e**, *P* values, *q* values and *r* coefficients were determined by two-sided Pearson correlation tests. In **d** and **e**, the gray bands represent 95% confidence intervals. Source data

**Extended Data Fig. 1:**
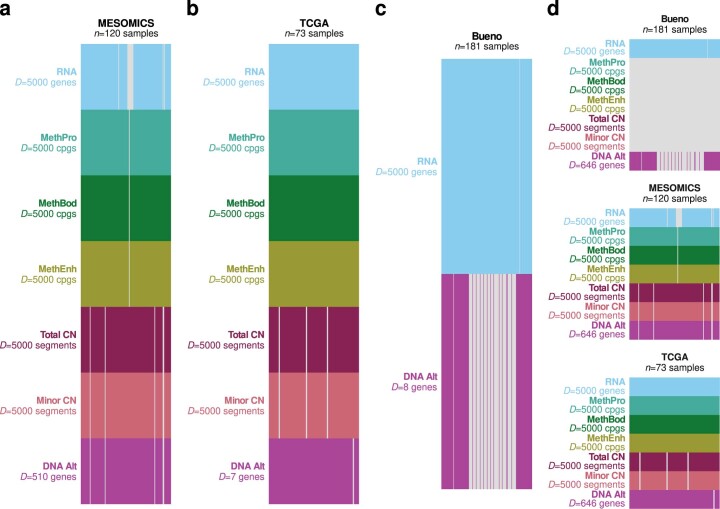
Overview of the MPM datasets for multiomic integration with MOFA. Overview of the omic data sets integrated into multiomics factor analyses (MOFAs), for (**a**) a MOFA of the MESOMICS cohort (*n* = 120), (**b**) MOFA of the TCGA cohort (*n* = 73), (**c**) MOFA of the Bueno cohort (*n* = 181), and (**d**) MOFA of the 3 cohorts (*n* = 120 + 73 + 181). *D* is the number of integrated omic features from genomic (rearrangements and mutations within *DNA Alt*; allele-specific copy number (CN) in *Total CN* and *Minor CN*), transcriptomic (*RNA*), and epigenomic data at promoter (*MethPro*), gene body (*MethBod*), and enhancer regions (*MethEnh*). Source data

**Extended Data Fig. 2:**
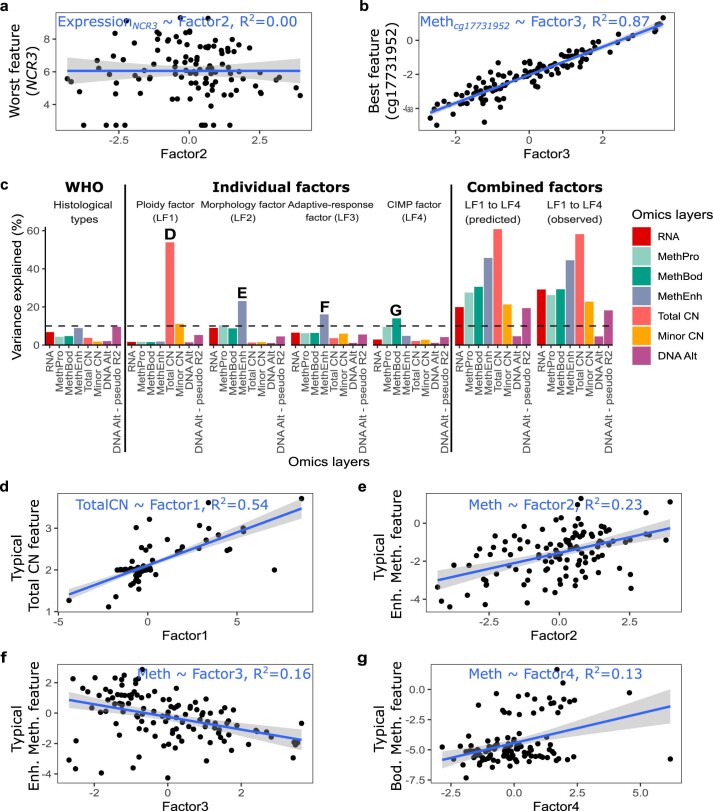
Proportion of interpatient variance explained by MOFA latent factors. **a**) Example feature for which a latent factor explains 0% of interpatient variance (here factor 2 explains no variance at all in the expression of gene *NCR3*—*R*^2^ = 0). **b**) Example feature for which a latent factor explains most of the variance (here factor 3 explains 87% of the variance of methylation site *cg17731952*—*R*^2^ = 0.87). **c**) Variance explained by the three histopathological types, each latent factor (LF) independently, predicted total variance explained by all latent factors together if they were completely independent (LF1 to LF4 predicted), and actual variance explained by a model including the four latent factors as covariables (LF1 to LF4 observed). CIMP: CpG island methylator phenotype. **d**) Typical Total copy number (CN) feature associated with Factor 1. **e**) Typical Enhancer Methylation feature associated with Factor 2. **f**) Typical Enhancer Methylation feature associated with Factor 3. **g**) Typical Gene Body Methylation feature associated with Factor 4. In (**a**)-(**b**) and (**d**)-(**g**), the gray band corresponds to 95% confidence intervals. Source data

**Extended Data Fig. 3:**
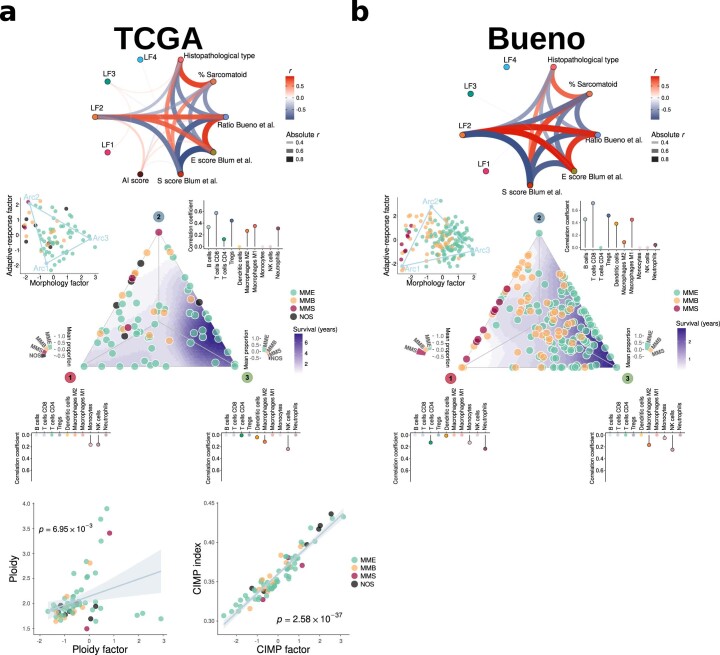
Replication of MOFAs latent factors and tumor tasks in major MPM cohorts. MESOMICS MOFA latent factors and tumor task replicated in the TCGA (**a**) and Bueno (**b**) cohorts. The gray band corresponds to 95% confidence intervals. In (**a**), *P* values correspond to Pearson correlation tests (*n* = 73). MME: epithelioid; MMB: biphasic; MMS: sarcomatoid; NOS: malignant pleural mesothelioma not otherwise specified. Source data

We first found that the current histopathological classification only accounts for up to 10% of the interpatient molecular differences (2–10%, depending on the molecular layer, with an average of 6%), leaving 90% unexplained (Fig. 1a). We then undertook an unsupervised decomposition of the interpatient molecular heterogeneity using Multi-Omics Factor Analysis (MOFA)^14^, integrating genomic, transcriptomic and epigenomic data. We identified four independent and reproducible latent factors individually explaining more than 10% of molecular variation in at least one molecular layer, and collectively up to 61% of interpatient differences (19–61%, depending on the molecular layer, with an average of 33%; Fig. 1a, Extended Data Figs. 1–3, Supplementary Fig. 1 and Supplementary Tables 4–7). Only latent factor 2 (LF2) was associated with the histopathological classification, the recent artificial intelligence score based on digital pathology^15^ and the previously proposed molecular classifications^3–7^ (median *q* value = 6.94 × 10^−11^; Fig. 1b). Therefore, LF1, LF3 and LF4 capture three prominent sources of biological variation overlooked by previous histopathological and genomic studies.

**Extended Data Fig. 4:**
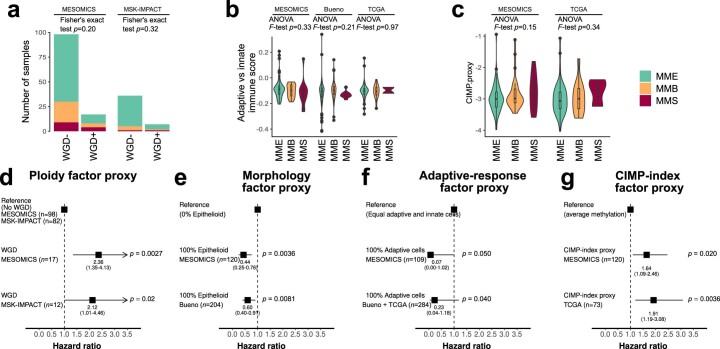
Replication of the prognostic value of MOFAs latent factors in other MPM cohorts. **a**)-(**c**) Association between histological types and proxies for the MOFA latent factors. **a**) Association between whole genome-doubling (WGD) status and histological types in the MESOMICS and MSK-IMPACT cohorts, as determined by Fisher’s exact tests. **b**) Association between the Adaptive versus innate response score and histological types, in the MESOMICS (*n* = 120) and Bueno (*n* = 211) and TCGA (*n* = 73) cohorts, as determined by ANOVA tests. **c**) Association between the CIMP-index proxy computed on a five-gene panel and histological types in the MESOMICS and TCGA cohorts, as determined by ANOVA tests. **d**)-(**g**) Forest plots of hazard ratios for overall survival showing the replication of latent factors’ prognostic value, using a Cox proportional hazards model. In (**b**)-(**c**), boxplots represent the median and interquantile range and whiskers the maximum and minimum values, excluding outliers. **d**) WGD status (proxy for the ploidy factor) in the MESOMICS and MSK-IMPACT cohorts. **e**) Percentage of epithelioid estimated by pathologists from H&E slides (proxy for the morphology factor) in the MESOMICS and Bueno cohorts. **f**) Adaptive versus innate response score (proxy for the adaptive-response factor), in the MESOMICS and Bueno and TCGA cohorts, computed as the difference between the proportion of lymphocyte B and T-cells minus the proportion of macrophages, monocytes, and neutrophils, estimated from gene expression data (quanTIseq software). **g**) CIMP-index proxy computed on a five-gene panel (proxy for the CIMP-index factor), in the MESOMICS and TCGA cohorts. In all panels, *P* values indicate the significance of tests. In (**d**)-(**g**), squares correspond to estimated hazard ratios and segments to their 95% confidence intervals; tests in the MESOMICS cohort (discovery) are two-sided while tests in validation cohorts (MSK-IMPACT, TCGA, or Bueno cohorts) are one-sided, in the direction found in the discovery cohort. Source data

LF1 (the ploidy factor) is largely explained by tumor ploidy (*r* = 0.87; Fig. 1c,d). LF2 (the morphology factor) separates the main histopathological types and thus summarizes the morphological and related molecular classifications (Fig. 1a–c). LF3 (the adaptive response factor) summarizes immune infiltration with adaptive response effectors (lymphocytes) (Fig. 1c). For LF2 and LF3, enhancer methylation was the major molecular layer captured (Fig. 1a), partly explained by its implication in the tumor–immune interaction phenotype captured by LF3, and its variability in MPM samples is probably driven by cell-type heterogeneity (Supplementary Fig. 2 and Supplementary Tables 5, 6 and 8). The major feature captured by LF4 (the CpG island methylator phenotype (CIMP) factor) was methylation at gene body and promoter regions, and most of its molecular variation was strongly associated with the CIMP index (*r* = 0.92; Fig. 1c,e). We then identified proxies to facilitate the interpretation of the latent factors and their implementation in the clinical setting: aneuploidy for LF1; the percentage of sarcomatoid component as reported by pathologists for LF2; an adaptive versus innate immune response score (Methods) for LF3; and a five-gene CIMP index proxy (Methods) for LF4. LF1, LF3, LF4 and their proxies were statistically independent of histopathological type (that is, all histological types can be either high or low ploidy, have high or low adaptive immune responses and have a high or low CIMP index), further confirming that these latent factors represent independent sources of molecular variation (Extended Data Fig. 4a–c).

In line with our previous observations^6^, tumor samples did not form clusters in MOFA but rather gradients between extreme molecular profiles (Fig. 1d,e). The ploidy factor ranged between a genomic near-haploidization (GNH) and a whole-genome doubling (WGD) profile, with a gradient of intermediate ploidies due to various levels of chromosome arm and focal amplifications and deletions (Fig. 1d). In contrast with the features found associated with the GNH subtype identified in the The Cancer Genome Atlas (TCGA) cohort^4^, the single near-haploid sample, MESO_108, had a ploidy of 1.10, almost no copy-neutral loss of heterozygosity (LOH) (<1%) and no *SETDB1*/*TP53* mutations and did not undergo WGD. Therefore, this sample does not correspond to the GNH subtype as described by Hmeljak and colleagues^4^, but to another possible genomic trajectory, where genomic instability is driven by alternative pathways. Differential gene expression analyses showed that, as reported in other tumor types^12^, the most upregulated enriched pathway in WGD-positive (WGD^+^) versus WGD-negative (WGD^−^) cases was E2F targets (*q* value = 0.048; Supplementary Tables 9 and 10), although we could not replicate this result in the TCGA cohort^4^, possibly due to the difficulty of replicating such findings in low-sample-size series (*n* = 11 WGD^+^ samples). The CIMP factor also ranged between two extreme profiles: CIMP-low and CIMP-high (Fig. 1e). A well-known effect of the CIMP-high phenotype is epigenetic silencing of tumor suppressor genes^16^. In line with this, we identified five Catalogue of Somatic Mutations in Cancer (COSMIC) tumor suppressor genes^17^, whose expression was negatively correlated with both the CIMP index and the methylation level of their CpG island(s): *CBFA2T3*, *FBLN2*, *PRF1*, *SLC34A2* and *WT1* (median *q* value = 2.6 × 10^−3^; Supplementary Table 11).

**Extended Data Fig. 5:**
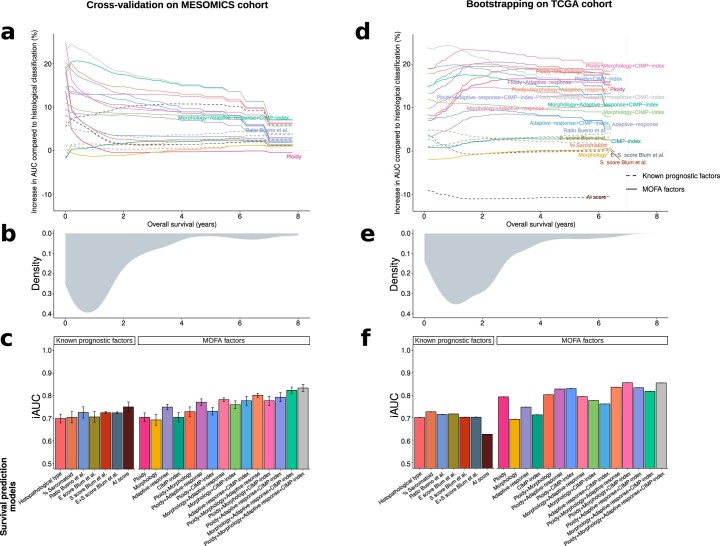
Performance of MOFA factors to predict survival. **a**) Increase in area under the curve (AUC) as a function of percentage of change compared to histological classification. **b**) Density of survival time within the MESOMICS cohort. **c**) Integral AUC (iAUC) of twenty-two Cox proportional hazards survival models based on: (i) the three histopathological types (MME, MMB, and MMS); (ii) the proportion of sarcomatoid content; (iii) the log2 ratio of CLDN15/VIM (C/V) expression proposed by Bueno and colleagues; (iv), (v) and (vi) the E score, S score, and combining both scores from Blum and colleagues, respectively; (vii) an Artificial Intelligence (AI) prognostic score; (viii-xi) the one-dimensional summary of molecular data using LFs as a continuous variable; (xii-xvii), the two-dimensional summary of molecular data using either each combination of 2 LFs as continuous variables, respectively; (xviii-xxi), the three-dimensional summary of molecular data using each combination of 3 LFs as continuous variables; and (xxii), the four-dimensional summary of molecular data using all 4 LFs. Bars represent the mean values and error bars their standard error. Panels (**a-c**) present the out-of-sample accuracy within the MESOMICS cohort (4-fold cross-validation on *n* = 120 individuals), while (**d-f**) present the out-of-sample accuracy within the TCGA cohort (2000 bootstraps on *n* = 73 individuals). The model fit accuracy (no split between training and test sets) on MESOMICS and TCGA cohort are presented in Supplementary Table 17. Source data

We trained latent factor-based survival models and tested their performance over previously proposed prognostic factors to evaluate to what extent each latent factor captured variability predictive of prognosis (Methods). While individually they provided a prediction value similar to each other, when combining the four latent factors there was an increase in their area under the receiver operating characteristic curve value, suggesting that they capture molecular characteristics with independent prognostic value, being informative of MPM progression in a complementary manner (Extended Data Fig. 5, Supplementary Fig. 3 and Supplementary Tables 12–20). In line with evidence from multiple cancer types^12^, survival was lowest for the greatest ploidy (Fig. 1f). As expected, samples in the lower extreme of the morphology factor, enriched for sarcomatoid tumors, presented the worst prognosis. The adaptive response factor linked hot tumors (tumors with a high level of immune infiltration) with better survival, whereas CIMP-low tumors had better survival than CIMP-high tumors (Fig. 1f). The previously described proxies also demonstrated prognostic value in the MESOMICS cohort, and allowed for validation of the prognostic value of the latent factors in the validation cohorts (Extended Data Fig. 4d–g). Probably due to the limited power and a potential effect of histology, the prognostic value of the ploidy and CIMP factors was not statistically significant when analyzing MME samples only; however, their respective effect size remained similar to those identified in the entire cohort (Supplementary Fig. 3). We additionally validated the existence of the four dimensions as well as their prognostic values in previously published cohorts (Supplementary Tables 21 and 22).

**Extended Data Fig. 6:**
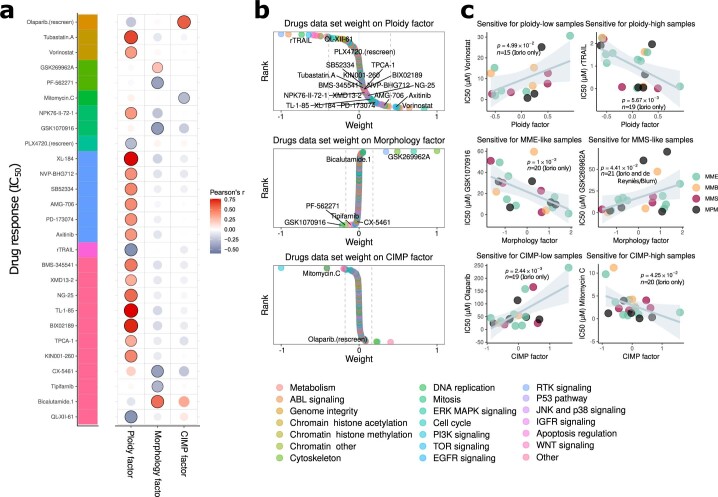
MOFA LFs of MPM cell lines and drug response. **a**) Correlations between drug responses (measured by half maximal inhibitory concentration, IC50 in μM) and MOFA LFs of cell lines. Significant associations are annotated by black point border. **b**) Distribution of drug response weights from the Drugs data set, with drugs for which the response is significantly correlated with the given LF annotated in black. Targeted pathways are represented in (**a**) by a color bar (left), and in (**b**) by point colors. **c**) Correlations between representative drug responses significantly correlated with MOFA LFs from cell lines (left: negative correlations, right: positive associations). MPM: malignant pleural mesothelioma not otherwise specified. Gray bands correspond to 95% confidence intervals. Pearson correlation coefficients and the associated two-sided *P* values are displayed in (**a**) and (**c**). Source data

Finally, combining molecular and drug response data for 59 MPM cell lines from Iorio et al.^18^, de Reyniès et al.^5^ and Blum et al.^7^, we were able to evaluate the therapeutic value of the ploidy, morphology and CIMP factors (the lack of microenvironment in cell culture models did not allow for replication of the adaptive response factor), by assessing the impact that cell line position along each latent factor had on the response to candidate drugs (Extended Data Fig. 6, Supplementary Fig. 4 and Supplementary Tables 23–26). Significant drug responses associated with the different factors were entirely orthogonal (Extended Data Fig. 6a), highlighting the fact that MOFA latent factors capture independent axes of heterogeneity in both tumoral mechanisms and therapeutic responses. Therefore, both survival and cell line analyses showed that these axes of variation are clinically relevant and have the potential for translation into clinical practice.

### Task specialization analyses reveal diverse tumor strategies

**Fig. 2:**
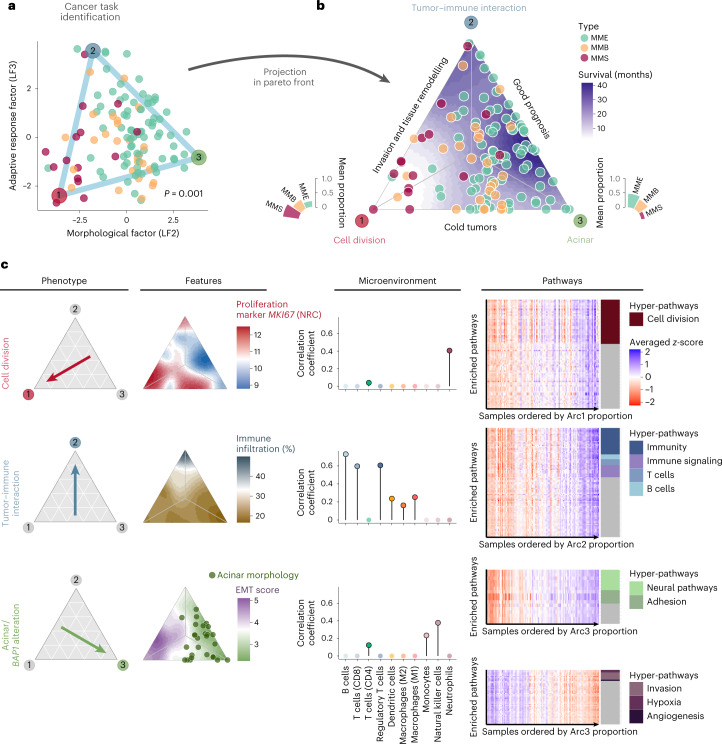
Cancer task inference from the morphology and adaptive response factors (*n* = 120). **a**, Sample positions along the morphological (LF2) and adaptive response factors (LF3) are contained within a triangle formed by three phenotypic archetypes (colored vertices). The *P* value corresponds to a one-sided test from the Pareto fit. **b**, Ternary plot representing the sample’s distance from the three specialized profiles. The bar plots represent the association between archetypes and histopathological types. **c**, Summary table of the main phenotypes, features and overexpressed pathways (columns) identified in each profile (rows). Left, arrows indicate the focal profile of each row. Middle left, ternary plots with a color-filled background representing key features for each profile. NRC, normalized read count. Middle right, lollipop plots presenting the correlation between RNA-seq-estimated immune cell infiltration and the proportion of archetypes. Right, expression heatmaps of cancer tasks inferred from each phenotype. The rows represent enriched pathways and the columns represent the samples, ordered by increasing phenotype proportion. The heatmap color scale corresponds to the averaged *z* score of each gene set. The colored tiles on the right annotate the gene sets that belong to the hyper-pathways inferred from each phenotype. Source data

Samples along the interdependent morphology and adaptive response factors formed a triangular shape delimited by three extremes (Fig. 2a and Supplementary Fig. 5). The well-established Pareto optimum theory^19^ (ParetoTI method) predicted that this pattern results from natural selection for cancer tasks, with specialist tumors close to the vertices of the triangle and generalists in the center (triangle fit *P* value = 0.001; Fig. 2b). Integrative gene set enrichment analysis (IGSEA) pointed to the following cancer tasks and tumor phenotypes: cell division, tumor–immune interaction and acinar phenotype (Fig. 2c and Supplementary Tables 27–30 for archetypes, IGSEA significant pathways and *q* values).

Tumors specialized in the cell division task displayed upregulation of these pathways, as reported by Hausser et al. in multiple tumor types^20^. This phenotype was enriched for nonepithelioid tumors and presented higher levels of necrosis, higher grade and a greater percentage of infiltrating innate immune response cells (neutrophils) (median *q* value = 0.005). Cell division specialization was supported by high expression levels of the proliferation marker *MKI67* and increased genomic instability (estimated from genomic, transcriptomic and epigenomic data; median *q* value = 1.97 × 10^−4^). Tumors specialized in the tumor–immune interaction task carried upregulated immune-related pathways, high expression of immune checkpoint genes and high immune infiltration with an enrichment for adaptive response cells: B lymphocytes, CD8^+^ T cells and regulatory T cells (median *q* value = 2.73 × 10^−3^). The cell division and tumor–immune interaction specialists also showed high expression of hypoxia response pathways and common enrichment for pathways in the invasion and tissue remodeling universal cancer task. Indeed, we found a higher epithelial-to-mesenchymal transition (EMT) score among tumors in this area of the Pareto triangle, driven by upregulation of mesenchymal genes and hypomethylation of their associated enhancers (median *q* value = 1.61 × 10^−6^). In line with in vitro studies showing that asbestos may induce EMT in MPM^21^, we found a positive correlation between the expression of mesenchymal genes and asbestos exposure score (*r* = 0.44 and *q* value = 0.01) and a negative correlation between this score and enhancer methylation of mesenchymal genes (*r* = −0.33 and *q* value = 0.02). We also observed overexpression of neoangiogenesis-related genes, corroborating the ability of these tumors to remodel their environment.

The last extreme phenotype was characterized by samples with acinar morphology, presenting a very structured tissue organization with epithelial cells tightly linked into tubular structures, and correlated with the presence of monocytes and natural killer cells (innate immune response cells) (median *q* value = 0.022). This phenotype presented the lowest EMT score, with overexpression of epithelial markers such as cell adhesion molecules (median *q* value = 1.21 × 10^−3^), corroborating the importance of tissue organization in this phenotype, and also low levels of *MKI67* expression, indicating slow growth. This phenotype showed no particular tumoral specialization in any task based on the few IGSEA upregulated pathways. In line with the better prognosis reported for this subtype^8^, the acinar phenotype is characterized by the highest levels of global methylation^22^ (*q* value = 5.58 × 10^−10^). Altogether, these data provide a biological understanding of the molecular and phenotypic heterogeneity characteristic of MPM tumors.

### WGS uncovers a diverse genomic landscape

**Fig. 3:**
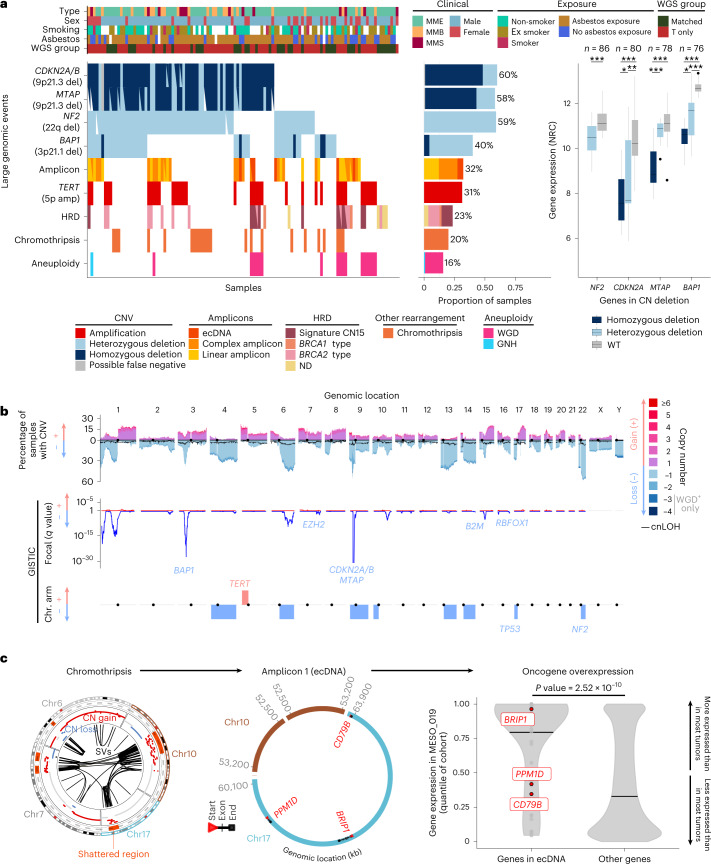
Genomic characterization of MPM from the MESOMICS cohort. **a**, Recurrent large genomic events. Top, clinical, epidemiological, morphological and technical features per sample. T only represents samples with WGS on the tumor sample only. Bottom left, oncoplot describing the genomic events per sample. amp, amplification; del, deletion; ND, HRD type not determined. Bottom middle, barplot of the frequency of each event within the cohort. Bottom right, comparison of the gene expression of cancer-relevant genes belonging to frequent deletions detected by GISTIC, with regards to their copy number (CN) status. Wild-type (WT) cases correspond to samples without copy number, structural variant or single-nucleotide variant events detected. The box plots represent the median and interquantile range and the whiskers the maximum and minimum values, excluding outliers. The *n* number above represents the number of biologically independent samples for each test. *0.01 < *q* value ≤ 0.05; **0.001 < *q* value ≤ 0.01; ***q value ≤ 0.001. NRC, normalized read count. **b**, Cohort-level copy number profile (top), with significantly altered regions identified by GISTIC in focal peaks (middle) and at the chromosome (chr.) arm level (bottom). cnLOH, copy-neutral LOH. **c**, Data from a patient with oncogene amplification due to a chromothripsis event (MESO_019). Left, chromosomes involved in the chromothripsis event (outer circle, shattered regions; intermediate circle, copy number gain and loss; inner circle, structural variants (SVs)). Middle, reconstructed ecDNA structure. Right, gene expression in MESO_019 relative to the expression in other tumors of the cohort (quantile). Oncogenes found within the ecDNA region are represented in red. The *P* value was determined by two-sided Wilcoxon rank-sum test. kb, kilobases. Source data

We found 97% (111/115) of MPM tumors harboring at least one large genomic event (copy number variant (CNV), amplicon, homologous recombination deficiency (HRD), chromothripsis or aneuploidy; Fig. 3a). As captured by the ploidy factor, MPM samples ranged from haploid to tetraploid (Fig. 1d). The average CNV profile was highly consistent between cohorts (Supplementary Fig. 6), with several recurrent chromosome arm-level CNVs, as well as focal alterations encompassing known cancer genes (Fig. 3b and Supplementary Tables 31–35). As previously reported^23^, all of the *MTAP* alterations co-occurred with *CDKN2A*/*B* (Fig. 3a and Supplementary Tables 36 and 37). We also found recurrent deletions of a prominent immune recognition gene, *B2M* (chr15q14; Fig. 3b).

**Extended Data Fig. 7:**
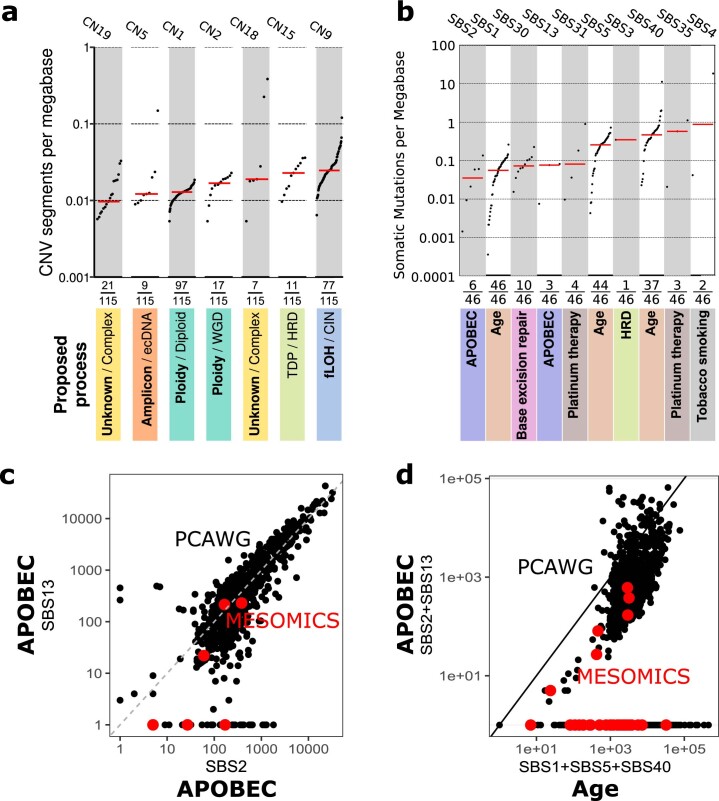
Tumor burden of mutational signatures. Tumor Mutational Burden of **a**) 7 copy number signatures (*n* = 115 biologically independent samples) and **b**) 10 Single Nucleotide Variant Signatures detected in the MESOMICS cohort (*n* = 46 biologically independent samples). Note that although SBS40 is associated with age in many cancers, its etiology is still unknown. TDP: tandem duplicator phenotype; HRD: homologous recombination deficiency; fLOH: focal loss of heterozygosity; CIN: chromosomal instability. **c**) Comparison of the tumor mutation burden (TMB, in number of mutations) of APOBEC signatures SBS2 and 13 in the MESOMICS cohort and in more than 2000 tumors from the Pan-Cancer Analysis of Whole Genomes (PCAWG) cohort. **d**) Comparison of the Relative TMB in number of somatic mutations of age-related signatures SBS1, SBS5, and SBS40, with that of APOBEC signatures SBS2 and 13 in the MESOMICS (red) and PCAWG cohorts (black). Source data

A comprehensive analysis of mutational signatures, encompassing single-base substitutions, CNVs and structural variants^24,25^, allowed us to identify the processes leading to particular somatic alteration patterns (Extended Data Fig. 7). A total of ten active single-base substitution signatures were detected in MPM genomes (Extended Data Fig. 7b); all corresponded to known COSMIC signatures and none was associated with asbestos exposure, as was previously reported^3,4^. Six tumors were found to have extrachromosomal DNA (ecDNA) (Supplementary Fig. 7 and Supplementary Table 38), and in the one sample with transcriptomic data we found increased expression of the genes predicted to be present on the ecDNA, including the known oncogene *BRIP1* (Fig. 3c). We observed that the aforementioned ecDNA sample co-occurred with, and may be fueled by, kataegis^26^ (Supplementary Fig. 8). Overall, kataegis was rarely seen in our cohort, contributing to only 2% of the MPM clustered mutations (Supplementary Tables 39 and 40). The identified complex mutational processes included a pattern compatible with chromothripsis. This was observed in 20% of the samples (Fig. 3a, Supplementary Fig. 9 and Supplementary Table 39) and also at the transcriptomic level, as fusion transcripts, in half of the positive samples (Supplementary Fig. 10a and Supplementary Tables 41–43). A signature of clustered structural variants was detected and significantly associated with a high structural variant load and chromothripsis (Supplementary Fig. 10b,c and Supplementary Tables 41 and 42). For one sample (MESO_019), the chromothripsis region overlapped with an ecDNA region, suggesting that chromothripsis may have been the source of the circular amplification (Fig. 3c). Finally, 23% of the samples showed a HRD phenotype, identified either by copy number signatures^25^ or structural variant pattern-based methods^27^ (Supplementary Fig. 11 and Supplementary Table 40). Among these samples, five harbored pathogenic germline mutations (from the ClinVar database) in one of 26 genes known to be involved in homologous recombination^28^—significantly more than the two mutations reported in the 77% of samples without HRD (Fisher’s exact test, *P* value = 0.00587).

We detected an HRD signature in nine out of 21 MPM cell lines from Iorio et al.^18^, thus validating the high rate of this pattern in MPM. In addition, the sensitivity of these cell lines to the clinically approved olaparib showed a tendency toward higher sensitivity in HRD samples compared with non-HRD samples (Supplementary Fig. 12). This may be linked with the results of a clinical trial suggesting a highly complex mechanism between the response to this drug and markers for DNA repair pathway activity^29^. Indeed, in contrast with their original hypothesis, patients with *BAP1* mutations had poorer survival when treated with olaparib than wild-type patients. In line with this observation, the olaparib response was positively associated with the prognostic CIMP index factor (*r* = 0.65; Extended Data Fig. 6), meaning that CIMP-low samples were more sensitive to this poly-ADP ribose polymerase inhibitor than CIMP-high samples (which are enriched for *BAP1* alterations (Fig. 5a) and associated with poorer survival (Supplementary Fig. 3)).

**Fig. 4:**
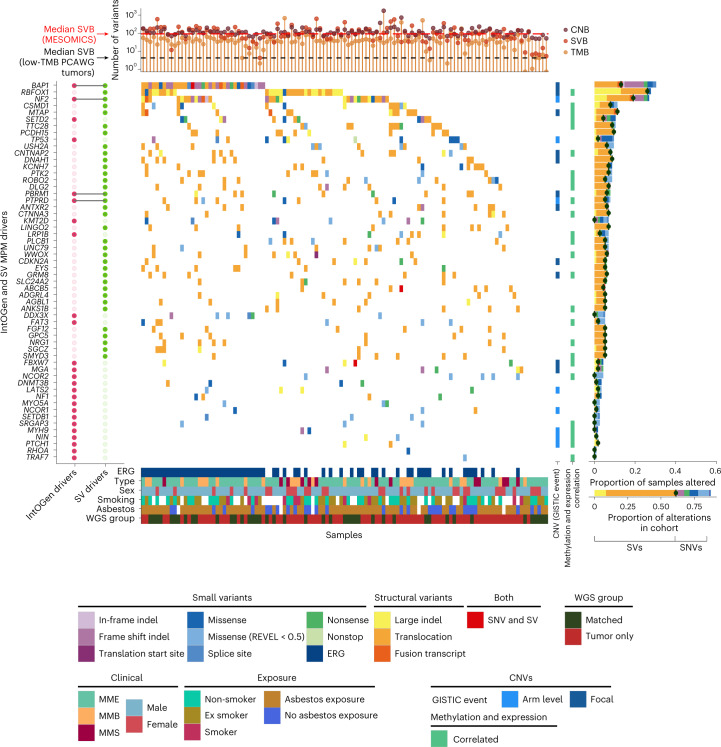
MPM driver genes in the MESOMICS cohort. Top, tumor mutational burden (TMB), number of segments or copy number burden (CNB) and structural variant burden (SVB) of each sample. Main, oncoplot describing genomic alterations in IntOGen and structural variant MPM driver genes per sample. These genomic events can co-occur with copy number changes. Large indels and translocations refer to structural variant events detected by structural variant callers while fusion transcripts are detected at the transcriptomic level. Each gene is also annotated as belonging to one focal or arm-level GISTIC event, as well as for being regulated by DNA methylation (right bars). Right, frequency of alterations within the cohort. For each gene, the dark green dot represents the frequency of structural variants. In the legend, ERG indicates whether the sample has one or more alteration in an ERG. Key clinical, epidemiological, morphological and technical features are given for each sample. PCAWG, Pan-Cancer Analysis of Whole Genomes; SNV, single-nucleotide variant; SV, structural variant. Source data

Despite the low mutational rate (0.98 nonsynonymous small variants per megabase; Supplementary Fig. 13a and Supplementary Tables 44–46), MPM tumors carry a particularly high number of structural variants relative to tumors with similarly low mutational burden (Fig. 4 and Supplementary Fig. 13b). The top genes altered by structural variants (≥5%) were *RBFOX1*, *NF2*, *BAP1*, *MTAP* and *PCDH15* (Supplementary Fig. 14a). For *RBFOX1*, 13 out of 39 samples have two separate events, with most deleting part of the RNA-binding protein domain (Supplementary Fig. 14b). Many of these genomic rearrangements resulted in fusion transcripts detected at the transcriptomic level (Supplementary Figs. 10a and 15).

Combining the MESOMICS dataset with the two other large datasets from Bueno et al.^3^ and the TCGA^4^, we reached the sample size (*n* ≈ 300) needed to detect rare driver alterations (1%). The IntOGen pipeline^30^ discovered 30 MPM driver genes based on small variants (Supplementary Fig. 14c). *BAP1*, *NF2*, *SETD2*, *TP53* and *LATS2* are all known MPM driver genes. Among the other 25 genes, some were previously reported as recurrently mutated in MPM (*PBRM1*, *KMT2D*, *DDX3X*, *PIK3CA*, *FBXW7*, *MGA*, *NF1*, *SETDB1*, *MYH9*, *PTCH1*, *RHOA* and *TRAF7*)^31–33^ or altered by structural variants (*PTPRD* and *LRP1B*)^34^, two were found overexpressed in MPM cell lines (*DNMT3B* and *EZH2*)^35^ and, for another two, germline mutations have been discovered, suggesting genetic susceptibility (*NCOR1* (ref. ^36^) and *MYO5A*^37^). The remaining seven driver genes have, to our knowledge, not been previously reported in MPM, but they are all known cancer genes, as reported in COSMIC: *FAT3*, *NIN*, *ARHGAP5*, *HLA-A*, *NCOR2*, *SRGAP3* and *WNK2*. Of note, *NF2* and *MYH9* (IntOGen drivers) are located within the significantly deleted chr22q region, along with *TTC28*—a gene frequently altered by structural variants (Figs. 3a,b and 4). Beyond extending the list of putative MPM drivers, combining point mutations with structural variants allowed for refinement of the frequency of alterations in key MPM genes (Fig. 4 and Supplementary Tables 41–46).

### Genomic alterations tune the molecular profiles of MPM

**Fig. 5:**
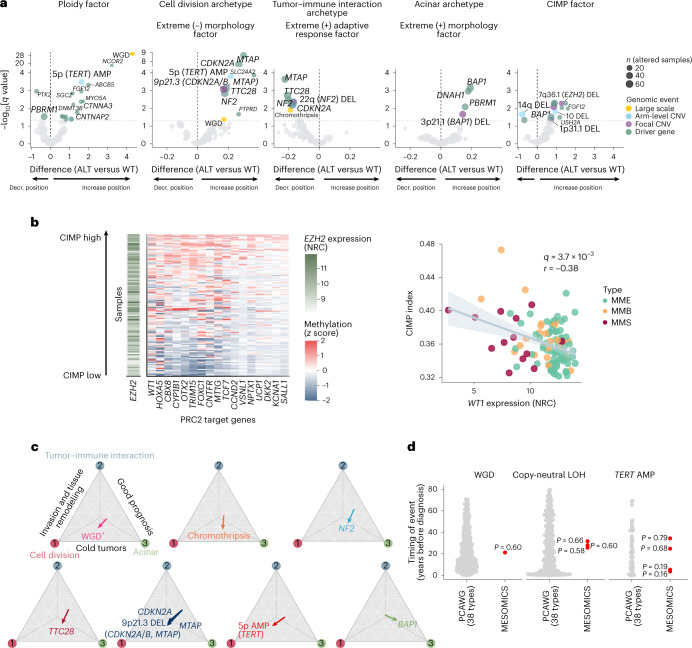
Impact of genomic events on MPM molecular profiles. **a**, Association between genomic events and MOFA factors. For each event, the ALT (altered) versus wild-type difference corresponds to the difference between the mean factor value of wild-type samples and that of altered samples. The *q* values correspond to an adjusted analysis of variance *P* value; the dashed horizontal line represents the *q* value threshold of 0.05. AMP, amplification; Decr., decrease; DEL, deletion. **b**, Association between CIMP index, *EZH2* expression (*n* = 109 samples) and PRC2 target gene methylation (*n* = 119 samples). Left, heatmap of *EZH2* gene expression (NRC) and CpG island methylation (*z* score) of PRC2 target genes whose methylation level was significantly positively correlated with CIMP index (*q* < 0.05), for samples ordered by CIMP index. Right: correlation between *WT1* expression and CIMP index. The *q* value was determined by Pearson correlation test and the gray band corresponds to the 95% confidence interval. **c**, Effect vector of key alterations affecting specialization in the tumor tasks from Fig. 2. The effect vector corresponds to the difference in position on the Pareto front between the centroid of altered samples and the centroid of wild-type samples. **d**, Comparison of the timing of large-scale amplifications in the MESOMICS and PCAWG cohorts. The points represent estimates of the timing of genomic events. The empirical *P* values (red data points) were determined by one-sided outlier tests. Source data

**Extended Data Fig. 8:**
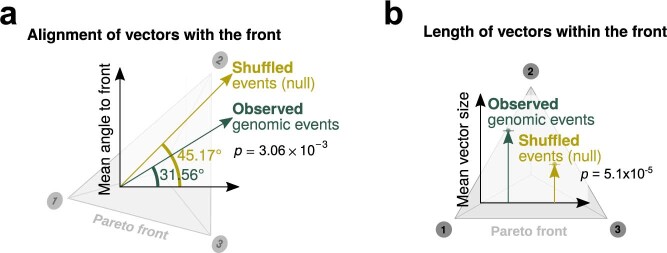
Test of the impact of genomic events on cancer task specialization. **a**) Alignment of vectors with the Pareto front in degree (0°: perfectly aligned, 90°: completely orthogonal) and (**b**) length of the vector. *P* values correspond to two-sided Wilcoxon tests between observed and shuffled vector distributions. Source data

Genomic events were associated with all MOFA latent factors and the extreme profiles that they encapsulated, as well as with the phenotypic specialists captured by the morphology and adaptive response factors (Fig. 5a and Supplementary Tables 47 and 48). Associated alterations significantly tuned tumor specialization (*P* value = 0.003; Methods and Extended Data Fig. 8). In addition to ploidy, *NCOR2* alterations and *TERT* amplification were associated with the ploidy factor (*q* values = 4.3 × 10^−18^ and 3.3 × 10^−4^, respectively; Fig. 5a). Thirty-six samples (31%) displayed *TERT* amplification, resulting in a significant increase in *TERT* expression (*P* value = 1.8 × 10^−5^; Supplementary Fig. 16a,b). *TERT* amplification was accompanied by an underlying amplification of chr5p in 81% of the positive cases. While no association was previously detected between *TERT* promoter mutations and WGD^38^, here we found that both *TERT* amplification and its increased expression were associated with WGD events (*P* value = 1.6 × 10^−10^ and 0.009, respectively; Supplementary Fig. 16c).

Genomic alterations in epigenetic regulatory genes (ERGs) have previously been shown to drive CIMP in cancer^39^. In line with this, we found enrichment for ERGs (*P* value = 3.4 × 10^−3^; Methods and Supplementary Fig. 17), including the mesothelioma drivers *NCOR2* and *EZH2*, among the genes highly expressed in CIMP-high tumors, and more generally in the list of MPM drivers (*q* value = 2.1 × 10^−5^). Chr7q36.1del, encompassing *EZH2*, further tuned the position of the samples along the CIMP factor (*q* value = 5.2 × 10^−3^; Fig. 5a). EZH2 (enhancer of zeste homolog 2) is a histone methyltransferase that functions as part of the Polycomb repressive complex 2 (PRC2) complex to promote gene silencing of specific targets^40^. Indeed, genes whose CpG island methylation level was highest in CIMP-high tumors were enriched for PRC2 target genes (*P* value = 0.01; Fig. 5b). *WT1*, which is found downregulated in CIMP-high tumors, is particularly interesting and a vaccine against this PRC2 target is currently being assessed in clinical trials for mesothelioma^41^. Cancers frequently associated with a CIMP-high phenotype include colorectal cancer (CRC) and glioma^42,43^, with *BRAF* (CRC) and *IDH1* (glioma) mutations also associated with this phenotype, as well as with microsatellite instability in CRC^42^. Microsatellite instability and *BRAF/IDH1* mutations were rare or absent events in our series and unrelated to the CIMP phenotype (Supplementary Tables 7, 44 and 49), suggesting that the mutational processes linked with CIMP phenotype in MPM may differ from those of other cancers.

WGD and chromothripsis seemed to push tumors away from the tumor–immune interaction phenotype (*q* values = 0.042 and 0.012, respectively; Fig. 5c); indeed, both cell division and acinar phenotypes were characterized by low immune cell infiltration (cold tumors), which may be explained by the downregulation of the interferon response pathway and *B2M* expression seen in WGD + MPM tumors (*q* value = 7.4 × 10^−17^; Supplementary Fig. 18a,b,e and Supplementary Tables 9 and 10). These may represent important mechanisms for WGD^+^ tumors to avoid the immune response^12,44^. Chromothripsis has also been associated with low immune infiltration as part of the chromosomal chaos that silences immune surveillance^45^.

*CDKN2A*, *MTAP* and *NF2* alterations also converged on cold tumors (median *q* value = 0.003). Within this cold phenotype, *TERT* amplification and alterations in *TTC28*, involved in the mitotic cell cycle, moved tumors towards cell division specialization (*q* values = 1.6 × 10^−4^ and 7.4 × 10^−4^, respectively; Fig. 5c), whereas chr3p21.1del (*BAP1*, *DNAH1* and *PBRM1*) and *BAP1* mutations moved tumors toward the better-prognosis acinar phenotype (*q* values = 0.021 and 7.1 × 10^−4^, respectively; Fig. 5c), as expected given the previously reported association between *BAP1* alterations and better survival in MPM^36^. A loss of BAP1 (BRCA1-associated protein-1) expression, measured by immunohistochemistry, was also associated with this phenotype (*r* = −0.38 and *q* value = 4.61 × 10^−5^; Supplementary Fig. 19). Interestingly, an analysis of splicing variation found that the morphology factor and acinar phenotype were significantly associated with alternative splicing events (Supplementary Fig. 20a–f). Major contributions came from events in cell adhesion genes, and neuronal progenitor BAF, neuron-specific BAF and SWI/SNF complexes, potentially affecting the alternative splicing pattern of genes such as *BCL11A* and *SMARCE1* (Supplementary Fig. 20g,h). The fact that these genes (just like *BAP1*) have important roles in chromatin remodeling suggests that disruption of chromatin remodeling pathways may molecularly define the acinar phenotype.

**Extended Data Fig. 9:**
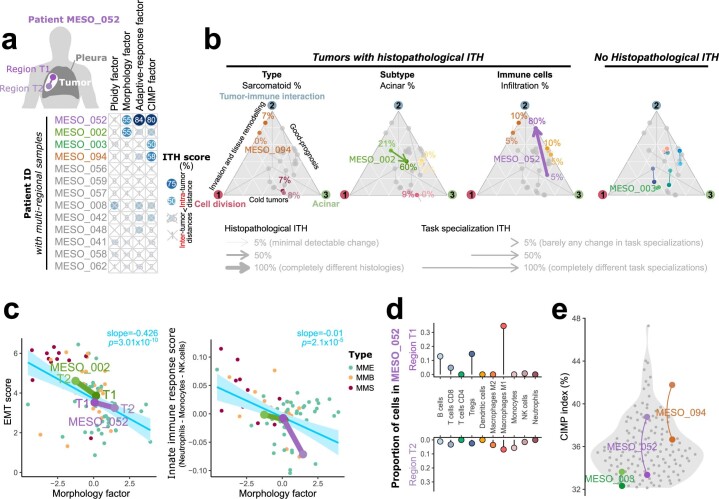
Multiomics intra-tumor heterogeneity (ITH) of 13 multiregion samples. **a**) Intratumoral heterogeneity (ITH) score, ranging from 0% (no ITH) to 100% (ITH greater than the maximum observed intertumor heterogeneity in the cohort), for each sample (row) and each MOFA latent factor (column). The score is computed as the percentage of inter-tumor distances in a MOFA factor that are lower than the observed intratumor distance between regions. The four samples with ITH score greater than 50% are highlighted in color. **b**) Relationship between histopathological heterogeneity and cancer task specialization. Ternary plots depicting task specialization in three cancer tasks (see Fig. 2). For each histopathological feature, a colored arrow connects regions from tumors with differences in this feature. Numbers correspond to the percentage of this feature in the tumor as estimated by our pathologist. The right ternary plot represents all samples with no histopathological ITH. **c**) Epithelial to mesenchymal transition (EMT) score and innate immune composition score as a function of MOFA’s Morphology factor. Small points correspond to all samples from the MESOMICS cohort, and large points connected by segments to regions from the 3 patients with CIMP factor ITH highlighted in (**a**). Blue bands correspond to 95% confidence intervals, and *P* values to two-sided *t*-tests. **d**) Lollipop plot of the estimated proportion of immune cells in two regions of a sample with ITH in the adaptive-response factor highlighted in (**a**). **e**) CIMP index in regions of two tumors with substantial ITH in the CIMP factor highlighted in (**a**) (colored points connected by an arc), compared to that of the rest of the cohort (grey points). Source data

The specialization of tumors can be influenced by early genomic events. Estimates of the timing of WGD, *TERT* amplification and copy-neutral LOH in the few samples (*n* = 6) with such events where a subclonal deconvolution was possible showed that our samples fall well within the values observed across >2,500 tumors of the Pan-Cancer Analysis of Whole Genomes Consortium^46^ (empirical *P* values = 0.16–0.79; Fig. 5d and Supplementary Fig. 21). Thus, these genomic events may indeed have occurred more than 10 years before diagnosis. Three out of the six patients were exposed to asbestos (of the other three patients, two had no known exposure and one had unknown exposure), among whom two had well-documented periods of exposure, from 56 to 21 years before diagnosis for MESO_048 (including the estimated timing of LOH) and from 54 to 50 years before diagnosis for MESO_057, more than 50 years before the estimated timing of *TERT* amplification, suggesting that genomic events can occur both concomitantly with and subsequent to asbestos exposure, although conclusive evidence of the timing of these alterations will need to be investigated in hypothesis-driven studies. Using a multiregional subcohort from 13 patients, we found intratumor heterogeneity in all factors except the ploidy factor, further suggesting that genomic events are mostly early and thus do not vary much across regions (Extended Data Fig. 9, Supplementary Fig. 22 and Supplementary Tables 50–52). Finally, we detected neutral tumor evolution close to the acinar phenotype (*P* value = 0.0024; Supplementary Fig. 23) at extreme values of the morphology and adaptive response factor, suggesting that tumors with this profile were even less influenced by recent genomic events.

## Discussion

The MESOMICS project represents a substantial advancement toward the comprehensive molecular characterization of MPM, made possible by inclusion of a large WGS dataset^3,4,34^ and by the depth of the multiomic integrative analyses undertaken. We demonstrated that ploidy, adaptive immune response and CpG island methylation constitute independent sources of molecular variation with quantitatively similar impacts on interpatient MPM heterogeneity as the histological classification. Despite some individual observations made in previous studies^6,7,13^, these three sources of molecular variation have been mostly unexplored or unknown because of the major focus that was put on refining the histological groups, and the lack of comprehensive analysis of a large multiomics dataset. In this sense, the unifying framework aspect of our research approach allowed us to capture the entire molecular landscape of MPM, summarized in four dimensions.

Aneuploidy is one of the morphology-independent features previously reported in MPM^4^ but poorly characterized. The ploidy factor identified tumors that underwent WGD, previously described in multiple cancer types as an early transformative event that dramatically destabilizes cell genetics and fuels tumor development^47^. WGD tends to be favored along the evolutionary course of low-mutational-burden tumors like MPM^12^ and is suspected to serve as a genetic spare tire in case of lethal alterations^48^. As a consequence, this event shapes the cellular phenotype associated with specific vulnerabilities^12^.

The CIMP has been reported in several cancer types, most notably CRC and glioblastoma, with inconsistent associations with survival^49–51^. Here we provide further evidence, to that of Blum et al.^7^, of distinct variation in CIMP index within mesothelioma tumors, and have shown that a high CIMP index is independent of morphology and predictive of poorer outcome. While a universal cause for a CIMP-high phenotype has not been established, it has been previously associated with alterations in ERGs^39,52^. Indeed, our data suggest that some mesothelioma tumors may acquire a CIMP-high phenotype through the activity of the ERG *EZH2*, to hypermethylate and silence specific target genes. Such a strategy may be warranted to promote malignant transformation in a lowly mutated tumor such as mesothelioma^35^.

Pareto task inference uncovered three specialized tumor profiles in the space delimited by the interdependent morphology and adaptive response factors, presumably resulting from pressures of the microenvironment, each selecting for adaptive alterations and phenotypic traits. Cell division specialists adopted a fast reproduction strategy that was expected to result from unfavorable and unpredictable environments^53^, with their genomic instability suggesting adaptation through evolutionary leaps^54,55^. Immune interaction specialists adopted an immune evasion or camouflage strategy. Both phenotypes also presented characteristics of invasion and tissue remodeling specialists^20^. These tumors tended to occur in intensely asbestos-exposed individuals, suggesting that chronic inflammation (promoted by asbestos exposure^56^) may have created the unfavorable environment responsible for selective pressure. Finally, acinar phenotype specialists adopted a structured tissue organization and slow growth strategy. This suggests an equilibrium strategy that is expected to be favorable in stable, resource-rich environments with limited predation^57^, in line with the lower level of asbestos exposure and limited inflammation and immune infiltration observed in these tumors. Consistent with limited environmental pressures, acinar tumors were enriched for neutral evolution and *BAP1* alterations—an event that, when combined with weak asbestos exposure in mice, greatly increased mesothelioma occurrence over weak asbestos exposure alone^58^.

**Fig. 6:**
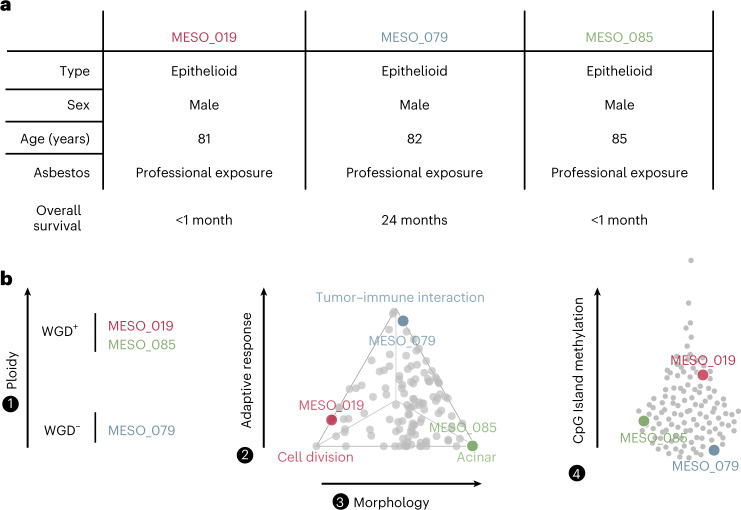
Added value of the four-factor molecular classification in understanding intertumor heterogeneity in three example patients. **a**, Patients MESO_019, MESO_079 and MESO_085 had nearly identical clinical characteristics. **b**, The three patients had vastly different profiles based on our four-factor morphomolecular classification: different WGD status (left), opposite positions on the Pareto front (middle) and variable levels of CpG island methylation (right). Source data

Overall, the four molecular factors are highly informative and capture specific profiles that are complementary in predicting tumor phenotype and aggressiveness. The fact that they are all independent and mostly unrelated to the morphology factor (histology) means that disregarding them might not only jeopardize the success of any treatment but also miss opportunities to stratify patients based on their molecular profile (Fig. 6). The tightly correlated proxies that we have identified could serve as biomarkers for response to specific therapies (such as immunotherapy for LF3) and could be easily tested in a hypothesis-driven study design. Subsequently, integrating these complementary factors would help to stratify patients for preselected-cohort clinical trials^59^, a process that has proven to be beneficial in small-cell lung cancer, another aggressive recalcitrant cancer^60–62^. The results of the MESOMICS project pave the way for the establishment of a more clinically relevant morphomolecular classification of MPM tumors.

## Methods

This section briefly describes the main methods (see Supplementary Information for details on the data, processing and analyses).

### Ethics

All of the methods were carried out in accordance with relevant guidelines and regulations. This study is part of a larger study, the MESOMICS project, aiming to perform comprehensive molecular characterization of MPM, and was approved by the International Agency for Research on Cancer (IARC) Ethics Committee (project number 15-17). The samples used in this study belong to the virtual biorepository French MESOBANK. Written, informed consent was obtained from all participants and no participant compensation was provided.

### Clinical data

Age at diagnosis (in years), sex (male or female), smoking status (nonsmoker, ex smoker or smoker), asbestos exposure (exposed or nonexposed), previous treatment with chemotherapy drugs (yes or no), treatment information (surgery, chemotherapy, radiotherapy, immunotherapy or cancer history) and survival data (calculated in months from surgery to the last day of follow-up or death) were collected for all 123 patients. The median age at diagnosis was 67.5 years and 73.3% of patients were male.

### MESOMICS cohort

The MESOMICS cohort includes biological material from 123 patients with MPM (including three nonchemonaive patients who were excluded from all analyses unless explicitly mentioned) kindly provided by the French MESOBANK and annotated with detailed clinical, epidemiological and morphological data. Samples were collected from chemonaive surgically resected tumors, applying local regulations and rules at the collecting site, and included patient consent for molecular analyses, as well as the collection of de-identified data. Samples underwent an independent pathological review by the French MESOPATH reference panel, who determined that of the 120 MPM tumor samples, 79 belonged to the MME type, 26 were MMB and 15 were MMS. Of the 105 samples with an epithelioid component (79 MME and 26 MMB), solid, acinar, trabecular and tubulopapillary architectural patterns were the most frequent in the series (*n* = 37, 31, 16 and 14, respectively).

### Discovery and intratumoral heterogeneity cohorts

Among the 123 patients with MPM, 13 had two tumor specimens collected for the study of intratumoral heterogeneity (ITH). The one with the highest tumor content, estimated by pathological review, was selected for this descriptive study and is reported in Supplementary Tables 1–3, and the other region is described in Supplementary Tables 50–52. Additionally, three patients have been reported as nonchemonaive and they were excluded from the analyses except if explicitly mentioned otherwise in the Methods.

### Pathological review

For all 136 samples (123 tumors plus 13 additional regions), a hematoxylin and eosin stain from a representative formalin-fixed, paraffin embedded block was collected for pathological review. Our pathologist (F.G.-S.) performed a detailed pathological review and classified all tumors according to the 2015 WHO classification^63,64^. The hematoxylin and eosin stain was also used to assess the quality of the frozen material selected for molecular analyses and to confirm that all frozen samples were at least 70% tumor cells.

### Artificial Intelligence analysis

Whole-slide image-based artificial intelligence prognostic scores were computed using the artificial intelligence MesoNet model based on morphological features, developed by Owkin—an artificial intelligence for medical research company^15^.

### Statistical analyses

All analyses were performed in R version 4.1.2. All tests involving multiple comparisons were adjusted using the Benjamini–Hochberg procedure, controling the false discovery rate using the p.adjust R function (stats package version 3.4.4). To limit false discoveries, we took a conservative *q* value threshold of 0.05. In addition, in line with the American Statistical Association statement on the misuse of *P* values^65^, which intends to ‘steer research into a “post *P* < 0.05 era"’, we report all *P* and *q* values, even those that may be closer to arbitrary thresholds such as the 5% threshold. To improve the reproducibility of our results, we summarize in Supplementary Tables 21 and 22 all *P* and *q* values reported in the text and main figures, along with details about the tests performed (hypothesis, model and sample size) and replication performed with additional cohorts.

### Survival analysis

Survival analysis has been performed using Cox’s proportional hazard model from which the significance of the hazard ratio between the reference and the other levels has been evaluated using Wald tests. We assessed the global significance of the model using the logrank test statistic (R package survival version 2.41-3) and drew Kaplan–Meier and forest plots using the R package survminer (version 0.4.2).

### DNA extraction

Included samples were extracted using the Gentra Puregene Tissue Kit (4 g) (158667; Qiagen), following the manufacturer’s instructions. All DNA samples were quantified using the fluorometric method (Quant-iT PicoGreen dsDNA Assay; Life Technologies) and assessed for purity by NanoDrop (Thermo Scientific) 260/280 and 260/230 ratio measurements. The DNA integrity of the fresh frozen samples was checked with a TapeStation system (Agilent Biotechnologies) using Genomic DNA ScreenTape (Agilent Biotechnologies).

### RNA extraction

Included samples were extracted using the AllPrep DNA/RNA extraction kit (Qiagen) following the manufacturer’s instructions. All RNA samples were treated with DNAse I for 15 min at 30 °C. The RNA integrity of the frozen samples was checked with a TapeStation system (Agilent Biotechnologies) using RNA ScreenTape (Agilent Biotechnologies).

Because of unsuccessful extraction (impacting either the quality or the quantity), we obtained different numbers of MPM samples for which WGS, DNA methylation or RNA sequencing (RNA-seq) data are available (Supplementary Tables 1–3).

### DNA sequencing

#### Sequencing

WGS was performed by the Centre National de Recherche en Génomique Humaine (Institut de Biologie François Jacob, CEA) on 130 fresh frozen MPMs, 54 of which with matched normal tissue or blood samples. We used an Illumina TruSeq DNA PCR-Free Library Preparation Kit (20015963; Illumina) according to the manufacturer’s instructions and sequenced them on a HiSeq X Five platform (Illumina) as paired-end 150-base pair reads. Samples paired with matched normal tissue or blood had a target sequencing depth of 60× and other samples had a target depth of 30×.

#### Data processing

WGS reads were mapped to the reference genome GRCh38 (with ALT and decoy contigs) using our in-house workflow (https://github.com/IARCbioinfo/alignment-nf; release version 1.0)^66^. In summary, this workflow relies on the Nextflow domain-specific language^67^ version 20.10.0.5430 and consists of four steps: read mapping (software BWA^68^; version 0.7.15), duplicate marking (software samblaster^69^; version 0.1.24), read sorting (software sambamba^70^; version 0.6.6) and base quality score recalibration using GATK^71^ (version 4.0.12).

#### Variant calling and filtering on DNA

We performed somatic variant calling using the software Mutect2 (ref. ^72^) from GATK version 4.1.5.0, as implemented in our Nextflow workflow (https://github.com/IARCbioinfo/mutect-nf; release version 2.2b). Multiregion samples were processed jointly using the multisample calling mode of Mutect2. We called germline variants using Strelka2 (ref. ^73^) version 2.9.10-0 using our Nextflow workflow (https://github.com/IARCbioinfo/mutect2-nf; release version 1.2a). Annotation was performed with ANNOVAR^74^ (16 April 2018) using the GENCODE version 33 annotation, COSMIC version 90 and REVEL databases. To call somatic variants on tumor-only samples (72/115), a similar procedure was performed (Mutect2 tumor-only mode) but including further germline-filtering steps using a random forest classifier.

#### CNV calling

Somatic CNVs were called using the PURPLE software^75^ version 2.52, as implemented in our Nextflow workflow (https://github.com/IARCbioinfo/purple-nf; version 1.0). We used a total of 57 matched WGS samples of MPM (including multiregion samples) for benchmarking the tumor-only mode of PURPLE. We ran PURPLE twice for each matched sample: first using the matched WGS normal/tumor pair as input and second using only the tumor WGS sample as input.

#### Structural variant calling

To identify somatic structural variants, including insertions, deletions, duplications, inversions and translocations, we built a consensus structural variants call set by integrating SvABA^76^ version 1.1.0, Manta^77^ version 1.6.0 and DELLY^78^ version 0.8.3 calls with SURVIVOR^79^ version 1.0.7. Somatic structural variants (minimum structural variant size = 50 base pairs) identified by at least two callers and single-caller predictions with a minimum read support of 15 pairs (including paired-end and split-read evidence) were included in the consensus set of each matched sample.

### RNA-seq

#### Sequencing

RNA-seq was performed on 126 fresh frozen MPM samples in the Cologne Center for Genomics, of which 109 MPM samples belonged to the discovery cohort (Supplementary Tables 1–3). Libraries were prepared using the Illumina TruSeq Stranded mRNA Sample Preparation Kit (20020595; Illumina) and the pool was sequenced using an Illumina NovaSeq 6000 sequencing device and a paired-end 100-nucleotide protocol.

#### Data processing

The 126 raw read files from the MESOMICS cohort and the 21 files from the Iorio and colleagues^18^ mesothelioma cohort (downloaded from the European Genome-phenome Archive (EGA) and Sequence Read Archive websites; datasets EGAS00001000828 and PRJNA523380, respectively) were processed in three steps using the RNA-seq processing workflow based on the Nextflow language and accessible at https://github.com/IARCbioinfo/RNAseq-nf (release version 2.3)^66^. Then, reads were realigned locally using ABRA2 (ref. ^80^); (workflow https://github.com/IARCbioinfo/abra-nf; release version 3.0) and base quality scores were recalibrated using GATK (workflow https://github.com/IARCbioinfo/BQSR-nf; release version 1.1). Once processed, expression was quantified using StringTie software (version 2.1.2; Nextflow pipeline accessible at https://github.com/IARCbioinfo/RNAseq-transcript-nf; release version 2.2).

The raw read counts of the 59,607 genes in the expression data matrix, from the MESOMICS, TCGA and Bueno cohorts^3,4^, from which we removed non-chimionaif samples, were normalized using the variance-stabilizing transform (vst function from R package DESeq2 version 1.14.1); this transformation enables comparisons between samples with different library sizes and different variances in expression across genes.

### DNA methylation

#### EPIC 850K methylation array

Epigenome analysis was performed on 119 MPMs (Extended Data Fig. 1 and Supplementary Tables 1–3), two technical replicates and three adjacent normal tissues. Epigenomic studies were performed at the IARC with the Infinium EPIC DNA methylation beadchip platform (Illumina) used for the interrogation of over 850,000 CpG sites (dinucleotides that are the main target for methylation).

#### Data processing

The resulting IDAT raw data files were preprocessed using the R packages minfi (version 1.34.0) and ENmix (version 1.25.1). Raw data were then normalized using functional normalization (function preprocessFunnorm; minfi), to reduce technical variation within the data, and probe removal steps were performed to ensure reliability and accuracy of the final dataset. This resulted in a normalized, filtered dataset of 781,245 probes for 139 samples. Finally, beta and *M* values were extracted (functions getBeta and getM; minfi). Nine probes recorded *M* values of −∞ for at least one sample, and these values were replaced with the next lowest *M* value in the dataset. The three normal tissues and one remaining technical replicate were then removed from the beta and *M* matrices for the subsequent analyses. This resulted in 135 samples: 122 for discovery and an additional 13 for ITH analyses.

#### CIMP index

A CIMP index value was calculated for all samples as follows. The mean beta value across all probes located within CpG islands was calculated per sample, resulting in beta values for 24,891 and 24,924 CpG islands, MESOMICS (EPIC array), TCGA^4^ and Iorio and colleagues^18^ cell lines (HM450K array), respectively. The CIMP index was then calculated as the proportion of these 24,891 or 24,924 islands with ≥30% methylation (beta value ≥ 0.3) per sample.

### Integrative unsupervised analyses

We performed four series of analyses with different subsets of samples: (1) discovery analyses with all of our discovery cohort (MESOMICS cohort; 120 samples), for which WGS, RNA-seq and/or 850K methylation array data were available; (2) and (3) replication analyses with the already published data from Bueno^3^ (181 samples after exclusion of nonchemonaive samples) and Hmeljak and colleagues^4^ (TCGA cohort; 73 samples in the curated list), respectively; (4) combined analyses integrating the MESOMICS, Bueno and TCGA cohorts^3,4^ with a total of 374 samples; and (5) replication combining cell lines from the Iorio study^18^ (for which whole-exome sequencing, expression arrays and RNA-seq, 450K methylation arrays and drug responses in the form of half-maximum inhibitory concentration scores are available (21 samples; 265 drugs)) and the de Reyniès^5^ and Blum et al.^7^ datasets (for which expression arrays and drug responses are available (38 samples; three drugs)). In addition, some single-omic analyses are also described in this section.

#### Preprocessing of expression data

We used normalized read count matrices (see the section ‘RNA-seq’) for subsets (1)–(4), encompassing 59,607 genes. Among these genes, those having less than one fragment per kilobase of exon per million mapped fragments (FPKM) difference across the samples were excluded from the unsupervised analyses. Also, to mitigate sex influence on the expression profiles, we removed genes from the sex chromosomes. For each analysis, the top 5,000 most variable genes were selected. Similarly, the 5,000 most variable genes from the normalized array expression of cell lines (see the section ‘Processing of publicly available expression array processing’ in Supplementary Methods) were selected. Whenever several probes were available for the same gene, the one with the highest intensity was selected.

#### Preprocessing of methylation data

DNA methylation was available for both the MESOMICS and TCGA cohorts. First, we extracted the *M* values of the CpGs from the MESOMICS, TCGA^4^, combined MESOMICS/TCGA and Iorio^18^ cell line cohorts, respectively^81^. We excluded sex chromosome CpGs, CpGs that did not pass quality control (see the section ‘DNA methylation’ in Supplementary Methods) and those having less than 0.1 beta value difference across the (1) 119, (3) 73, (4) 192 and (5) 59 samples. Based on this annotation, the CpG list representing the methylation data was divided according to their association with promoters, enhancers or the gene body using the EPIC 850K array manifest B5 (see the section ‘Regional methylation analysis’ in Supplementary Methods), resulting in three datasets, respectively named MethPro, MethEnh and MethBod. For each analysis and dataset, the top 5,000 most variable CpGs (calculated from *M* values) were selected.

#### Preprocessing of copy number changes

Copy number change data were available for the MESOMICS, TCGA and MPM cell line cohorts. We assessed the global (total) and minor (minor) allele copy number states at the gene level using, respectively, the total (total) and minor (minor) copy number estimate given by PURPLE (see the section ‘CNV calling’) on the hg38 genome for the MESOMICS cohort and SNP array estimates downloaded from the Genomic Data Commons portal for the TCGA–MESO cohort^4^ and from the Cell Model Passports portal for the MPM cell lines.

For the three analyses, the resulting value assigned to each gene is an average of the copy number estimate of the tumor by taking into account the tumor purity (purity) estimated by PURPLE. To avoid redundancy, genes with exactly the same resulting copy number value in all samples (because of their genome location proximity) were grouped as one single feature in the dataset. Only the genes or groups of genes altered in at least three samples were selected. To ensure continuity of the data, which is technically necessary for the algorithm, the copy number estimates were centered and scaled before being integrated into the MOFA algorithm. For consistency, somatic CNVs occurring on sex chromosomes were removed and the top 5,000 most variable genes or groups of genes were selected to be integrated.

#### Preprocessing of genomic alterations data

Somatic structural variants data were used only for integrative analyses (1) and (4), while somatic mutations were used in all analyses. Each gene, altered by somatic splicing, structural variants or exonic, damaging mutations (see the section ‘Damaging variants and driver detection’ in Supplementary Methods) was integrated in a common dataset. Of note, for missense mutations, we used the REVEL annotation included in ANNOVAR for predicting the pathogenicity of these variants and we used a 0.5 cut-off to restrict to the most likely damaging missense events. We also removed genes altered in fewer than three samples. For consistency, we selected genes in non-sex chromosomes, protein-coding or long noncoding RNA genes, and with expression greater than or equal to 0.01 fragment per kilobase of exon per million mapped fragments (FPKM) in at least one sample of the cohort, to be sure to include genes expressed in mesothelioma. We integrated the resulting datasets as a Boolean variable in the following analyses.

#### Multiomic integrative analyses

To provide an integrative low-dimensional summary of the molecular variation across the samples, we performed continuous latent factors identification using the software MOFA (R package MOFA2, version 1.7.0). Indeed, MOFA is able to integrate different molecular datasets (layers) by generating independent continuous variables, named latent factors, that explain most variation from the joint datasets. In total, we performed five analyses: (1) MOFA–MESOMICS (*n* = 120; Fig. 1 and Extended Data Fig. 1a); (2) MOFA–Bueno (*n* = 181; Extended Data Fig. 1c); (3) MOFA–TCGA (*n* = 73; Extended Data Fig. 1b); (4) MOFA–3 cohorts (*n* = 374; Extended Data Fig. 1d) and (5) MOFA–cell lines, as described above (*n* = 59; Supplementary Fig. 4). Additionally, we ran MOFA on our discovery cohort, including the ITH samples (MOFA–ITH; *n* = 134) to evaluate the ITH within MPM samples.

**Extended Data Fig. 10:**
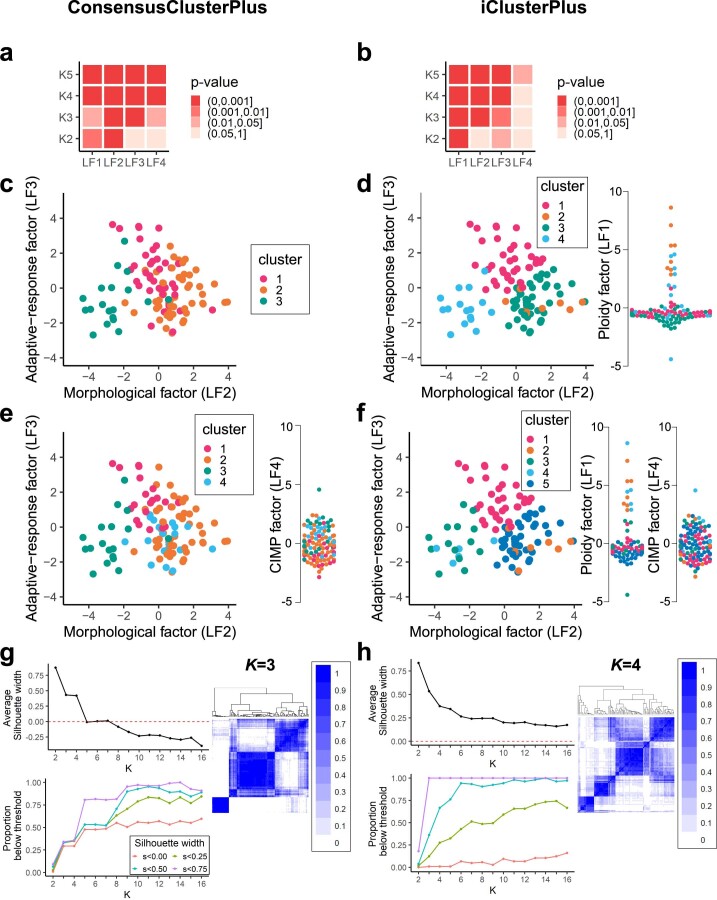
Association between MOFA latent factors and the clusters identified by consensus clustering (**a**-**d**) and integrative clustering (**e**-**h**). **a**) Kruskal-Wallis rank sum test significance (*P* value) between each *K* (row) and the LFs (column), for *K* from 2 to 5 from consensus clustering results and the first four LFs. **b**) Kruskal-Wallis rank sum test significance (*P* value) between each *K* (row) and the LFs (column), for *K* from 2 to 5 from integrative clustering results and the first four LFs. **c**) Consensus clustering results for *K* = 3. Samples are visualized in MOFA latent factor space of LF2 vs. LF3 and colored by the consensus clustering results. **d**) Integrative clustering results for *K* = 4. Samples are visualized in MOFA latent factor space of LF2 vs. LF3 and colored by the integrative clustering results. On the right, we show the samples in one-dimensional space of LF1 using beeswarm plot. **e**) Consensus clustering results for *K* = 4. **f**) Integrative clustering results for *K* = 5. Samples are visualized in MOFA latent factor space of LF2 *vs*. LF3 and colored by the integrative clustering results. On the right, we show the samples in one-dimensional space of LF1 and LF4 using beeswarm plot. **g**) Top-left: average silhouette width for consensus clustering with different *K*. Bottom-left: proportion of samples below the selected silhouette width threshold for consensus clustering with different *K*. Right: consensus matrix heatmap for *K* = 3. Color gradient represents consensus values from 0–1. **h**) Top-left: average silhouette width for integrative clustering with different *K*. Bottom-left: proportion of samples below the selected silhouette width threshold for integrative clustering with different *K*. Right: heatmap of the frequencies of samples being clustered together among all clustering results using the set of iClusterPlus lambda values for *K* = 4. Color gradient represents consensus values from 0–1. Source data

MOFA was performed independently for each analysis, setting the number of latent factors to ten (function runMOFA from the R package MOFA2). A summary of all of these runs is given in Extended Data Figs. 1 and 2, Fig. 1 and Supplementary Figs. 1 and 4 and coordinates and proportions of variance explained for models (1)–(4) are given in Supplementary Tables 4–8, while those for MOFA–ITH are given in Supplementary Tables 50–52 and those for the cell lines (model (5)) are given in Supplementary Tables 23–26. A comparison with other multiomic methods is provided in Extended Data Fig. 10 (see section 'Multiomic integrative analyses details' in Supplementary Methods).

### Evolutionary tumor trade-off analyses

#### Pareto task identification

The Pareto front model was fitted to different sets of samples using the ParetoTI R package (https://github.com/vitkl/ParetoTI; release version 0.1.13), following the above-mentioned analyses (1)–(4), and additionally on two different kinds of molecular maps: using MOFA (restricting to LF1, LF2, LF3 and LF4) and using expression principal component analysis as technical validation (see the section ‘RNA-seq’). In brief, the algorithm tries to find polyhedra by testing successively 1 to *n* axes, adding them one after another in decreasing order of transcriptomic variance explained. For this technical reason, the MOFA latent factors were ordered as follows by decreasing transcriptomic variance explained: morphology factor (LF2), adaptive response factor (LF3), CIMP factor (LF4) and ploidy factor (LF1). For each number *n* of axes used, ParetoTI identifies the position of the *n* + 1 = *k* vertices (archetypes) in the molecular map defined, and we used 200 bootstraps, each taking 75% of the data to measure the variability in archetype position and infer archetype positions robust to outliers (function fit_pch_bootstrap with the parameters bootstrap = T and bootstrap_N = 200; see our code at https://github.com/IARCbioinfo/MESOMICS_data/blob/main/phenotypic_map/MESOMICS/PhenotypicMap_MESOMICS.md).

#### Interpretation of tumor archetypes

To further characterize the phenotype of each archetype, we used the proportion of each archetype for each sample estimated by ParetoTI. These proportions were used as continuous variables to further test the association between each archetype and clinical, epidemiological and morphological variables, as well as molecular data (Supplementary Tables 27–30).

More specifically, we inferred each archetype phenotype by performing IGSEA on the expression data. To do so, we used the ActivePathways R package (https://github.com/reimandlab/ActivePathways; release version 1.1.0), which is a tool able to integrate different sources of molecular variation to assess the enrichment of Gene Ontology terms by combining *P* values from different association tests between sources and gene-level data. Here we integrated these proportions as different axes of molecular variation. We restricted the Gene Ontology terms to a minimum size of 20 genes and a maximum size of 1,000 genes as the default parameters of ActivePathways. To infer the pathways specifically altered in each archetype, we integrated the Pearson’s *P* value correlation of each gene from the expression matrix of 59,607 genes with the proportion from each archetype and we selected the pathways for which the enrichment source only corresponded to the tested archetype. We performed two kinds of analyses: one restricted to the genes positively correlated with the proportion (to obtain the upregulated pathways) and the other restricted to the negatively correlated genes (to identify the downregulated pathways).

### Reporting summary

Further information on research design is available in the Nature Portfolio Reporting Summary linked to this article.

## Online content

Any methods, additional references, Nature Portfolio reporting summaries, source data, extended data, supplementary information, acknowledgements, peer review information; details of author contributions and competing interests; and statements of data and code availability are available at 10.1038/s41588-023-01321-1.

## Supplementary information

Supplementary Information Supplementary Methods and Supplementary Figs. 1–24.

Reporting Summary

Peer Review File

Supplementary Tables Supplementary Tables 1–52.

## Data Availability

The genome sequencing, RNA-seq and methylation data have been deposited in the EGA database, which is hosted at the European Bioinformatics Institute and Centre for Genomic Regulation under accession number EGAS00001004812. Because raw omics datasets derived from humans are at risk of re-identification when combined with information from other public sources, access must be requested from the MESOMICS data access committee, as detailed at https://ega-archive.org/studies/EGAS00001004812. Minimum datasets of processed somatic alterations for genomic, transcriptomic and epigenomic data, sufficient to reproduce, interpret and extend our main results, are publicly available at https://github.com/IARCbioinfo/MESOMICS_data/tree/main/phenotypic_map/MESOMICS. A data note manuscript detailing all of the quality controls of the dataset is available at https://www.biorxiv.org/content/10.1101/2022.07.06.499003v1 (ref. ^82^). TCGA whole-exome sequencing, RNA-seq and methylation array data are available from the Genomic Data Commons portal (TCGA–MESO cohort^4^). Whole-exome sequencing and RNA-seq data from the Bueno and colleagues cohort^3^ are available from the EGA under accession number EGAS00001001563. Small variant lists, RNA-seq, expression array and methylation data for the Iorio and colleagues cohort^18^ are available from the Gene Expression Omnibus (accession number GSE29354), EGA (accession number EGAS00001000828) and Sequence Read Archive (accession number PRJNA523380). Corresponding drug responses are available from the cancerrxgene.org website (https://www.cancerrxgene.org/downloads/drug_data?tissue=MESO; accessed July 2021). Expression array data for the de Reyniès and colleagues cohort^5^ are available from the ArrayExpress platform (E-MTAB-1719) and corresponding drug response data are available from the supplementary material of Blum et al.^7^. All of the other data supporting the findings of this study are available within the article and its Supplementary Information files. Further information and requests for resources should be directed to and will be fulfilled by M.F. (follm@iarc.who.int). Source data are provided with this paper.

## Source data

Source Data Fig. 1 Statistical and figure source data.
Source Data Fig. 2 Statistical and figure source data.
Source Data Fig. 3 Statistical and figure source data.
Source Data Fig. 4 Statistical and figure source data.
Source Data Fig. 5 Statistical and figure source data.
Source Data Fig. 6 Statistical and figure source data.
Source Data Extended Data Fig. 1 Statistical and figure source data.
Source Data Extended Data Fig. 2 Statistical and figure source data.
Source Data Extended Data Fig. 3 Statistical and figure source data.
Source Data Extended Data Fig. 4 Statistical and figure source data.
Source Data Extended Data Fig. 5 Statistical and figure source data.
Source Data Extended Data Fig. 6 Statistical and figure source data.
Source Data Extended Data Fig. 7 Statistical and figure source data.
Source Data Extended Data Fig. 8 Statistical and figure source data.
Source Data Extended Data Fig. 9 Statistical and figure source data.
Source Data Extended Data Fig. 10 Statistical and figure source data.

## Code availability

All bioinformatics pipelines are available at https://github.com/IARCbioinfo (see Methods for details about which pipelines and versions were used for each analysis). A detailed R notebook allowing reproduction of the MOFA and Pareto tumor task inference results for the MESOMICS cohort is available at https://github.com/IARCbioinfo/MESOMICS_data.

## References

[1] Carbone M (2019). Mesothelioma: scientific clues for prevention, diagnosis, and therapy. CA Cancer J. Clin..

[2] WHO *Classification of Tumours, Thoracic Tumours* (5th edn) (International Agency for Research on Cancer, 2020).

[3] Bueno R (2016). Comprehensive genomic analysis of malignant pleural mesothelioma identifies recurrent mutations, gene fusions and splicing alterations. Nat. Genet..

[4] Hmeljak J (2018). Integrative molecular characterization of malignant pleural mesothelioma. Cancer Discov..

[5] De Reyniès A (2014). Molecular classification of malignant pleural mesothelioma: identification of a poor prognosis subgroup linked to the epithelial-to-mesenchymal transition. Clin. Cancer Res..

[6] Alcala N (2019). Redefining malignant pleural mesothelioma types as a continuum uncovers immune–vascular interactions. EBioMedicine.

[7] Blum Y (2019). Dissecting heterogeneity in malignant pleural mesothelioma through histo-molecular gradients for clinical applications. Nat. Commun..

[8] Nicholson AG (2020). EURACAN/IASLC proposals for updating the histologic classification of pleural mesothelioma: towards a more multidisciplinary approach. J. Thorac. Oncol..

[9] Fernandez-Cuesta L, Mangiante L, Alcala N, Foll M (2021). Challenges in lung and thoracic pathology: molecular advances in the classification of pleural mesotheliomas. Virchows Arch..

[10] Cortés-Ciriano I (2020). Comprehensive analysis of chromothripsis in 2,658 human cancers using whole-genome sequencing. Nat. Genet..

[11] ICGC/TCGA Pan-Cancer Analysis of Whole Genomes Consortium. (2020). Pan-cancer analysis of whole genomes. Nature.

[12] Quinton RJ (2021). Whole-genome doubling confers unique genetic vulnerabilities on tumour cells. Nature.

[13] Creaney J (2022). Comprehensive genomic and tumour immune profiling reveals potential therapeutic targets in malignant pleural mesothelioma. Genome Med..

[14] Argelaguet R (2020). MOFA+: a statistical framework for comprehensive integration of multi-modal single-cell data. Genome Biol..

[15] Courtiol P (2019). Deep learning-based classification of mesothelioma improves prediction of patient outcome. Nat. Med..

[16] Baylin SB, Jones PA (2016). Epigenetic determinants of cancer. Cold Spring Harb. Perspect. Biol..

[17] Sondka Z (2018). The COSMIC cancer gene census: describing genetic dysfunction across all human cancers. Nat. Rev. Cancer.

[18] Iorio F (2016). A landscape of pharmacogenomic interactions in cancer. Cell.

[19] Hausser J, Alon U (2020). Tumour heterogeneity and the evolutionary trade-offs of cancer. Nat. Rev. Cancer.

[20] Hausser J (2019). Tumor diversity and the trade-off between universal cancer tasks. Nat. Commun..

[21] Turini S, Bergandi L, Gazzano E, Prato M, Aldieri E (2019). Epithelial to mesenchymal transition in human mesothelial cells exposed to asbestos fibers: role of TGF-β as mediator of malignant mesothelioma development or metastasis via EMT event. Int. J. Mol. Sci..

[22] Shipony Z (2014). Dynamic and static maintenance of epigenetic memory in pluripotent and somatic cells. Nature.

[23] Chapel DB (2020). MTAP immunohistochemistry is an accurate and reproducible surrogate for CDKN2A fluorescence in situ hybridization in diagnosis of malignant pleural mesothelioma. Mod. Pathol..

[24] Alexandrov LB (2020). The repertoire of mutational signatures in human cancer. Nature.

[25] Steele CD (2022). Signatures of copy number alterations in human cancer. Nature.

[26] Bergstrom EN (2022). Mapping clustered mutations in cancer reveals APOBEC3 mutagenesis of ecDNA. Nature.

[27] Ladan MM, van Gent DC, Jager A (2021). Homologous recombination deficiency testing for BRCA-like tumors: the road to clinical validation. Cancers.

[28] Toh M, Ngeow J (2021). Homologous recombination deficiency: cancer predispositions and treatment implications. Oncologist.

[29] Ghafoor A (2021). Phase 2 study of olaparib in malignant mesothelioma and correlation of efficacy with germline or somatic mutations in *BAP1* gene. JTO Clin. Res Rep..

[30] Martínez-Jiménez F (2020). A compendium of mutational cancer driver genes. Nat. Rev. Cancer.

[31] De Rienzo A (2016). Gender-specific molecular and clinical features underlie malignant pleural mesothelioma. Cancer Res..

[32] Kato S (2016). Genomic landscape of malignant mesotheliomas. Mol. Cancer Ther..

[33] Shukuya T (2014). Identification of actionable mutations in malignant pleural mesothelioma. Lung Cancer.

[34] Mansfield AS (2019). Neoantigenic potential of complex chromosomal rearrangements in mesothelioma. J. Thorac. Oncol..

[35] McLoughlin KC, Kaufman AS, Schrump DS (2017). Targeting the epigenome in malignant pleural mesothelioma. Transl. Lung Cancer Res..

[36] Pastorino S (2018). A subset of mesotheliomas with improved survival occurring in carriers of *BAP1* and other germline mutations. J. Clin. Oncol..

[37] Hylebos M (2018). Molecular analysis of an asbestos-exposed Belgian family with a high prevalence of mesothelioma. Fam. Cancer.

[38] Bielski CM (2018). Genome doubling shapes the evolution and prognosis of advanced cancers. Nat. Genet..

[39] Turcan S (2012). *IDH1* mutation is sufficient to establish the glioma hypermethylator phenotype. Nature.

[40] Margueron R, Reinberg D (2011). The Polycomb complex PRC2 and its mark in life. Nature.

[41] Zauderer MG (2017). A randomized phase II trial of adjuvant galinpepimut-S, WT-1 analogue peptide vaccine, after multimodality therapy for patients with malignant pleural mesothelioma. Clin. Cancer Res..

[42] Phipps AI (2015). Association between molecular subtypes of colorectal cancer and patient survival. Gastroenterology.

[43] Malta TM (2018). Glioma CpG island methylator phenotype (G-CIMP): biological and clinical implications. Neuro. Oncol..

[44] Sreejit G (2014). The ESAT-6 protein of *Mycobacterium tuberculosis* interacts with beta-2-microglobulin (β2M) affecting antigen presentation function of macrophage. PLoS Pathog..

[45] Zanetti M (2017). Chromosomal chaos silences immune surveillance. Science.

[46] Gerstung M (2020). The evolutionary history of 2,658 cancers. Nature.

[47] Fujiwara T (2005). Cytokinesis failure generating tetraploids promotes tumorigenesis in p53-null cells. Nature.

[48] López S (2020). Interplay between whole-genome doubling and the accumulation of deleterious alterations in cancer evolution. Nat. Genet..

[49] Advani SM (2018). Clinical, pathological, and molecular characteristics of CpG island methylator phenotype in colorectal cancer: a systematic review and meta-analysis. Transl. Oncol..

[50] Noushmehr H (2010). Identification of a CpG island methylator phenotype that defines a distinct subgroup of glioma. Cancer Cell.

[51] Hughes LAE (2013). The CpG island methylator phenotype: what’s in a name?. Cancer Res..

[52] Moarii M, Reyal F, Vert J-P (2015). Integrative DNA methylation and gene expression analysis to assess the universality of the CpG island methylator phenotype. Hum. Genomics.

[53] Maley CC (2017). Classifying the evolutionary and ecological features of neoplasms. Nat. Rev. Cancer.

[54] Vendramin R, Litchfield K, Swanton C (2021). Cancer evolution: Darwin and beyond. EMBO J..

[55] Gould, S. J. & Eldredge, N. Punctuated equilibria: an alternative to phyletic gradualism. In Schopf, T.J.M. *Models in Paleobiology* 82–115 (Freeman Cooper, 1972).

[56] Zolondick AA (2021). Asbestos-induced chronic inflammation in malignant pleural mesothelioma and related therapeutic approaches—a narrative review. Precis. Cancer Med..

[57] Southwood TRE, May RM, Hassell MP, Conway GR (1974). Ecological strategies and population parameters. Am. Nat..

[58] Napolitano A (2016). Minimal asbestos exposure in germline *BAP1* heterozygous mice is associated with deregulated inflammatory response and increased risk of mesothelioma. Oncogene.

[59] Adashek JJ, Goloubev A, Kato S, Kurzrock R (2021). Missing the target in cancer therapy. Nat. Cancer.

[60] Gay CM (2021). Patterns of transcription factor programs and immune pathway activation define four major subtypes of SCLC with distinct therapeutic vulnerabilities. Cancer Cell.

[61] Dora D (2020). Neuroendocrine subtypes of small cell lung cancer differ in terms of immune microenvironment and checkpoint molecule distribution. Mol. Oncol..

[62] Owonikoko TK (2021). YAP1 expression in SCLC defines a distinct subtype with T-cell-inflamed phenotype. J. Thorac. Oncol..

[63] Galateau-Salle F, Churg A, Roggli V, Travis WD, World Health Organization Committee for Tumors of the Pleura. (2016). The 2015 World Health Organization Classification of Tumors of the Pleura: advances since the 2004 classification. J. Thorac. Oncol..

[64] WHO *Classification of Tumours of the Lung, Pleura, Thymus and Heart* (4th edn) (International Agency for Research on Cancer, 2015).

[65] Wasserstein RL, Lazar NA (2016). The ASA statement on *P*-values: context, process, and purpose. Am Stat..

[66] Alcala N (2019). Integrative and comparative genomic analyses identify clinically relevant pulmonary carcinoid groups and unveil the supra-carcinoids. Nat. Commun..

[67] Di Tommaso P (2017). Nextflow enables reproducible computational workflows. Nat. Biotechnol..

[68] Li H, Durbin R (2009). Fast and accurate short read alignment with Burrows–Wheeler transform. Bioinformatics.

[69] Faust GG, Hall IM (2014). SAMBLASTER: fast duplicate marking and structural variant read extraction. Bioinformatics.

[70] Tarasov A, Vilella AJ, Cuppen E, Nijman IJ, Prins P (2015). Sambamba: fast processing of NGS alignment formats. Bioinformatics.

[71] Van der Auwera, G. A. & O’Connor, B. D. *Genomics in the Cloud: Using Docker, GATK, and WDL in Terra* (O’Reilly Media, 2020).

[72] Benjamin, D. et al. Calling somatic SNVs and indels with Mutect2. Preprint at *bioRxiv*10.1101/861054 (2019).

[73] Kim S (2018). Strelka2: fast and accurate calling of germline and somatic variants. Nat. Methods.

[74] Wang K, Li M, Hakonarson H (2010). ANNOVAR: functional annotation of genetic variants from high-throughput sequencing data. Nucleic Acids Res..

[75] Cameron, D. L. et al. GRIDSS, PURPLE, LINX: Unscrambling the tumor genome via integrated analysis of structural variation and copy number. Preprint at *bioRxiv*10.1101/781013 (2019).

[76] Wala JA (2018). SvABA: genome-wide detection of structural variants and indels by local assembly. Genome Res..

[77] Chen X (2016). Manta: rapid detection of structural variants and indels for germline and cancer sequencing applications. Bioinformatics.

[78] Rausch T (2012). DELLY: structural variant discovery by integrated paired-end and split-read analysis. Bioinformatics.

[79] Jeffares DC (2017). Transient structural variations have strong effects on quantitative traits and reproductive isolation in fission yeast. Nat. Commun..

[80] Mose LE, Perou CM, Parker JS (2019). Improved indel detection in DNA and RNA via realignment with ABRA2.. Bioinformatics.

[81] Du P (2010). Comparison of Beta-value and M-value methods for quantifying methylation levels by microarray analysis. BMC Bioinformatics.

[82] Genova AD (2022). A molecular phenotypic map of malignant pleural mesothelioma.. Gigascience.

