# Supplementary material for: Multiomic analysis of malignant pleural mesothelioma identifies molecular axes and specialized tumor profiles driving intertumor heterogeneity
Source: Nat Genet. 2023 Mar 16;55(4):607–18. doi: 10.1038/s41588-023-01321-1 (PMC10101853; doi:10.1038/s41588-023-01321-1)
Supplement: Supplementary file 1 — Supplementary Methods and Supplementary Figs. 1–24. [file 41588_2023_1321_MOESM1_ESM.pdf]

nature genetics

Article

https://doi.org/10.1038/s41588-023-01321-1

Multiomic analysis of malignant pleural mesothelioma identifies molecular axes and specialized tumor profiles driving intertumor heterogeneity

In the format provided by the authors and unedited

Contents

1 Supplementary methods **3**
1.1 Clinical data description . . . 3
1.2 The MESOMICS cohort description . . . 3
1.3 Pathological review details . . . 3
1.4 Artificial Intelligence analysis details . . . 4
1.5 Immunohistochemistry . . . 4
1.6 Survival analysis quality control and replication . . . 4
1.7 DNA Sequencing . . . 5
1.7.1 Whole-Genome DNA Sequencing (WGS) details . . . 5
1.7.2 Variant calling and filtering on DNA details . . . 5
1.7.3 Microsatellite instability detection . . . 5
1.7.4 Copy number variant calling details . . . 6
1.7.5 Structural variant calling details . . . 6
1.7.6 Damaging variants and driver detection . . . 7
1.7.7 Comparison with PCAWG and TCGA . . . 7
1.7.8 Identification of complex mutational process in MPM tumors . . . 7
1.7.9 *TERT* promoter mutation analyses . . . 8
1.8 RNA Sequencing . . . 8
1.8.1 RNA Sequencing (RNA-seq) details . . . 8
1.8.2 Data processing details . . . 8
1.8.3 Quality controls . . . 9
1.8.4 Variant calling and filtering on RNA . . . 9
1.8.5 Fusion transcript analysis . . . 9
1.8.6 Alternative splicing analysis . . . 9
1.8.7 Processing of publicly available expression array data . . . 9
1.8.8 Immune contexture deconvolution from expression data . . . 9
1.8.9 Association between gene expression and frequent deletions . . . 10
1.8.10 WGD expression analyses . . . 10
1.9 DNA methylation . . . 10
1.9.1 EPIC 850k methylation array details . . . 10
1.9.2 Data processing details . . . 10
1.9.3 Processing of publicly available DNA methylation data . . . 11
1.9.4 Global methylation level . . . 11
1.9.5 Regional methylation analysis . . . 11
1.9.6 Deconvolution of enhancer methylation profile . . . 12
1.9.7 CpG island methylator phenotype index details . . . 12
1.9.8 Annotating IlluminaHumanMethylationEPIC array probes with gene ID . . . 12
1.9.9 Correlation between methylation and expression . . . 13
1.10 Epithelial-mesenchymal transition methylation quantification . . . 13
1.10.1 Epithelial-mesenchymal transition expression score . . . 13
1.10.2 Methylation . . . 13
1.11 Epithelial (E) and Sarcomatoid (S) scores . . . 13
1.12 Genomic instability scores . . . 13
1.13 Integrative unsupervised analyses . . . 14
1.13.1 Pre-processing of methylation data details . . . 14
1.13.2 Pre-processing of copy number changes details . . . 14
1.13.3 Pre-processing of drug response data . . . 14
1.13.4 Multi-omic integrative analyses details . . . 14
1.13.5 Interpreting MOFA latent factors . . . 15
1.13.6 Survival prediction . . . 17
1.13.7 Intra tumor heterogeneity analyses . . . 17
1.14 Evolutionary tumor trade-off analyses . . . 18
1.14.1 Pareto task identification details . . . 18
1.14.2 Interpretation of MPM polyhedron details . . . 18
1.15 Clonal reconstruction . . . 18

1.15.1 Small variants subclonal reconstruction . . . 18
1.15.2 CNV clonality reconstruction . . . 19
1.16 Inferring the timing of alterations . . . 19
1.16.1 Molecular time dating . . . 19
1.16.2 Chronological time dating . . . 20

2 Supplementary references **20**

3 List of Supplementary Tables **46**

List of Figures

S1 Multi-Omics Factor Analysis (MOFA) latent factors of the major MPM cohorts. . . . 22
S2 Reference-free deconvolution (MeDeCom) of MESOMICS enhancer DNA methylation data. . . . 23
S3 Association between survival and MOFA latent factors in the MESOMICS cohort. . . . 24
S4 Overview of the MPM cell lines dataset for integration with MOFA. . . . 25
S5 Comparison of the best Pareto front fit in (A) morphological factor (LF2) and adaptive-response factor (LF3) space and (B) ploidy factor (LF1) and CIMP factor (LF4) space. . . . 26
S6 Copy Number profile for major MPM cohorts. . . . 27
S7 Extrachromosomal DNA (ecDNA) predictions for MESOMICS cohort. . . . 28
S8 Example of co-occurrence of kataegis and ecDNA. . . . 29
S9 Chromothripsis analysis of MESOMICS cohort. . . . 30
S10 Chromothripsis, mRNA fusions, and clustered SV signature. . . . 31
S11 Overview of positive homologous recombination-deficiency (HRD) samples predicted by CHORD. . . . 32
S12 Homologous recombination-deficiency pattern in MPM cell lines and PARP inhibitor response. . . . 33
S13 Comparative analysis of tumor mutational burden. . . . 34
S14 MPM drivers. . . . 35
S15 Comparative analysis of mRNA fusions per tumor types and mRNA-fusion network for major MPM cohorts. 36
S16 Overview of *TERT* alterations in the MESOMICS samples. . . . 37
S17 Schematic representation of mesothelioma driver epigenetic regulatory gene (ERG) functions. . . . 38
S18 Pathways and top down-regulated genes of whole-genome doubling (WGD)+ samples. . . . 39
S19 Relationship between acinar phenotype and BAP1 protein expression measured by immunohistochemistry (IHC). . . . 40
S20 Alternative splicing in malignant pleural mesothelioma. . . . 41
S21 Timing of copy number gains in mutation time. . . . 42
S22 Patterns of intra-tumor heterogeneity in 13 samples. . . . 43
S23 Timing of small variants. . . . 44
S24 Comparison of the number of alterations in gene drivers between T/N matched samples and tumor-only samples of the epithelioid type (n=77 samples, 38 T-only and 39 T/N). . . . 45

1 Supplementary methods

1.1 Clinical data description

Detailed information from Santé Publique France (SPF), the French National Public Health Agency, about probability of exposure (no evidence found-0, possible-1/3, likely-2/3, and very likely-1), frequency (sporadic-0.25, intermittent-0.5, frequent-0.75, and persistent-1), intensity (low-1, intermediate-2, high-3, and very high-4), and duration of the asbestos exposure (in years) was available for 47 patients as the result of a supervised survey, the national program for pleural mesothelioma surveillance (Programme national de surveillance du mésothéliome pleural, PNSM)[83]. In order to compare exposure levels between patients and to reduce the number of variables, we computed a lifetime exposure score in units of years of persistent low-intensity asbestos exposure, by multiplying the probability, frequency, intensity, and duration of the exposure. This score is analogous to the pack-years concept used for tobacco smoking that also balances intense, short-duration with weaker, long-duration exposures[84]. Indeed, ten years of very likely, sporadic, very high intensity asbestos exposure leads to the same score ($10 \times 1 \times 0.25 \times 4 = 10$) as ten years of very likely, persistent, low-intensity exposure ($10 \times 1 \times 1 \times 1 = 10$) (**Table S2-3**). In order to improve the power of some of the statistical analyses, we constructed a categorical age variable where age was discretized into 3 classes (class1: (29, 63], class2: (63, 71], and class3: (71, 90], in years).

We tested the associations between clinical variables; in particular, between a batch variable (sample provider) and the main variable of interest (histopathological type or major epithelioid subtype) or important biological covariables such as sex, age, smoking status, and asbestos exposure, using Fisher's exact tests. We found that the sample provider was not significantly associated with the clinical variables (from **Table S2**), while sex was significantly associated with smoking status and asbestos exposure (Fisher's exact test q-value = 0.0002 and q-value = 0.03, respectively).

1.2 The MESOMICS cohort description

Age at diagnosis, sex, smoking status, asbestos exposure, treatment information (surgery, chemotherapy, radiotherapy, immunotherapy, and cancer history), and survival data (calculated in months from surgery to last day of follow up or death) were collected for all patients. Tumor stage was not available as this information was not collected in the French MESOBANK records. Note that this distribution of MPM types and epithelioid subtypes does not represent the true clinical distribution because of the bias we have introduced by including only samples with sufficient tumor content and good quality DNA and RNA. The tumor content estimated by our pathologist (F.G.-S.) in the series ranged from 10 to 100%. Similarly, the presence of infiltration was also evaluated in the H&E (hematoxylin and eosin) slides, and it ranged from 0% to 45% (**Table S2**). In addition, using the H&E slides, whole-image artificial intelligence analyses were undertaken to identify the most clinically relevant morphological features, and a score was calculated using the MesoNet algorithm (see details in section **Artificial Intelligence model details**)[15]. As expected, the median overall survival (OS) for the whole series was 14 months (IQR 12-17.1), with epithelioid (MME) showing the longest OS (15.8 months, IQR 13.9-24.5) followed by biphasic (MMB; 10.8 months, IQR 6.3-17.2) and sarcomatoid (MMS; 4.5 months, IQR 2.2-NA). The tubulopapillary subtype showed the best OS (ranging from 20.9 to 41.9 months), followed by trabecular (16.85-18.4), acinar (13.1-15.9) and solid (11.9-14.4), which showed the worst OS. In addition, the proportion of solid subtype was negatively associated with survival (q-values < 0.01) (**Table S13**). The ratio of men to women is 2.76, with no statistical association between sex and histological type or subtype. Of note, surgery but not radiotherapy were associated with a better prognosis (hazard ratios of 1.15 and -0.10, and $p = 0.033$ and 0.800, respectively; **Table S14**).

As shown in **Table S1**, the main difference between MESOMICS and the two previously published series of mesothelioma by Bueno[3] and the TCGA[4] was the percentage of MMS (12.5% in MESOMICS *vs*. 4% in both TCGA and Bueno). As expected, the median overall survival (OS) for the whole series was 14 months (very close to the TCGA and Bueno's series), with MME showing the longest OS (15.8 months) followed by MMB (10.8 months) and MMS (4.5 months). While the trend was similar to the TCGA and Bueno's series, the TCGA cohort showed longer survival for MME (24.1 months). Median age at diagnosis was 67.5 years (64 and 65.3 for TCGA and Bueno's series, respectively) and 73.3% of patients were male (84% and 82% in TCGA and Bueno's series, respectively). The ratio of men to women was 2.76, with no statistical association between sex and histological type or subtype.

1.3 Pathological review details

Tumor grade, immune infiltration, presence of necrosis and vessels were assessed for all 136 samples. In addition of histopathological types, we also assessed the epithelioid histopathological characteristics (architectural subtypes, cytological variants and stromal characteristics), which we subdivided into three subtypes, based on the recent IASCL-EURACAN

interdisciplinary meeting recommendations[8]: favorable prognosis (regrouping the acinar and papillary subtypes, and samples with abundant myxoid stroma), intermediate-prognosis (trabecular subtype), and unfavorable prognosis (solid subtype). Finally, we also assessed the sarcomatoid histopathological characteristics (simple, desmoplastic, low and high grade fusocelular, and with pleomorphic, heterologous, or transitional component) and both epithelioid and sarcomatoid histopathological characteristics in case of biphasic samples.

1.4 Artificial Intelligence analysis details

The model has been trained on a randomly selected training dataset of 2903 slides from the MESOPATH-NETMESO INCa network/MESOBANK (excluding samples from our MESOMICS cohort) and applied to the slides of our MESOMICS cohort. This model is trained to predict overall survival using only one H&E stained whole slide image per patient as input. It is therefore completely agnostic of any genomic information. The MesoNet model is composed of five elements. i) Matter extraction, which splits foreground and background images. ii) Tiling, which splits scanned images into tiles of 224 by 224 pixels. iii) Feature extraction, which uses the ResNet50 network, pre-trained in general image recognition tasks to obtain 2,048 features per tile. iv) Top and negative instances, which uses a 1-dimensional convolution layer to compute a score per tile, and picks the 10 highest and 10 lowest scores. v) Classification, using a multi-layer perceptron with two fully connected layers of 200 and 100 neurons and outputs a prediction score.

1.5 Immunohistochemistry

FFPE tissue sections (3µm thick) from 136 MPM samples were deparaffinized and stained with the Santa Cruz BAP1 antibody (cloneC-4, catalog number sc-28383) (dilution one to 50). Nuclear staining was considered positive (when nuclear expression was retained) or negative (complete loss of staining of all tumor cells with a positive internal control on the slides: fibroblast, lymphocytes and other non-tumor cells). Consequently, the positivity of BAP1 was reported as a score ranging from 0 complete loss of nuclear staining and 1 nuclear staining retained in 100% nuclei. Results are presented in **Table S2**.

1.6 Survival analysis quality control and replication

The proportional hazards hypothesis was checked using the Schoenfeld residuals (zph function). Univariate Cox analyses were performed for each important biological data such as sex, age, smoking status, and asbestos exposure as explanatory variable to evaluate their individual association with survival. In order to respect the minimal proportion of samples per group at 10%, we gathered current and former smoker groups together. Among the clinical data tested, only age and sex were both significantly associated with survival (Cox model p-value = 0.00021 and p-value = 0.045 respectively) (**Table S12**). As a result of univariate analyses, in order to assess survival associations with continuous molecular variables, we fitted Cox models by including sex and used the attained age scale, which provides a control for age effects without needing to fit an additional age parameter compatible with the proportional hazards assumption[85] (**Tables S13-20**).

For the replication of survival analyses (**Extended data fig. 4**), we used the MSK-IMPACT[38], Bueno[3], and TCGA[4] cohorts depending on the data available. For the ploidy factor, we used the Whole-Genome Doubling (WGD) status from the mesothelioma samples of the MSK-IMPACT cohort, using tumor location as an additional covariable (3 of the samples were from the peritoneum). As initially reported by the authors across more than 30 tumor types, we find that WGD is associated with poorer survival in the MSK-IMPACT MPM cohort. For the Morphology factor, we used the percentage epithelioid component as reported in the Bueno cohort by pathologists from microscopic examination of H&E slides, which is as expected associated with a better prognosis. For the Adaptive-response factor, we used an Adaptive *vs.* Innate immune response score, defined as the difference between the proportions of adaptive cells (B and T cells) minus the proportion of innate response cells (macrophages, monocytes, neutrophils), that we could obtain from the estimated immune cell proportions computed from the gene expression data in all RNA-seq cohorts using software quanTIseq (MESOMICS, Bueno, and TCGA). This is based on the fact that we found B and T cells are associated with good prognosis while macrophages are associated with bad prognosis, and this has been recently proven also true in mesothelioma (`https://doi.org/10.3389/fonc.2022.870352`). We found that this score was associated with a better prognosis. Finally, for the CIMP-index factor, we used a simpler CIMP-index proxy computed from only five genes, and replicated our results in the only cohort containing methylation data, the TCGA cohort. Following the original publication[38] , we fitted Cox models with age as a covariable. We used one-sided Wald tests with a cutoff p-value of 5% to assess the replication of the effects and their direction.

1.7 DNA Sequencing

1.7.1 Whole-Genome DNA Sequencing (WGS) details

After a complete quality control, genomic DNA (1µg) was used to prepare a library for whole genome sequencing, using the Illumina TruSeq DNA PCR-Free Library Preparation Kit (Illumina Inc., CA, USA, catalog number 20015963), according to the manufacturer's instructions. After quality control and normalization, qualified libraries were sequenced on a HiSeqX5 platform from Illumina (Illumina Inc., CA, USA), as paired-end 150 bp reads. Two lanes of HiSeqX5 flow cells were produced for each sample paired with matched-normal tissue or blood, in order to reach an average sequencing depth of 60x and one lane for the others in order to reach an average sequencing depth of 30x for the others. Sequence quality parameters were assessed throughout the sequencing run and standard bioinformatics analysis of sequencing data was based on the Illumina pipeline to generate FASTQ files for each sample.

1.7.2 Variant calling and filtering on DNA details

Our Nextflow workflow is based on the GATK best practices (https://github.com/IARCbioinfo/mutect-nf release v2.2b). We used a set of 79 blood samples (16 from the MESOMICS cohort and 73 from Gabriel *etal*.[86]) coming from the same sequencing machine from CNRGH in Paris as the panel of normal, and used GATK4's filtering module (FilterMutectCalls) with the recommended known variants VCF (gnomAD variants from GATK's Mutect2 bundle) and per sample estimates of the contamination rate obtained with GATK4's CalculateContamination (using the small panel of EXAC single-nucleotide polymorphisms (SNPs) from GATK4's Mutect2 bundle). Normalization of resulting variant calling format (VCF) files was performed with BCFtools version 1.10-2 as implemented in our workflow (https://github.com/IARCbioinfo/vcf_normalization-nf v1.1).

To call somatic variants on tumor-only samples (72/115) a similar procedure was performed (Mutect2 tumor-only mode) but included further germline filtering steps using a random forest (RF) classifier. A total of 20 features (gnomad, COSMIC, genomic location/impact, and features obtained directly from Mutect2) were selected to build a RF model to classify single nucleotide variants and small Indels as somatic or germline. For training the RF model a total of 46 tumors with matched normal mesothelioma whole-genome sequences were used. Variants on this subset were called using both the tumor-only and matched modes of Mutect2. The matched somatic calls (ground-truth) were used to split the variants of the tumor-only calls into germline and somatic classes and subsampled to mitigate bias arising from class imbalance during training (1:1 somatic:germline ratio, n =407,984). The dataset was divided into 75% for training (n =305,988) and 25% for testing (n =101,996), and the trained model reached an accuracy of 0.93 in the test set. A random forest model for single-nucleotide variants (SNVs) (rfvs01) was trained using a total of 326,388 (80%) variants (1:1 ratio). For indels, a random forest model (rfvi01) was built using a total of 337,442 variants (1:1 ratio, including 305,988 SVNs and 31,454 indels). To control the false positives (RF Model FDR=6.4%), given the highest expected proportion of germline variants in the prediction set, we set a cutoff (RF votes) of 0.5 and 0.75 for coding and non-coding variants, respectively. Finally, the RF models (rfvs01 and rfvi01) were used to classify a total of 1,454,942 variants (SNVs=1,317,200 and indels=137,742) of which 217,436 variants (including SNVs and indels) were classified as somatic. Importantly, the recurrent somatic SNVs are not enriched in tumor-only samples (Fisher's exact tests, **Fig. S24**). The point mutation calls for the MESOMICS cohort (n =448,434) include the matched calls (43 WGS) and the filtered tumor-only calls (72 WGS) (**Table S44**). The source code and the random forest models are available in the Github repository at https://github.com/IARCbioinfo/RF-mut-f.

We retrieved germline variants in a list of 26 genes involved in homologous recombination (*BRCA1*, *BRCA2*, *TP53BP1*, *ATM*, *ATR*, *ATRIP*, *BARD1*, *BLM*, *BRIP1*, *DMC1*, *MRE11A*, *NBN*, *PALB2*, *RAD50*, *RAD51*, *RAD51B*, *RAD51C*, *RAD51D*, *RIF1*, *RMI1*, *RMI2*, *RPA1*, *TOP3A*, *TOPBP1*, *XRCC2*, and *XRCC3*; from Toh and Ngeow The Oncologist 2021[28]) and classified as "Pathogenic" or "Likely pathogenic" in the ANNOVAR clinvar annotation (version 03.16.2020). We found such variants in 6 samples (1 germline mutation in *BRCA1*, 3 in *BRCA2*, 1 in *ATM*, and 1 in *NBN*); they were all at very low frequencies or absent from the 1000 genomes and exAc databases (frequency<0.0001). In addition, we note that the *BRCA1* and *BRCA2* germline-mutated tumors indeed were classified as such by CHORD, further validating our results. See results in **Table S46**.

1.7.3 Microsatellite instability detection

Microsatellite Instability (MSI) phenotypes were detected from cram files using msisensor-pro version 1.2.0 [87]. We scanned the reference genome GRCh38 for microsatellite information, and constructed a baseline for tumor-only samples using all normal samples from the MESOMICS cohort as a panel of normals. We then separately evaluated MSI in tumor-only samples using the "pro" module, and in tumor-normal pairs using the "msi" module. Tumor-only samples showed a slight offset of 2% in the percentage of microsatellites altered reported by the program but were otherwise comparable to tumor-normal pairs. A single sample had a high proportion of microsatellites altered (>8% in MESO_084) and was considered

microsatellite unstable. Results are reported in **Table S49**.

1.7.4 Copy number variant calling details

We benchmarked the PURPLE tumor-only mode by comparing the estimation of tumor purity, tumor ploidy, number of segments, percentage of genome changed (amplified, deleted), percentage of genome in neutral LOH (Loss Of Heterozygosity), and major/minor copy number alleles at gene level for matched and tumor-only PURPLE calls. CNV calls are reported in **Table S34** and presented in **Fig. 3b**, and compared to that from the Bueno[3] and TCGA[4] cohorts in **Fig. S6**. TCGA copy number data was downloaded from the TCGA portal (TCGA-MESO, https://portal.gdc.cancer.gov/, March 2021) corresponding to the allele-specific copy number segment data from genotyping arrays.

We observed a high concordance (pearson correlation) between tumor-only and matched PURPLE calls for tumor purity ($r > 0.98$), tumor ploidy ($r = 1$), number of cnv segments per tumor ($r > 0.98$), percentage of genome changed (amplified, deleted, $r > 0.99$), and percentage of genome in neutral LOH ($r > 0.99$). Moreover, the concordance between tumor-only and matched PURPLE calls was also high at gene level with major and minor copy number alleles reaching $R > 0.96$. However, we observed artifactual focal amplifications and deletions near telomeric and centromeric regions and short segments (<13999 bp) that were not called when using the matched data. These regions were identified and the segments overlapping these regions were removed from the tumor-only calls. In addition, slightly negative copy number estimates (in]-0.5,0[) were rounded to 0, and copy number estimates below -0.5 were excluded because they likely correspond to noisy read depth regions. The copy number calls for the MESOMICS cohort (115 WGS) include the matched PURPLE calls (43 WGS) and the filtered tumor-only PURPLE calls (72 WGS). Importantly, the recurrent somatic CNVs are not enriched in tumor-only samples except for one single gene and all q-values>0.05 after Benjamini-Hochberg adjustment (Fisher's exact tests, **Fig. S24C-D**, **Table S33-34**). Whole genome doubling samples were called in genomes with more than 10 autosomes with major allele copy number > 1.5. Near haploid samples were identified as those with LOH genome percentage larger than 80%. Finally, recurrent genomic regions of DNA copy-number alterations in the 115 WGS were identified with GISTIC2.0[88] (version 2.20.23, -conf 99%) using as input the PURPLE CNV calls (log2(totalcopynumber)-1) (**Table S34**). Cohort-level profiles (**Fig. 3b**) were computed and plotted using ACNViewer [89] version 2.2 (see singularity image at https://github.com/IARCbioinfo/acnviewer-singularity).

For replication of the analyses using the whole-exome sequencing data from the TCGA[4] and Bueno[3] cohorts (**Fig. S6**), as PURPLE is only suited for WGS data, we used software Facets[90] instead, as implemented in our pipeline (https://github.com/IARCbioinfo/facets-nf v. 2.0).

1.7.5 Structural variant calling details

For matched WGS, DELLY was run in somatic SV discovery mode using the hg38 blacklisted DELLY regions ("-x human.hg38.excl.tsv"; the list excludes centromere, telomere, and heterochromatin regions, as well as alt, decoy, and unknown contigs of hg38) and the tumor/normal WGS pairs. A list of somatic SVs passing all filters was generated using the DELLY somatic filter, which considers somatic SVs as those with at least 10 fold coverage in the tumor sample and without evidence of normal read support for the alternative allele (ALT support in normal equal 0). Manta was run in somatic SV discovery mode using the tumor/normal WGS (–normalBAM and –tumorBAM options) and excluding the non-chromosome contig sequences (alt and decoy) of hg38 (–callRegions option). The somatic SVs passing all Manta filters (minPassSomaticScore >=30) were considered for the consensus step. SvABA was run using our in-house Nextflow workflow (https://github.com/IARCbioinfo/svaba-nf, revision number 1.0) to identify somatic and germline SVs using the tumor/normal WGS. The somatic SVs passing all SvABA filters were considered for the consensus step. The overlap of filtered somatic SV calls was performed using SURVIVOR (merge subcommand) considering as matching SV breakpoints those at a maximum distance of 1kb (ignoring SV type and SV strand).

To filter germline SVs in tumor-only samples we trained a random forest model for each SV caller. The SV random forest model includes a total of 19 features, which are associated with external SV databases, custom panel-of-normal SVs, genomic regions, and SV features derived from SV callers. The training of the random forest model for each SV caller was performed using the matched WGS (57 including multi-region samples). First, the somatic calls from the matched WGS were used as the ground-truth during training and evaluation of each SV random forest model. Second, tumor-only calls were generated for the matched data using the tumor WGS for Manta and DELLY. For SvABA, the somatic and germline SVs called by the somatic mode were merged to generate the tumor-only calls from the matched data. Third, the panel of normals for each matched WGS and SV caller combinations were generated by integrating 45 germline SV calls (excluding the respective normal sample) with SURVIVOR (merge command). Fourth, a total of 12,454, 16,720, and 12,264 SVs at 1:1 somatic:germline proportions were used to train (75%) and evaluate (25%) the random forest models of DELLY, Manta, and SvABA, respectively. The accuracy achieved on the benchmark set was 0.9, 0.89 and 0.88 for DELLY, SvABA, and Manta SV RF models, respectively. Finally, the SV random forest models were used to filter the germline SVs from tumor-

only samples using a cutoff (RF votes) of 0.5 and 0.75 for coding and non-coding SVs, respectively. SVs matching one present in the custom PON or located in centromeric regions were discarded. SV call set for each tumor-only sample was created using the same steps performed for the matched WGS (merging DELLY, SVaba and Manta calls with SURVIVOR and keeping single caller predictions with read support ≥ 15). Moreover, SVs found in more than 4 samples in the tumor-only series were also classified as potentially germline and removed from the final consensus set. The SVs calls for the MESOMICS cohort (**Table S41**, n =12,914) include the matched SV calls (43 WGS, $n = 4,685$) and the filtered tumor-only SV calls (72 WGS, n =8,229). Importantly, the recurrent somatic SVs are not enriched in tumor-only samples (Fisher's exact tests, **Fig. S24B**, **Table S41**). The source code and the SV random forest models are available in the Github repository at https://github.com/IARCbioinfo/ssvht.

1.7.6 Damaging variants and driver detection

Mutational cancer driver genes have been detected using the state-of-the-art integrative oncogenomics pipeline (IntOGen[30]), that distinguishes signals of positive selection from neutral mutagenesis across a cohort of tumors by combining multiple driver detection methods. The IntOGen pipeline was run for each cohort separately, and also for the combined cohort to gain in statistical power, and to detect mutational driver genes that may be specific to each of them (**Fig. S14C** and **Fig. 4**, left panel). Of note, variants occurring on mitochondria chromosome chrM have not been considered in this analysis. Genes that drive tumorigenesis upon SVs have simply been selected based on their recurrence, using a cutoff of five samples (**Fig. S14A**).

The damaging SNVs, indels and structural variants have been selected as follows. First, for SNVs and small indels, we used ANNOVAR annotations to restrict the list to the exonic or splicing, non-synonymous variants. For multi-nucleotide polymorphisms (MNP), we used the Coding Change ANNOVAR procedure to infer the protein changes occurring and in case of any amino acid changes, we classified the event as damaging. Finally, we removed structural variants for which the breakpoints lead to harmless changes for the coding sequence of the gene such as large in frame deletion in a single intron. The oncoplot (**Fig. 4**) was plotted using R package maftools[91] version 2.10.05.

1.7.7 Comparison with PCAWG and TCGA

Tumor Mutational Burden comparison of Mesothelioma and TCGA cohorts (Fig. S13A) was performed with the maftools[91] package (v2.6.05). The PCAWG data for mRNA fusions (version 1.0), SVs (version consensus_1.6.161116), CNVs (consensus.20170119), and number of SNVs represented in **Fig. S13B** were downloaded from the PCAWG site (https://dcc.icgc.org/releases/PCAWG, accessed in June 2022), using the white- and gray-listed samples from the sample sheet v1.4 (2016-09-14). The copy number burden (CNB) was computed as the number of segments, thus reflecting the degree of fragmentation of the genome.

1.7.8 Identification of complex mutational process in MPM tumors

Mutational SBS signatures were de novo discovered and decomposed into COSMIC mutational signatures with the SigProfilerExtractor [24] tool, version 1.0.17. The SNVs called by both Mutect2 and Strelka (nextflow workflow https://github.com/IARCbioinfo/strelka2-nf v1.2a) on the T/N samples were used as input for SigProfilerExtractor to avoid caller specific signatures; note that we also chose to include the three non-chemonaive samples in order to check the presence of platinum-agent-associated mutational signatures. Five de novo signatures were identified and decomposed with high fidelity (cosine similarities greater than 0.93) into 10 known COSMIC signatures (see **Table S45**). Note we identified a small number of tumors (6) with the APOBEC signature SBS2, which tend to have a low-burden of age-related signatures (**Extended data fig. 7D**) and half of which also have the other APOBEC signature SBS13, as reported in some PCAWG cohorts[11] such as B-cell non-Hodgkin leukemia[24] (**Extended data fig. 7C**). Copy Number signatures were called using SigProfilerExtractor version 1.1.3 as described in Steele *etal*.[25] and using as input the PURPLE copy number segments with allele-specific copy numbers rounded to the nearest integer, removing samples with ambiguous integer copy numbers (i.e., those with integer total CN different from the sum of minor and major integer CN, due to the presence of multiple subclones with different CN states). We identified 4 de novo signatures that were decomposed with high fidelity (cosine similarities greater than 0.9) into 7 COSMIC signatures (v3.1, see signatures in **Table S35**), and processes associated with each signature were retrieved from Steele *etal*.[25]. SV signatures were also called using the SigProfiler framework (version 1.1). Finally, detection and classification of clustered mutations (kataegis analysis) was performed as described in Bergstrom *etal*.[26]. The list of clustered mutations per tumor including their classes are provided in **Table S42**, and represented in **Fig. S10**.

Chromothripsis regions were identified by combining SVs and CNV calls with the svpluscnv[92] R package version 0.9.1. To identify shattered regions' breakpoints from CNVs and SVs, breakpoints were counted by splitting the genome into 10Mb windows. Contiguous windows with a high density of breakpoints were merged into larger shattered regions. Then

interleaved SVs and variations in copy number state signatures were used to differentiate chromothripsis from focal events such as double minutes. Additionally, following recent practices[10], we classified the shattered region into high and low confidence by considering the number of oscillating CN segments: high-confidence calls were classified as those displaying an oscillation pattern between two copy number states in at least seven adjacent CN segments, others were classified as low-confidence calls (**Table S39**, **Fig. S9**).

Amplicon predictions were performed using the AmpliconArchitect program[93] version 1.2. In brief, the copy number variants were called using the CNVkit program (version 0.9.7), which is the recommended CNV caller to identify seed for AmpliconArchitect. Seed selection was performed following the recommended criteria (minimum segment length of 50Kb and minimum copy number gain of 4.5) using the amplified_intervals.py (amplified_intervals.py –gain 4.5 –cnsize_min 50000 –ref GRCh38) script provided by the AmpliconArchitect package. AmpliconArchitect was then run with default parameters using the selected seeds and the tumor CRAM files as input. In particular, AmpliconArchitect uses the UCSC cytoBands for the position for centromeres; although some of the segments reported overlap with some centromeric regions in chr13, we prefered to report these segments, because these are the default centromeric positions so likely used by a majority of AmpliconArchitect users, and because AmpliconArchitect accounts for repetitive and low-complexity regions and these amplicons were each supported by multiple split-reads in these samples (>25 breakpoints per ecDNA amplicon), which we believe is unlikely to be an artifact. Finally, the AmpliconClassifier program was run to classify the amplicons generated by AmpliconArchitect into ecDNA, BFS, Complex, linear or non-amplified classes (**Table S38**, **Fig. S7**). MESO_019 was the only tumor where a circular amplicon encompassing known oncogenes was found, so we plotted its the only non-trivial cycle found by AmpliconArchitect, using CycleViz v0.1.2 tool from the AmpliconArchitect authors (`https://github.com/jluebeck/CycleViz`; **Fig. 3c´**, middle panel). Finally, the homologous recombination deficiency samples were identified using the R package CHORD[94] version 2.0 (**Table S40**). Following CHORD recommendation, four HRD positive samples were marked with a not determined HRD type because they have less than 30 SV.

1.7.9 *TERT* promoter mutation analyses

Point mutations within the *TERT* promoter region (chr5: 1,294,956-1,295,406, hg38) were identified from the VCF file outputs of WGS prior to filtering T-only variants using the random forest filter (see **Variant calling and filtering on DNA**). Prefiltered VCF files were used due to low mappability of the region that results in high false negative point mutation detection rates. Genomic coordinates were selected specifically as all previously reported *TERT* promoter mutations in mesothelioma (C158A, A161C, C228T, C250T) are contained within the above region[95,96]. Three of four reported mutations were identified in seven samples: A161C (chr5: 1,295,046 in hg38 coordinates), C228T (chr5: 1,295,113), and C250T (chr5: 1,295,135). Results are presented in **Fig. S16**.

1.8 RNA Sequencing

1.8.1 RNA Sequencing (RNA-seq) details

We collected two technical replicates, MESO_051_TR and MESO_115_TR, from two different patients and coming from the same RNA extraction as MESO_051_T and MESO_115_T respectively but sequenced separately. Libraries were prepared using the Illumina TruSeq mRNA stranded sample preparation Kit (Illumina Inc., CA, USA, catalog number 20020595). Library preparation started with 1μg total RNA. After poly-A selection (using poly-T oligo-attached magnetic beads), mRNA was purified and fragmented using divalent cations under elevated temperature. The RNA fragments underwent reverse transcription using random primers. This is followed by second strand complementary DNA (cDNA) synthesis with DNA Polymerase I and RNase H. After end repair and A-tailing, indexing adapters were ligated. The products were then purified and amplified (14 PCR cycles) to create the final cDNA libraries. After library validation and quantification (Agilent 4200 Tapestation), equimolar amounts of the library were pooled. The pool was quantified by using the Peqlab KAPA Library Quantification Kit and the Applied Biosystems 7900HT Sequence Detection System. The pool was sequenced by using an Illumina Novaseq 6000 sequencing device and a paired end 100nt protocol.

1.8.2 Data processing details

Reads were trimmed for the adapter sequence using Trim Galore (version 0.6.5 for expression quantification, and version 0.4.2 for alternative splicing analyses), then mapped to reference genome GRCh38 (using annotation gencode version 33) with STAR software (version 2.7.3a). Then, reads were realigned locally using ABRA2[80] (workflow `https://github.com/IARCbioinfo/abra-nf` release v3.0), and base quality scores were recalibrated using GATK (workflow `https://github.com/IARCbioinfo/BQSR-nf` release v1.1). Expression was quantified for each sample, generating a raw read count table with gene-level quantification for each gene of the comprehensive gencode gene annotation file (release 33), as well as a table with Gene fragments per kilobase million (FPKM), using StringTie software (version 2.1.2) (Nextflow pipeline accessible at

https://github.com/IARCbioinfo/RNAseq-transcript-nf release v2.2). Quality control of the samples was performed at each step. FastQC software (version 0.11.9; https://www.bioinformatics.babraham.ac.uk/projects/fastqc/) was used to check raw reads quality and RSeQC software (version 3.0.1) was used to check alignment quality.

1.8.3 Quality controls

We performed dimensional reduction on expression data as quality control, using Principal Component Analysis (PCA) (function dudi.pca from R package ade4 version 1.7-16). PCA was performed on the variance-stabilized read counts of the 5,000 most variable genes for (i) (MESOMICS) 109, (ii) (Bueno[3]) 180, (TCGA[4]) 73, and (iii) (3-cohorts: MESOMICS, Bueno, and TCGA) 362 samples (**Table S27**). For each set, samples were plotted by their coordinates to visualize outliers. For each dataset, linear regression analysis was performed to determine any significant association between these PCs and technical variables such as RNA-seq batch, macrodissection and provider. We found no outliers, and no batch effect in this data.

1.8.4 Variant calling and filtering on RNA

We used Mutect2 with the –allele flag to force genotyping of variants identified by mutect in the whole-genome sequencing data to call variants on the 126 RNA sequencing data for validation (workflow https://github.com/IARCbioinfo/mutect2-nf release v2.2b with option –genotype).

1.8.5 Fusion transcript analysis

Fusion transcripts were detected using Arriba[97] for the MESOMICS, Bueno[3], and TCGA[4] cohorts. First, RNA-seq reads were aligned using STAR (2.7.6a) to the hg38 reference. Second, Arriba was used to call mRNA-fusions using the STAR alignment (BAM) and Arriba blacklisted regions (-b option). For the MESOMICS cohort, we additionally integrated the genomic SVs by including the SV breakpoints into the calling (-d option). Finally, high-quality mRNA-fusion predictions for all MPM cohorts were defined as those Arriba predictions classified as high confidence and with a minimum support of ten reads from paired-end and split-read alignments. The **Table S43** contains all the mRNA fusions passing the aforementioned filters, and **Fig. S15** represents recurrently altered genes.

1.8.6 Alternative splicing analysis

The raw read files from the 109 samples of the MESOMICS cohorts were trimmed using Trim Galore version 0.4.2 and pseudo aligned with Salmon version 1.8.0, using GRCh38 reference genome (using annotation gencode version 33) to build an index. Percent Spliced In (PSI) of the alternative splicing events of all genes were calculated from the Transcripts Per Million (TPMs) using SUPPA2[98]. Transcript usage was also calculated using SUPPA2. Principal Component Analysis (PCA) was used to cluster the samples based on their alternative splicing pattern profile, using PSIs from the top 6,366 splicing events, which explained 50% of the variance (**Fig. S20**). Spearman correlation tests were performed among the principal components and the MOFA Factors and Archetypes. Gene Ontology Cellular Component analysis was used to identify alternative splicing events contributing to PCs.

1.8.7 Processing of publicly available expression array data

Raw expression array CEL files from Iorio and colleagues[18] and de Reynies and colleagues[5] were downloaded from public repositories (GEO: GSE29354 and ArrayExpress: E-MTAB-1719, respectively) and processed using the RMA algorithm (justRMA function from the affy R package version 1.68.0). Annotations were downloaded from the hgu219.db and hgu133plus2.db packages (version 3.2.3).

1.8.8 Immune contexture deconvolution from expression data

The proportion of cells that belong to each of ten immune cell types (B cells, macrophages M1, macrophages M2, monocytes, neutrophils, NK cells, CD4+ T cells, CD8+ T cells, CD4+ regulatory T cells, and dendritic cells) were estimated from the RNA-seq data using software quanTIseq (downloaded 14 September 2020) using our workflow for parallel processing of samples (https://github.com/IARCbioinfo/quantiseq-nf release v1.1).

As technical validation of quantiseq results, we used EpiDISH R package (version 2.6.0) to estimate seven immune cell types (B cells, monocytes, neutrophils, NK cells, CD4+ T cells, CD8+ T cells, and eosinophils) as well as epithelial cells and fibroblasts from the DNA methylation data. The immune cell types for which the association with archetypes were the strongest (absolute Pearson's correlation coefficient $r > 0.4$, B cells, CD8+ T, and neutrophils) presented significant concordance between softwares (additionally to monocytes). The other estimates (NK cells and CD4+ T) have not been

confirmed in EpiDISH estimation, possibly because of the reference differences —such as the reference size, the number of cell types estimated— between softwares. Proportion of cells in the TCGA and Bueno samples were taken from the supplementary tables of Alcala *etal*.[6], which used the exact same software and version.

1.8.9 Association between gene expression and frequent deletions

Frequent copy number deletions have been defined from PURPLE calls and GISTIC2 analyses and presented in **Fig. 3a** and **b** (**Table S31, S32, S33, and S36**). The statistical association between frequent deletions described in **Fig. 3a** and gene expression has been tested using linear regression corrected with purity computed by PURPLE when available and with purity estimated by transcriptomic data when available or pathological estimated otherwise. Each copy number deletion type has been tested against true wild-type (WT) cases meaning with no other alterations (other copy number variant types, SNVs, SVs, or transcript fusion). The deleted cases can also include other alterations (**Fig. 3a**). Of note, we have reproduced our analyses and excluded these cases with other alteration types and found the same significant differences in gene expression with q-values$\leq$ 0.0001 for *NF2*, *MTAP*, and *BAP1*, and WT *vs*. heterozygous, and WT *vs*. homozygous for *CDKN2A* (not a significant difference between heterozygous and homozygous *CDKN2A* deletions).

1.8.10 WGD expression analyses

To identify significant differentially expressed genes associated with WGD status, we employed the same strategy introduced by Quinton *etal*.[12]. In brief, the expression of each gene (log2-transformed TPM+1 values) was modeled as a function of WGD + WGS-estimated purity + local_copy_Number. The purity, local_copy_number (log2(integer total copy number)), and WGD status were obtained from PURPLE predictions. Genes were considered significantly associated with WGD status if they had an FDR q-value of less than 0.05. Pathway enrichment analyses were performed with the hypeR[99] package (version 1.9.0) using a hypergeometric test with the MSigDB Hallmark gene sets (version 7.5.1) and the list of differentially expressed WGD genes and considering a universe of 23,467 genes (default in hypeR), and separating the list of upregulated and downregulated genes. Note that because the number of significantly upregulated and downregulated genes had different orders of magnitude (137 upregulated *vs*. 4,129), the proportion of overlap expected by chance are much higher in the downregulated GSEA while even modest overlaps (e.g., 6 genes) in the upregulated GSEA are sufficiently surprising to be statistically significant. Pathways with an FDR q-value of less than 0.05 were considered significantly associated with WGD status. We also tested an alternative model considering a multiplicative correction for tumor purity as suggested in Zheng *etal*. 2017[100], where expression is a function of WGD:purity + local_copy_Number:purity (where ":" indicates interaction); these results confirm the down-regulation of immune pathways and in particular interferon pathways in WGD+ tumors. Results are reported in **Tables S9-10** and presented in **Fig. S18**.

1.9 DNA methylation

1.9.1 EPIC 850k methylation array details

For each sample, 600 ng of purified DNA were bisulfite converted using the EZ-96 DNA Methylation kit (Zymo Research Corp., CA, USA, catalog number D5004) following the manufacturer's recommendations for Infinium assays. Then, 250 ng of bisulfite-converted DNA was used for amplification, fragmentation and finally hybridisation on Infinium MethylationEPIC v1.0 BeadChip (Illumina Inc., CA, USA, catalog number WG-317-1003), following the manufacturer's protocol. Chips were scanned using Illumina iScan to produce two-color raw data files (IDAT format).

Each chip holds eight samples, and the 140 samples were spread over 19 chips. We used stratified randomisation to mitigate the batch effects, samples were arranged over the chips to evenly distribute, in order of priority, histopathological type, major epithelioid subtype, provider, sex, smoking status, age and professional asbestos exposure. However, due to differences in the number of each histopathological type, and date of sample arrival, four of the 19 chips contained exclusively one type. Technical replicates were placed on different chips, whilst ITH and adjacent normal samples were placed on the same chip as their corresponding tumor sample. The position of samples on each chip was then randomized.

1.9.2 Data processing details

We first performed quality control checks on the raw data. Two-color intensity data of internal control probes were manually inspected to check the quality of successive sample preparation steps (bisulfite conversion, hybridisation, extension, and staining; function plotQC, ENmix). There was one outlier, the technical replicate MESO_056_T1, when comparing per sample log2 methylated and unmethylated chip-wise median signal intensity (function getQC, minfi), and no samples displayed an overall p-detection value > 0.01 (function detectionP, minfi). The poor quality sample, MESO_056_T1, was excluded from subsequent processing. Sex was assigned using a predictor based on the median total signal intensity of sex

chromosomes, with a cutoff of -2 log2 estimated copy number difference between males and females (function getSex, minfi). One sample was identified to be discordant between predicted (female) and clinically reported (male) sex, MESO_071_T. Whole genome sequencing results from matched blood confirmed that the participant was male, whilst the tumor displayed losses on chrY and gains on chrX.

Four groups of probes were removed: (i) poor performing probes with a p-detection value > 0.01 in at least one sample (16,497 probes discarded), p-detection value was computed by comparing the total signal (methylated and unmethylated) of each probe with the background signal level from non-negative control probes (function detectionP, minfi) (ii) cross-reactive probes (42,552 probes discarded), cross-reactive probes co-hybridise to multiple locations within the genome and therefore cannot be reliably investigated[101] (iii) probes on the sex chromosomes (17,144 probes discarded), and (iv) probes with SNPs within the single base extension site, or target CpG site, at a minor allele frequency of > 5% (database dbSNP build 137), (8,411 probes discarded, function dropLociWithSnps, minfi).

1.9.3 Processing of publicly available DNA methylation data

DNA methylation array data (IlluminaHumanMethylation450k BeadChip array IDAT files) from the TCGA mesothelioma cohort[4] were downloaded from the GDC legacy archive (https://portal.gdc.cancer.gov/legacy-archive/search/), and from the Iorio *etal*.[18] cell line cohort from GEO repository (dataset GSE68379), respectively. Datasets were then imported into R and pre-processed using R packages minfi (version 1.34.0) and ENmix (version 1.25.1) individually. Data processing was performed as per the MESOMICS cohort, no samples failed QC steps or were discordant for sex. Probes with p-detection value > 0.01, cross-reactive probes[102], probes on sex chromosomes, and those associated with SNPs were discarded. This resulted in normalized, filtered datasets of 439,417 probes for 74 samples for the TCGA cohort[4], and 436,125 probes for 21 samples for the Iorio cell line cohorts[18]. Beta and M-values were extracted (functions getBeta and getM, minfi), and probes recording M-values of $-\infty$ for at least one sample were replaced with the next lowest M-value in the dataset.

Where DNA methylation array data was required for the MESOMICS and TCGA cohorts together (see **Integrative unsupervised analysis**), data were combined and processed as follows. IDAT files for 126 MESOMICS samples (excluding ITH samples), and 74 TCGA samples were imported into R as separate RGSets. The TCGA RGSet was converted to array type IlluminaHumanMethylationEPIC and combined with the MESOMICS RGSet (function convertArray and combineArrays from R package minfi). All samples passed QC. One sample was identified to be discordant between predicted and clinically reported sex, MESO_071_T, as previously described. Subsequent processing was as per the MESOMICS cohort, and 56,308 probes were discarded (16,588 with p-detection value > 0.01, 26,254 cross-reactive, 8,838 sex chromosome, and 4,628 SNP-associated). This resulted in a normalized, filtered dataset of 396,145 probes for 200 samples. Beta and M-values were extracted (functions getBeta and getM, minfi), one hundred and twenty eight probes recorded M-values of $-\infty$ for at least one sample and were replaced with the next lowest M-value in the dataset. Seven samples were then removed from the beta and M matrices (all MESOMICS samples), three normal tissues, one technical replicate and three non-chimionaif samples, resulting in a dataset of 193 samples.

1.9.4 Global methylation level

DNA methylation level at *LINE1* repetitive elements was used as an estimate of global methylation level. Methylation levels at *LINE1* repetitive elements were calculated using the REMP package[103] (version 1.12.0) functions to extract M and beta values of CpGs that are located in *LINE1*. REMP functions were performed on the normalized, filtered M-table containing 781,245 probes, and identified 23,906 probes located in *LINE1* elements. Average M and beta values were then calculated for each individual sample across all *LINE1* probes respectively to obtain mean *LINE1* methylation levels per sample. The mean M-values were used for statistical analysis of associations between global methylation levels and features of interest (**Table S7**), while beta values were used for plotting significant findings. An examination of the mean methylation level across *LINE1* probes identified one outlier, MESO_040_T, for which the global level of methylation appears particularly low in comparison to the rest of the cohort, nevertheless this single sample only marginally influenced the relationship between *LINE1* and other variables mentioned in the main text.

1.9.5 Regional methylation analysis

Methylation profile within promoter, enhancer and gene body regions were examined as follows. Array probes were classified as promoter, enhancer, gene body or other, using annotations provided in the EPIC 850K array manifest b5 (version 1.0 b5, downloaded from: https://emea.support.illumina.com/downloads/infinium-methylationepic-v1-0-product-files.html). Probes with a value of "Promoter_Associated" in the column 'Regulatory_Feature_Group' were assigned as promoter probes, those with any value in the column 'Phantom5_Enhancers' were assigned as enhancer probes, and probes with a value including "Body" or "1stExon" in the column 'UCSC_RefGene_Group' were assigned as gene body probes.

Probes which fell into multiple groups were classified as promoter first, if applicable, then as enhancer probes. The dataset of 781,245 probes contained 102,341 promoter-assigned probes, 23,858 enhancer-assigned probes, and 317,281 gene body assigned probes.

Average M and beta values were calculated for each individual sample across all promoter, enhancer and gene body probes to obtain mean promoter, enhancer and gene body methylation levels per sample respectively. The mean M-values were used for statistical analysis of associations between regional methylation levels and features of interest, while beta values were used for plotting significant findings.

1.9.6 Deconvolution of enhancer methylation profile

Deconvolution of enhancer methylation levels was performed with non-negative matrix factorisation using R package MeDeCom[104] (version 1.0.0). The 5,000 most variable enhancer probes (variance calculated from beta values) were input to identify latent methylation components (LMC, cell-type specific methylation profiles). Values of $k = 3$ and $\lambda = 0.01$ were selected by examining the resulting cross-validation error plot; LMC values are reported in **Table S8**. Attributing the three resulting latent methylation components to cell types was performed through Pearson correlation tests of proportion of each LMC present in a sample against the proportion of individual cell types within each sample (**Fig. S2**). Proportions of B cells, M1 and M2 macrophages, monocytes, neutrophils, NK cells, T-CD8+, T-CD4+, T regulatory cells and dendritic cells were estimated from the result of quanTIseq analysis of RNA sequencing data (**Fig. S2A**), see **Immune contexture deconvolution from expression data**. Proportions of sarcomatoid and epithelioid cell types were estimated by histopathological review by the study pathologist F.G.-S., see **Pathological review** (**Table S2**).

1.9.7 CpG island methylator phenotype index details

We used probes denoted as "Island" in the Epic 850k array manifest b5 column 'Relation_to_UCSC_CpG_Island' and defined islands from manifest column 'Island_name.' CIMP index values ranged from 0.32 to 0.56, meaning 32% to 56% of all islands represented on the array were considered methylated per sample. The CIMP index in the MESOMICS cohort ranged from 0.32 to 0.47, and in the TCGA cohort[4] from 0.31 to 0.44. There was no significant difference in the distribution of CIMP index values between MESOMICS and TCGA samples (p-value = 0.98, student's t test).

We have been deliberate in not describing the finding of a higher CIMP index in some MESOMICS and TCGA samples as a CIMP+ phenotype as the method we have used to investigate CpG island methylation level, based on DNA methylation array data, differs from the classical gene panel model assessed through methylation-specific PCR [51,105]. Instead we refer to our measurement as a CIMP index, with a continuous rather than categorical interpretation. Nevertheless, we did compute a CIMP proxy based on the mean methylation level (beta value) of promoter CpG islands for the five genes in the Weisenberger *etal.*[105] panel. The CIMP proxy was computed based on the mean methylation level of promoter CpG islands for five genes only: *CACNA1G* (island coordinates (hg19): chr17:48636103-48639279), *IGF2* (chr11:2158951-2162484), *NEUROG1* (chr5:134870740-134872051), *RUNX3* (chr1:25255527-25259005), and *SOCS1* (chr16:11348541-11350803) (selected from Weisenberger *etal.*[105], **Table S7**). We found this proxy to be significantly correlated with CIMP index for both the MESOMICS (p-value= 1.36×10^{-36}, $r = 0.86$) and TCGA (p-value=3.08×10^{-24}, $r = 0.84$) cohorts (**Table S7**). Therefore we are confident in our finding of variation in CIMP within malignant pleural mesothelioma, and that a subset of samples display a high level of CIMP.

Another method for calculating CIMP index[7] was also tested (in the MESOMICS cohort only), here called CIMP-normal index. Probes located within CpG islands were retained, the mean beta value across all probes within each island was calculated for the three adjacent normal tissues available in the MESOMICS cohort. Islands whose methylation level was $< 30\%$ in all three adjacent normal samples were retained ($n = 15,824$), denoted as normally hypomethylated islands. The CIMP-normal index was then calculated as the proportion of these 15,824 islands with $\geq 30\%$ methylation (beta value ≥ 0.3) per sample. CIMP-normal index values ranged from 0.013 to 0.19, corresponding to 0.13% to 19% of normally hypomethylated islands to be hypermethylated per sample (**Table S7**). There was a significant correlation between the two CIMP index values calculated (p-value=3.27×10^{-66}, r =0.96). The method for CIMP-normal index was based on first identifying normally hypomethylated islands, therefore requiring normal pleura or mesothelium. The normal tissues available in the MESOMICS cohort are adjacent to mesothelioma samples, therefore that they are unlikely to be pure non-tumour tissues, as such, the CIMP index rather than the CIMP-normal index was used for subsequent analysis.

1.9.8 Annotating IlluminaHumanMethylationEPIC array probes with gene ID

Probes were assigned to a gene based on the contents of the EPIC 850K array manifest b5 column 'UCSC_RefGene_Name'. Additionally, promoter and enhancer only associated probes which did not have any gene annotation in the manifest column 'UCSC_RefGene_Name' were then assigned a 'nearest gene' annotation using the function matchGenes with the TxDb.Hsapiens.UCSC.hg19.knownGene library from R package bumphunter.

1.9.9 Correlation between methylation and expression

Correlation between methylation levels and gene expression was performed as follows. Regional level testing: probes were divided into promoter, enhancer and gene body (see **Regional methylation analysis**), probe groups were then filtered to retain only those with a difference of > 0.1 beta value between lowest and highest methylation level across 119 samples (samples input to MOFA analysis i). Pearson correlation tests were performed between the M-value of all probes within a region group and their corresponding gene expression level (normalized using variance stabilization transformation, filtered for genes having > 1 FPKM difference across 109 samples). This resulted in testing within 109 samples with both methylation and expression data of 37,067 promoter probes against expression of 8,444 genes, 20,308 enhancer probes against expression of 6,539 genes, and 262,820 gene body probes against expression 15,825 genes. p-values were adjusted for multiple testing using Benjamini-Hochberg method within region groups, probes were considered correlated with expression at q-value $\leq$ 0.05.

Island level testing: probes located within Cpg islands (denoted as "Island" in the Epic 850k array manifest b5 column 'Relation_to_UCSC_CpG_Island') were retained, the mean M-value across all probes within each island (identified from manifest column 'Island_name') was calculated per sample resulting in M-values for 24,891 CpG islands. Pearson correlation tests were performed between the M-value of each island and their corresponding gene expression level (normalized using variance stabilization transformation, filtered for genes having > 1 FPKM difference across 109 samples). Corresponding genes for each island were identified as the corresponding gene for each probe within the island (see **Annotating IlluminaHumanMethylationEPIC array probes with gene ID**). This resulted in testing within 109 samples with both methylation and expression data of 21,189 islands against expression of 12,992 genes.

1.10 Epithelial-mesenchymal transition methylation quantification

1.10.1 Epithelial-mesenchymal transition expression score

A score of epithelial-mesenchymal transition (EMT) per sample was calculated from variance-stabilized read counts as the mean expression of 52 mesenchymal-associated genes minus the mean expression of 25 epithelial-associated genes, as previously described[4,106]. A higher EMT score indicates a more mesenchymal-like gene expression profile than epithelial-like. Results are reported in **Table S7**.

1.10.2 Methylation

EMT gene methylation levels were calculated as follows. Firstly, all probes within promoter, enhancer, or gene body groups associated with at least one of the panel of 77 EMT-associated genes[106] in the manifest column 'UCSC_RefGene_Name' or 'nearest gene' annotation (function matchGenes, bumphunter) were selected. This resulted in 3,764 probes across all 77 genes, specifically 153 promoter probes corresponding to 17 EMT genes, 209 enhancer probes corresponding to 54 EMT genes, 2,575 body probes corresponding to 76 EMT genes, and an additional 827 probes to promoter, enhancer, or gene body regions, corresponding to 73 genes. The mean M and beta values across all epithelial and mesenchymal genes separately for each region group were then calculated per sample.

1.11 Epithelial (E) and Sarcomatoid (S) scores

For each sample, E- and S-scores were computed for the MESOMICS, TCGA[4], Bueno[3] and cell-lines samples[5,18] using expression data (normalized read count for MESOMICS, TCGA, Bueno and expression array data for cell-lines) and the method WISP from Blum *etal*.[7] (R package WISP version 2.3). The method relies en unsupervised clustering to identify three clusters, enriched for samples of the Epithelioid, Sarcomatoid histopathological types and Normal cells, respectively, and then uses these samples to produce signature expression profiles that are used to perform a deconvolution of all the samples using a constrained linear model. Results are presented in **Table S7** and **S23**.

1.12 Genomic instability scores

We estimated genomic instability from all omic layers: genomic, expression, and methylation profiles. From the genome, we calculated the proportion of changes in the genome in terms of copy number. From expression data, we computed a hallmark score using hallmarks of cancer[107] by summing the normalized read count of the genes belonging to each hallmark. Finally, we used global methylation level (see **Global methylation level** section) as a third proxy of genomic instability. Values are reported in **Table S7**. We performed pairwise comparisons between these three estimates and found significant correlations (q-value = 1.53×10^{-5}; q-value = 6.63×10^{-3}; and q-values = 1.39×10^{-3} for genomic *vs*. transcriptomic, genomic *vs*. epigenomic, and transcriptomic *vs*. epigenomic estimates, respectively).

1.13 Integrative unsupervised analyses

1.13.1 Pre-processing of methylation data details

We used M-values of 781,245, 426,213, and 396,145 CpGs from MESOMICS, TCGA[4], combined MESOMICS/TCGA, and Iorio cell line[18] cohorts cohorts respectively, which theoretically range from $-\infty$ to $+\infty$ and have a bimodal distribution, being not affected by heteroscedasticity contrary to beta values[81]. Following the same approach as for expression data, sex-chromosomes CpGs have been excluded (see **Methylation section**), resulting in 781,245, 426,213, 396,145, and 436,125 CpGs available after QC (see **DNA methylation Sequencing section**). After splitting the data into three sets (MethPro, MethEnh, and MethBod), we obtained respectively 37,884, 23,169 and 291,877 CpGs for analysis (i); 27,235, 4,953 and 125,228 for analysis (iii); 37,951, 5,174, and 132,546 for analysis (iv), and 30,387, 4,757, and 111,774 for analysis (v). To note, analysis (i) was performed with data from the EPIC 850K array, whilst analyses (iii)-(iv) were performed on data generated from either the EPIC aray or HM450K array. Therefore only probes found on both arrays were used for analyses (iii)-(iv). For comparison in analysis (i) based on EPIC array data, 77% of promoter probes (3,849), 78% of gene body probes (3,878), and 30% of enhancer probes (1,520) used are also on the HM450K array.

1.13.2 Pre-processing of copy number changes details

For the TCGA samples, the copy number state has been aligned on the hg19 genome. Hence, specifically for analysis (iv), the transformation of hg19 coordinates into hg38 has been required to integrate copy number data from both MESOMICS and TCGA samples within the same data set. To do so, we used liftOver R package (version 1.14.0) to transform segment coordinates into hg38 genome. Hg19 positions not found by the software because overlapping uncertain regions such as centromeres, have been replaced by the corresponding hg38 centromere coordinates. Then, for the remaining positions not found in the hg38 genome, we first listed, for each segment, the overlapping genes in hg19 and hg38 coordinates and compared the two lists. Then, we saved the same coordinates in case of identical lists and expanded the coordinates to include the overlapping genes that are missing. This expansion has been made only and only if the resulting segment length did not exceed an increase of 5% of the original segment and less than the maximum length difference observed in the transformation process made by liftOver. If these criteria were not filled, the given gene was not included and thus, the coordinates remained unchanged.

The copy number of each segment was computed as total = purity $\times$ total + (1-purity) $\times$ 2 and minor = purity $\times$ minor + (1-purity) with total and minor the value assigned for each gene in the Total and Minor data set, respectively. In case of segment breaks occurring within a given gene sequence, the mean value of the two segments overlapping is assigned to the gene. In the particular case of the MPM cell lines cohort, the copy number changes have been assessed using SNP arrays, giving only a round estimate of the copy number at the gene level and because the purity is estimated as 1, the resulting total data corresponds to integer values. Note that although available (see **Fig. S6**), because they were computed from exome data instead of genome-wide data as the MESOMICS and TCGA[4] cohorts, CNVs from the Bueno cohort were not included in MOFA.

1.13.3 Pre-processing of drug response data

We used drug response data (used IC50 in units of mean µM) only for the analysis (v) on MPM cell lines, combining the drug response of 265 drugs from Iorio[18]. Among them, three have also been tested on the de Reyniès cell lines[5] and their responses are reported in Blum *etal*[7].

1.13.4 Multi-omic integrative analyses details

Some samples did not have all the data sets chosen to be integrated available, such as for Bueno and colleagues' samples[3] missing methylation array data. Fortunately, MOFA was shown to handle missing data, including samples with entire 'omic techniques missing, by using the correlated signals from several datasets to accurately reconstruct latent factors[108]. Note that our sample size is in line with the size of n =100 that was shown to allow to capture the main sources of variation in simulated datasets in the original MOFA study, across a wide range of omic datasets (1 to 21), features per dataset (100 to 10,000), latent factors (5 to 60), and missing values (from 0 to 90%)[108]; we also mention that $n > 100$ fits general recommendations for dimensionality reduction based on matrix factorization such as PCA for stable latent factors and weights so the results from the sample can accurately be generalized to the population[109].

To compare multi-omic with uni-omic unsupervised analyses, we correlated the MOFA coordinates of the samples shared by MOFA and the PCAs with their coordinates in PCA-exp (see **RNA Sequencing**). Results show that the main 4 MOFA factors all have a counterpart in the PCA. For the MOFA-Cell lines, weights of the features from the drug layer and their correlations with the latent factors are represented in **Fig. S4** and **Tables S25-26**.

MOFA has multiple advantages when capturing molecular variation compared to classical clustering approaches such as ConsensusCluster+ and icluster+[110,111], such as allowing us to integrate arbitrarily many 'omic datasets (vs a single dataset in ConsensusCluster+ and four in icluster+), capturing both continuous and discrete independent sources of biological variation (*vs.* discrete clusters only), and quantifying the importance of each 'omic layer in separating samples and clusters (*vs.* unknown relationships between clusters). However, in order to compare MOFA results with previous results we compared our results with those obtained using the exact methods used in previous large-scale genomic studies of MPM (ConsensusCluster+ as in Bueno *etal.* 2016[3], and icluster+ as in Hmeljak *etal.* 2018[4]).

We ran consensus clustering on gene expression data using the ConsensusCluster+ R package version 1.60. Because contrary to MOFA, ConsensusCluster+ and icluster+ cannot handle missing data, from the original gene expression matrix input into MOFA of size 120x5,000, we remove samples that miss any of the 5,000 genes, resulting in a filtered gene expression matrix of 109 samples and 5,000 genes. We run consensus clustering on this gene expression matrix using ConsensusClusterPlus for $K = 2$ to 16, where K is the number of clusters. The clustering algorithm used is k-means. A consensus matrix heatmap was generated by the method for each K, as well as the consensus cumulative distribution function and the relative change in area under CDF curve as a function of K. We visualized samples in the space of each pair of MOFA latent factors from LF1 to LF4, and colored the samples by the results of consensus clustering with different K's. For each K and each latent factor, we quantitatively assess the association between the latent factor and the clusters using Kruskal-Wallis rank sum test and report the p-value of the test to investigate its statistical significance. Of note, we did not use the one-way ANOVA test, because the ANOVA assumptions - the homogeneity of variances across groups and the normality of data - were not met. To assess the clustering performance of consensus clustering, we used the Silhouette method. We use one minus the consensus value as the distance between two samples [112]. Based on this distance measure, the Silhouette value for each sample was computed to measure the similarity of a sample to its own cluster compared to other clusters. We visualized the average Silhouette width of all samples for K from 2 to 16. Using a range of selected values from -0.25 to 0.75 as thresholds, we also computed the proportion of samples with Silhouette width below the threshold for each K. This proportion of samples that were poorly clustered indicates the unstability of the clustering method. For $K = 3$, we show in a heatmap the co-clustering frequency patterns of the samples for all clustering methods considered in the consensus clustering.

We ran integrative clustering on MESOMICS data using the iClusterPlus R package version 1.32. The method takes only up to four matrices, so we used the following four matrices: one total CNV matrix, one alterations matrix, one gene expression matrix, and one matrix formed by concatenating the three methylation matrices input into MOFA. The CNV matrix had 5,000 features, the alterations matrix has 533, the gene expression matrix had 5,000, and the concatenated methylation matrix had 15,000. There were 105 samples left after the removal of those with missing data. We ran integrative clustering for $K = 2$ to 16 using the default number of points to sample. The alterations data is modeled by the bionomial distribution, while the others are modeled by Gaussian distributions. Similar to how we compared MOFA with the consensus clustering, we visualized samples in each two-dimensional latent factor space colored by the results of integrative clustering. For selected K, we also visualized samples in the one-dimensional space of one latent factor using the beeswarm plot. We performed the Kruskal-Wallis rank sum test between each latent factor and each K and report the test significance in a heatmap. We also assessed the clustering performance of integrative clustering. We retrieved the optimal latent space representation of the samples for each K from the integrative clustering method. We used the pairwise Euclidean distances between samples based on the latent space representation and the clustering result to compute the Silhouette value, as well as the proportion of samples with Silhouette width below the threshold. For $K = 4$, we show in a heatmap the co-clustering frequency patterns of the samples for clustering with all lambda parameters considered in the integrative clustering.

Results of the comparisons between ConsensusCluster+ and icluster+ and MOFA are shown in **Extended data fig. 10**. These methods capture some of the variation in the dataset: the three archetypes (**B** and **F**, to be compared with **Fig. 2a**), higher-ploidy samples (**G**, middle panel), and the CIMP index (**C**, **G**, right panels), but in a much cruder way than with MOFA and the Pareto task analysis, mostly because the discreteness assumption of clustering methods misses the inherent continuity in the data (noted in mesothelioma by Blum *etal.* 2019[7], and ourselves, Alcala *etal.* 2019[6]). Indeed we observe small and fragile clusters, with negative or low (<0.25) silhouette widths for a large number of samples (more than 25% of samples for K greater than or equal to 4, the value used in both Bueno *etal.* 2016[3] and Hmeljak *etal.* 2018[4]), indicating uncertain or wrong cluster assignment (**D** and **H**), and preventing downstream analyses. We think that these new analyses both validate our results, and at the same time highlight the need to use more modern techniques that were not yet available in previous mesothelioma studies. Note that MOFA is increasingly becoming a new standard in multi-omic analyses [113,114].

1.13.5 Interpreting MOFA latent factors

We selected latent factors for downstream analysis based on their prognostic value and the amount of molecular variance they explained. We first retained factors that were significantly associated with survival (Cox proportional hazards models with each LF as continuous variable, see **Survival analysis** section above, and **Table S15**). Then, we computed the variance

that each factor explained in each of the four types of alterations input into MOFA (genomic alterations, CNVs, gene expression, methylation) as in a classical principal component analysis: the sum across features of the squared cosine similarities between a focal latent factor and each feature, which is equal to the R^2 of a linear model with a alteration single as explained variable (e.g., the expression of gene X) and a latent factor as explanatory variable (i.e., expression_X Factor1), averaged across all alterations. Thus, for each of the seven 'omic datasets input into MOFA, and for each of the 10 LFs, we obtain a quantity that ranges from 0 (the latent factor explains no inter-patient variation for any feature of this dataset) to 100% (the latent factor explains all inter-patient variation for all features). We also show in **Extended data fig. 2** two extreme examples that illustrate a molecular feature which is badly explained by a latent factor (panel **A**) and a feature which is well-explained by a latent factor (panel **B**), and typical features associated with each latent factor (panels **D-G**). Note that each R^2 is thus computed independently, so the fact that we split methylation datasets into three does not impact the R^2 of the expression or alteration datasets for instance, and one could analyze in the future how each latent factor explains inter-patient variation with other choices of features (e.g., splitting expression into multiple pathways) without these new analyses affecting what the numbers we report in **Fig. 1a** and **Extended data fig. 2**. Also note that due to the difficulty to properly account for the proportion of variance explained for an indicator variable (0:wild type, 1:altered) using cosine similarities (or correlation coefficients or R^2 of linear models), we rather display in **Fig. 1a** the pseudo R^2 of McKelvey and Zavoina, using the Veall-Zimmermann estimator[115]. Indeed, classical R^2 for indicator variables are known to have a theoretical upper bound strictly smaller than 1 and thus to vastly underestimate the actual proportion of variance explained; in contrast, the Veall-Zimmermann, which is based on a logistic regression model which better models indicator variables, was shown to have no such upper bound and to have a similar interpretation as classical R^2 (proportion of variance in an indicator variable explained by a feature). We indeed show in **Extended data fig. 2C** that classical R^2 are much smaller than the Veall-Zimmermann pseudo R^2.

We retained factors explaining more than 10% of variance of at least one omic dataset and ordered factors based on the sum of the proportion of variance across all omic types they explained. This led to four latent factors for the discovery cohort. The resulting ten latent factors capture 54% of the molecular variability in the omic dataset integrated, while the first four latent factors alone capture 32% of the variance (**Table S5**). Additionally, because the likelihood of a latent factor within matrix factorization analyses such as MOFA and PCA does not depend on its sign, the directions of latent factors are arbitrary and we chose the direction that best fitted their interpretation: for LF1, from haploid (negative values) to tetraploid (positive values), for LF2, from low epithelioid component (negative values) to high epithelioid component (positive values), for LF3, from low infiltration (negative values) to high infiltration (positive values), and for LF4 from low CIMP index (negative values) to high CIMP index (positive values). Note that the four LFs that have been retained for further analyses are statistically independent of one another. Indeed, we expect under statistical independence of the factors that the R^2 of a model combining all factors of the form feature Factor1+Factor2+Factor3+Factor4 (see "LF1 to LF4 predicted" in **Extended data fig. 2B**) would be the sum of the R^2 of each independent model feature Factor1, feature Factor2, feature Factor3, and feature Factor4. In contrast, if the factors were completely collinear, the R^2 of all independent models would be equal and the R^2 of the combined model would be equal to that of any of the independent models. We do observe that the R^2 of the combined model is very close to that expected under perfect statistical independence (see "LF1 to LF4 expected" and "LF1 to LF4 observed" in **Extended data fig. 2B**), corroborating the statistical independence of the factors for each omic dataset. Of note, some of the other latent factors are not totally statistically independent (LF5 and LF9). With larger cohorts, future studies might reproduce some of these other factors and have more power to detect their influence on survival and thus decide to study them to further stratify patients and we thus report them in the supplementary tables.

We tested the association between each LF and clinical, morphological, and epidemiological variables using linear regression (**Table S6**). As quality control, we also assessed their associations with the technical variables selected to detect potential batch effects in the data using linear regression (**Fig. S3D**). The proportion of cells that belong to different immune cell types (see **Immune contexture deconvolution from expression data** section) were quantified and correlated with each dimension using Pearson correlation tests (**Tables S2 and S6**), as were the association between MOFA latent factors and previous classifications (**Fig. 1b**).

In order to replicate our findings in Bueno and TCGA cohorts[3,4] we used MOFA analyses (ii) and (iii) and matched their resulting latent factors using those from MOFA-3-cohorts (MOFA analysis (iv) including the MESOMICS, Bueno, and TCGA samples) as intermediary between the discovery cohort (MOFA MESOMICS analysis (i)) and these two intedentant replication cohorts. We firstly performed Pearson's correlation tests between the position of Bueno and TCGA samples within the MOFA-3-cohorts and their position in the individual MOFA-TCGA and MOFA-Bueno (Fig. S1A). In the case of the TCGA cohort, we observed ambiguity in the identification of the Morphological factor (MESOMICS LF2) and Adaptive-response factor (MESOMICS LF3) in MOFA TCGA (**Fig. S1A**, middle panel) using simply this method for LFs identification. In order to improve the way we identify these factors within the TCGA data, we used the optimal transformation (rotation, translation, and scaling) between two point patterns according to the least-squares estimation from Umeyama[116]. We firstly transformed the MOFA-3-cohorts LFs to match those of the MOFA-MESOMICS, and then transformed the MOFA-TCGA LFs to match those of the transformed MOFA-3-cohorts. This resulted in the optimal match between the LFs of the MOFA-

MESOMICS and MOFA-TCGA cohorts, while preserving the relative position of samples within these LFs. In **Table S4**, we reported both the raw and transformed factors for MOFA-TCGA and all the replication analyses related to MOFA factors, in the TCGA cohort, have thus been done using the transformed MOFA-TCGA factors. Of note, in this process, we excluded the Ploidy factor from the set of factors used to transform the matrices because it would add noisiness to the computation since the samples without genomic data are very often imprecisely placed by the MOFA algorithm along the Ploidy factors.

1.13.6 Survival prediction

In order to evaluate the ability of the MOFA factors to predict survival, we compared twenty-two survival models: (i) a model based on the three histopathological types (categorical variables, MME, MMB, and MMS); (ii) a model based on the proportion of sarcomatoid content (continuous variable, fitted with a penalized cubic spline); (iii) a model based on the log2 ratio of CLDN15/VIM (C/V) expression in Bueno and colleagues[3] (continuous variable, fitted with a penalized cubic spline; see values in **Table S7**); (iv-vi) a model based on the E-score, S-score and the combination of the two, respectively, from Blum and colleagues[7] (continuous variable, fitted with penalized cubic splines without interaction; see values in **Table S7**); (vii) a model based on AI prognostic score (continuous variable, fitted with a penalized cubic spline); (viii-xi) models based on the a-dimensional summary of molecular data using either LF1, LF2, LF3, or LF4 as a continuous variable, respectively (each with a single continuous variable, fitted with a penalized cubic spline); (xii-xxii), models based on the two, three, four-dimensional summary of molecular data using both the combination of two, three, and four of the four LFs as continuous variables, respectively (continuous variables, fitted with penalized cubic spline without interaction). To do so, we assessed their fits using the time-dependent Area Under the ROC Curve (AUC) and its integral (iAUC[117], R package survAUC, version 1.1–1, **Tables S17-S18**), computed using the test set. This time-dependent AUC is used to evaluate the ability of an explanatory variable to predict patients with a survival lower or higher than a given threshold. Its integral summarizes the results of time-dependent AUC over the threshold value, providing an interpretation similar to that of classical AUC. In each model, we included sex and age; penalized splines were fitted using the pspline function from package survival, with three degrees of freedom. Because of the high proportion of missing asbestos and smoking status information and the absence of significant association between these variables and survival in univariate models (see **Survival analysis section**), smoking and asbestos were not included in the model as covariables.

To assess the out-of-sample prediction performance, we used 4-fold cross-validation in the MESOMICS cohort (**Extended data fig. 5A-C**, **Table S19**). We also assessed the prediction performance on a completely independent cohort by fitting the model on the whole MESOMICS cohort and testing it on the TCGA cohort[4], using bootstrapping (n =2,000 bootstraps) on the test set to assess variation in performance (**Extended data fig. 5D-F**, **Table S20**). Standard errors in the iAUC mean estimate were computed either from the 4 folds or the 2000 bootstraps, respectively, for the MESOMICS and TCGA. We also looked at the model fits on the MESOMICS cohort (**Extended data fig. 5A-C** and **Table S17**), which confirmed that the MOFA with 4 LFs (xxii) provided the best fit of all models, and also led to the lowest Akaike Information Criteria (AIC=524.5 for the model with the LFs, *vs.* AIC=569.8 for the best AIC of non-MOFA models, that of the S-score + E-score; **Table S17**, see **Table S16** for E- and S-scores in the TCGA and Bueno cohorts[3,4]), which shows that the greater number of parameters in the MOFA survival model is not enough to explain its better performance.

We also assessed the significance of surgery on overall survival after accounting for the four latent factors and sex, and found that surgery had a significant impact ($p = 0.007$, hazard ratio of 2.02, **Table S14**).

1.13.7 Intra tumor heterogeneity analyses

The ploidy, morphology, and CIMP factors represented in Extended data **Fig. 9** were identified in the MOFA-ITH by correlating the coordinates of non-ITH samples in the MOFA-ITH with their coordinates in the MOFA from Fig. 1, and choosing the largest match (correlations were all $r > 0.9$). To avoid spurious ITH to be detected, we excluded ITH samples with uncertain estimated ploidy (min and max ploidy estimates with a difference greater than 1), and because the ploidy factor overwhelmingly represents variance in genomic data (CNVs; **Table S52**), samples with missing WGD information were not represented in the ploidy factor (NA values in **Table S51**). Similarly, the Pareto front was fitted on the MOFA-ITH using the method described below (**Table S51**). Euclidean distances between each pair of samples were then computed for each factor of the MOFA-ITH separately (**Extended data fig. 9A** and **Fig. S22A**). Proportions of the tumor from different components (% Sarcomatoid, % Acinar, % immune infiltration) presented in **Extended data fig. 9B** are that reported by the pathologist, and included the constraint that % Sarcomatoid + % Epithelioid + % infiltration=100%, and % Acinar $\leq$ % Epithelioid (see data in **Table S50**).

1.14 Evolutionary tumor trade-off analyses

1.14.1 Pareto task identification details

In our case, three molecular spaces have been tested: LF2-LF3, LF2-LF3-LF4, and LF2-LF3-LF4-LF1. Each polyhedron fit is assessed by the ratio of the volume of the best-fitting polyhedron to the volume of the convex hull of the data (t-ratio). The more the data follows the Pareto optimality theory, the more the t-ratio metric approaches 1. Finally, the algorithm re-calculates the t-ratio on 1000 shuffles, keeping the distribution of loading on each axis but not the associations between them and computes a one-sided p-value to estimate the statistical significance of the fit.

We chose to represent the most significant fit with the smallest number k because of the limited number of samples but all the fit results are presented in **Table S28**, and the best fit and an alternative non-significant fit are presented in **Fig. S5** for illustration. Using MOFA axes, we found $k = 3$ archetypes in the LF2-LF3 space and reproduced, for each analysis (i), (ii), (iii), and (iv), the fit using the corresponding expression PCA in the PC1-PC2 space (see **Extended data fig. 3** for the fit for model ii, and iii). In order to evaluate the reproduction of the three archetypes discovered in (i) (MESOMICS cohort) into (ii) (Bueno's cohorts[3]) and (iii) (TCGA cohort[4]), we used (iv) (3-cohorts) and correlated the pairwise distance between archetypes and samples within each molecular map (**Table S28**). Overall, we found a strong concordance between the three analyses (minimum absolute Pearson's r =0.68; median r =0.84).

1.14.2 Interpretation of MPM polyhedron details

In all the analyses, the proportion of enriched genes within the enriched pathways ranged from 0.10 to 0.75 (**Table S30**). Used as a quality control of the enrichment results, we assessed the fold change between the 10% closest samples *vs*. the 10% furthest samples from each archetype of the enriched genes belonging to each enriched pathway. More specifically, in order to assign universal cancer task to each archetype, we referred to Hausser *etal.*[20] and examined the GO term descriptions to gather pathways in hyper-pathways as reported in **Table S30**. Of note, we focused our interpretation on the non-shared hyper-pathways to infer the specific cancer task of each phenotype. Also note that, because we are interested in reconstructing a genotype-to-phenotype map, we actually want to find genes which amplification or deletion directly impacts gene expression and thus contrary to the WGD- *vs*. WGD+ differential expression analyses, we purposely avoided correcting for copy number changes, which would have removed the signal and thus potentially lost the interesting biological impact of copy number changes on specialization.

Similarly, we tested the association between each archetype and genomic events using linear regression and more specifically, in order to infer genomic event effect-size on the Pareto front, we calculated the vector linking the centroids of the altered and wild-type groups (centroids function from sda R package v.1.3.8). To infer to what extent alterations drive the tumor cells toward specialization, we followed the method from Hausser *etal.*[20] and calculated the alignment of vectors with the front (angle between the Pareto front and the vector built from the altered and wild-type groups within the 4-factors space), after having normalized each LF (centering and division by standard deviation). Finally, we evaluated the driving role of genomic events associated to at least one archetype (**Table S48**) using these two variables (vector size and angle to Pareto front) by permutation tests (with 1,000 permutations) in which we randomized, one genomic event at a time, the altered and wild-type groups and compared this distribution from shuffled values with the observed values (**Extended data fig. 8**).

1.15 Clonal reconstruction

1.15.1 Small variants subclonal reconstruction

To obtain accurate estimates of variant allelic fractions (VAFs), we restricted the model fitting to somatic alterations with high-confidence VAF estimations (read depth greater than or equal to 60X and VAF greater than or equal to 0.05), and focused on samples with matched normal tissue or blood ($n = 43$). We only selected variants in high-confidence CN calls: regions with confident calls, excluding centromeric regions (based on UCSC annotation) that have notoriously more difficult CNV calling due to larger variance in reads mapping.

We inferred whether small somatic variants were clonal or subclonal using R package MOBSTER version 1.00. MOBSTER uses a mixture model to identify different clones in the VAF distribution. Importantly, the model uses evolutionary theory predictions to perform more accurate subclonal reconstructions, and can test whether subclones are under natural selection or neutral evolution by testing the presence of a "neutral tail" component, a Pareto type I distribution that is expected to be present in exponentially growing tumors evolving under neutral evolution[118]. For each sample, we first fit a mixture model to the VAF distribution from variants in regions with the most frequent CN (major and minor CN of 1 for most samples, major and minor CN of 2 and 1 or 3 and 1 for WGD samples, and 1 and 0 or 2 and 0 for GNH samples). For each sample, we compared the fit of models with or without neutral tail and with 1 to 3 clusters, with 10 repetitions per model with

different initializations, resulting in 6×10 models per sample; we chose the best model using the ICL statistic. We assessed the robustness of the fit using parametric bootstrapping; only models that were correctly inferred in more than 80% of the simulations were used. Of note, the clonal cluster also provides an estimate of the sample purity based on the VAF distribution. Multiple clusters denoting the presence of a subclone were identified in 13 samples. All 13 samples presented a single subclone, and all were in the low-adaptive response factor range (close to archetype 3), as expected from the high purity required to detect subclonal alterations (see **Table S2 and S50**); 3 samples presented a neutral tail, while the 10 others presented a selected subclone (**Fig. S23B**).

We finally assigned mutations that were not included in the model fit (small variants in regions with another CN, subclonal CNVs) to clones and subclones using their VAF and the fitted model. For each of the 13 samples where a subclone was identified, we recovered the cutoff cancer cell fraction (CCF) separating clonal and subclonal alterations according to the selected MOBSTER model. We then converted this threshold CCF to a threshold VAF by taking into account the CN state of each alteration using the formula $VAF_{thres} = CCF_{thres}\phi/[CN_{normal} \times (1-\phi) + CN_{total} \times \phi]$, where CN_{normal} is 2 for autosomal regions and 1 for sex chromosomes, CN_{total} is the total CN of the tumor, and ϕ is the MOBSTER-estimated purity. Variants were then assigned to the clonal and subclonal categories depending on which side of the threshold they fell. Note that this approach is similar to that used in the DPClust software[119], but using the recent evolutionary-theory aware probability distributions from MOBSTER instead of Dirichlet distributions. The proportion of clonal and subclonal alterations in the 13 samples where this analysis was possible are reported in **Table S2** and **Fig. S23C**. Note that the small number of samples with such clusters of subclonal alterations detected allowed us to further check visually the consistency between the fits of different CN regions (e.g. CN neutral LOH regions should have two clonal modes, one corresponding to variants in 1 or 2 copies, while diploid regions should have only clonal mode). Results for alterations in the driver list from **Fig. 4** are presented in **Fig. S23C**.

1.15.2 CNV clonality reconstruction

Clonality of CNVs was assessed using the estimated fractional copy number from PURPLE. Indeed, the PURPLE algorithm uses a penalized estimation of CN so that clonal CN segments are expected to have CN values close to an integer while subclonal segments have non-integer CN values; we thus classified as subclonal segments with a CN deviating from an integer value (fractional part between 0.2 and 0.8). Because of the difficulty of inferring the clonality of CNVs, we also assessed the clonality of CNVs using software Facets[90] (`https://github.com/IARCbioinfo/facets-nf`; release 2.0); only CNVs consistently called as clonal or subclonal by PURPLE and Facets are reported in **Fig. S21D** (see **Table S37** for all CNV clonalities). Although CNVs are generally called more accurately than small variants in T-only samples, for consistency with the rest of the clonality and evolutionary analyses, we restricted the analyses to tumors with a matched normal (n =43).

1.16 Inferring the timing of alterations

Due to the low tumor mutational burden in MPM and the high immune infiltration of many samples, we restricted the analysis to samples with large scale events (WGD or more than 10% of the genome with LOH), and to samples with matched normal tissue or blood (n =6).

1.16.1 Molecular time dating

Similarly to the approach from Gerstung and colleagues[46] implemented in package MutationTimeR, copy number gains and copy neutral losses of heterozygosity (LOH) were dated by comparing the number of alterations that were present in a single copy (that appeared after the event), N_r, to the ones that were present in multiple copies (that appeared before the event) N_l (**Fig. S21**). We computed Bayesian credibility intervals (BCI) for the timing of each gain and compared results with parametric bootstrapping confidence intervals (CI). BCI were obtained by assuming that N_r followed a Poisson distribution of parameters λ_r and λ_l, and uniform prior distributions over the interval [0,104] with the constraints that $\lambda_r < \lambda_l$, because the mutation rate λ_l includes both mutations that occurred before and after the copy number gain, while λ_r is limited to mutations that occurred before the gain; posterior distributions were numerically computed using a discrete grid approximation of size 1001, and used to compute the posterior distribution of timings t_e. Bootstrapping CI proceeded as in Gerstung and colleagues[46], first drawing 1000 N_{ri} values from a Poisson distribution of parameter N_r, and finally inferring t_e from the simulations. We show in **Fig. S21B** that both approaches provide very similar results (correlation between the center of the CI and BCI across dated events is r =0.99, p =4.8×10^{-14}), but the Bayesian estimates have the advantage of ensuring that $0 < t_e < 1$, because of the prior imposing that $\lambda_r < \lambda_l$, so they are the ones reported in the main text.

Synchronicity of duplications in the WGD sample was assessed by checking the overlap between CI for gains in the different segments considered.

1.16.2 Chronological time dating

We used the method validated by Gerstung and colleagues[46] to date amplification events that first estimated the temporal accumulation of CpG to TpG mutations ([C>T]pG), mostly due to spontaneous deaminations that would accumulate at an approximately constant rate through time. In order to check whether the small number of alterations present in some segments led to biases in our estimates, we performed the same analysis but using all mutations instead of just [C>T]pG mutations. Results showed no significant systematic bias (linear regression coefficient estimated at 0.85, with 95%CI [0.69,1.01]; **Fig. S21A**), and CI of [C>T]pG and CI of all mutations overlapped except for MESO_008, a non-chemonaive tumor that showed an excess of chemotherapy-associated mutational signatures that likely influenced the proportion of signatures associated with aging relative to other signatures. Overall, our results show that using all mutations increases the precision of estimates but does not bias the results as long as we exclude non-chemonaive samples, probably because MPM do not have SBS signatures of exogenous sources but rather only a slow temporal accumulation of mutations (see **mutational signatures** section), so results in the main text correspond to results for all mutations. Finally, we checked whether mutation accumulation showed a sign of temporal acceleration by comparing the number of small variants corrected for the effective genome size (defined as in Gerstung *etal.*[46] as $1/\text{mean}(m_i/C_i)$, with m_i the number of copies of alteration i and C_i the total CN at this position) with the age at diagnosis (**Fig. S23A**); the analysis showed that small variants fit a linear accumulation model, thus we used a rate of $\times 1$ for chronological dating.

Because of the limited number of samples with chronological dating, we focused on testing whether the timing of such events differed from that reported in other tumor types rather than the precise estimation of the timing in MPM. It was reported By Gerstung and colleagues[46] that whole-genome duplication (WGD), large-scale losses of heterozygosity, and amplification of the chromosomal region including *TERT*, typically predate diagnosis by a decade. We tested this hypothesis using outlier tests, where the p-value corresponds to the quantile of the empirical distribution of event timings from the PCAWG cohort[11] including more than 2500 tumors from 38 types. Note that outlier tests are designed to test whether values observed in mesothelioma were outliers in the empirical distribution of timings of such events in other cancers, but they cannot say whether there is a more subtle trend of slightly higher or lower average timing in mesothelioma. We report the results of the test of this hypothesis in **Fig. 5d**.

2 Supplementary references

83. Gilg Soit Ilg, A. *et al.* Programme national de surveillance du mésothéliome pleural (PNSM) : vingt années de surveillance des cas, de leurs expositions et de leur reconnaissance médico-sociale (1998-2017). *Archives des Maladies Professionnelles et de l'Environnement* **81**, 672 (2020).

84. Schaeffner, E. S., Miller, D. P., Wain, J. C. & Christiani, D. C. Use of an asbestos exposure score and the presence of pleural and parenchymal abnormalities in a lung cancer case series. *Int. J. Occup. Environ. Health* **7**, 14–18 (2001).

85. Griffin, B. A., Anderson, G. L., Shih, R. A. & Whitsel, E. A. Use of alternative time scales in Cox proportional hazard models: implications for time-varying environmental exposures. *Stat. Med.* **31**, 3320–3327 (2012).

86. Gabriel, A. A. G. *et al.* Genetic analysis of lung cancer reveals novel susceptibility loci and germline impact on somatic mutation burden. *bioRxiv* (2021) doi:10.1101/2021.04.26.21254132.

87. Jia, P. *et al.* MSIsensor-pro: Fast, Accurate, and Matched-normal-sample-free Detection of Microsatellite Instability. *Genomics Proteomics Bioinformatics* **18**, 65–71 (2020).

88. Mermel, C. H. *et al.* GISTIC2.0 facilitates sensitive and confident localization of the targets of focal somatic copy-number alteration in human cancers. *Genome Biol.* **12**, R41 (2011).

89. Renault, V. *et al.* aCNViewer: Comprehensive genome-wide visualization of absolute copy number and copy neutral variations. *PLoS One* **12**, e0189334 (2017).

90. Shen, R. & Seshan, V. E. FACETS: allele-specific copy number and clonal heterogeneity analysis tool for high-throughput DNA sequencing. *Nucleic Acids Res.* **44**, e131 (2016).

91. Mayakonda, A., Lin, D.-C., Assenov, Y., Plass, C. & Koeffler, H. P. Maftools: efficient and comprehensive analysis of somatic variants in cancer. *Genome Res.* **28**, 1747–1756 (2018).

92. Lopez, G., Egolf, L. E., Giorgi, F. M., Diskin, S. J. & Margolin, A. A. svpluscnv: analysis and visualization of complex structural variation data. *Bioinformatics* **37**, 1912–1914 (2021).

93. Deshpande, V. *et al.* Exploring the landscape of focal amplifications in cancer using AmpliconArchitect. *Nat. Commun.* **10**, 392 (2019).

94. Nguyen, L., W M Martens, J., Van Hoeck, A. & Cuppen, E. Pan-cancer landscape of homologous recombination deficiency. *Nat. Commun.* **11**, 5584 (2020).

95. Pirker, C. *et al.* Telomerase Reverse Transcriptase Promoter Mutations Identify a Genomically Defined and Highly Aggressive Human Pleural Mesothelioma Subgroup. *Clin. Cancer Res.* **26**, 3819–3830 (2020).

96. Quetel, L. *et al.* Genetic alterations of malignant pleural mesothelioma: association with tumor heterogeneity and overall survival. *Mol. Oncol.* **14**, 1207–1223 (2020).
97. Uhrig, S. *et al.* Accurate and efficient detection of gene fusions from RNA sequencing data. *Genome Res.* **31**, 448–460 (2021).
98. Trincado, J. L. *et al.* SUPPA2: fast, accurate, and uncertainty-aware differential splicing analysis across multiple conditions. *Genome Biol.* **19**, 1–11 (2018).
99. Federico, A. & Monti, S. hypeR: an R package for geneset enrichment workflows. *Bioinformatics* **36**, 1307–1308 (2020).
100. Zheng, X., Zhang, N., Wu, H.-J. & Wu, H. Estimating and accounting for tumor purity in the analysis of DNA methylation data from cancer studies. *Genome Biol.* **18**, 17 (2017).
101. Pidsley, R. *et al.* Critical evaluation of the Illumina MethylationEPIC BeadChip microarray for whole-genome DNA methylation profiling. *Genome Biol.* **17**, 208 (2016).
102. Chen, Y.-A. *et al.* Discovery of cross-reactive probes and polymorphic CpGs in the Illumina Infinium HumanMethylation450 microarray. *Epigenetics* **8**, 203–209 (2013).
103. Zheng, Y. *et al.* Prediction of genome-wide DNA methylation in repetitive elements. *Nucleic Acids Res.* **45**, 8697–8711 (2017).
104. Lutsik, P. *et al.* MeDeCom: discovery and quantification of latent components of heterogeneous methylomes. *Genome Biol.* **18**, 55 (2017).
105. Weisenberger, D. J. *et al.* CpG island methylator phenotype underlies sporadic microsatellite instability and is tightly associated with BRAF mutation in colorectal cancer. *Nat. Genet.* **38**, 787–793 (2006).
106. Mak, M. P. *et al.* A Patient-Derived, Pan-Cancer EMT Signature Identifies Global Molecular Alterations and Immune Target Enrichment Following Epithelial-to-Mesenchymal Transition. *Clin. Cancer Res.* **22**, 609–620 (2016).
107. Kiefer, J. *et al.* Abstract 3589: A systematic approach toward gene annotation of the hallmarks of cancer. in *Bioinformatics and Systems Biology* (American Association for Cancer Research, 2017). doi:10.1158/1538-7445.am2017-3589.
108. Argelaguet, R. *et al.* Multi-Omics Factor Analysis-a framework for unsupervised integration of multi-omics data sets. *Mol. Syst. Biol.* **14**, e8124 (2018).
109. Saccenti, E. & Timmerman, M. E. Approaches to Sample Size Determination for Multivariate Data: Applications to PCA and PLS-DA of Omics Data. *J. Proteome Res.* **15**, 2379–2393 (2016).
110. Wilkerson, M. D. & Hayes, D. N. ConsensusClusterPlus: a class discovery tool with confidence assessments and item tracking. *Bioinformatics* **26**, 1572–1573 (2010).
111. Mo, Q. *et al.* Pattern discovery and cancer gene identification in integrated cancer genomic data. *Proc. Natl. Acad. Sci. U. S. A.* **110**, 4245–4250 (2013).
112. Monti, S., Tamayo, P., Mesirov, J. & Golub, T. Consensus Clustering: A Resampling-Based Method for Class Discovery and Visualization of Gene Expression Microarray Data. *Mach. Learn.* **52**, 91–118 (2003).
113. Argelaguet, R., Cuomo, A. S. E., Stegle, O. & Marioni, J. C. Computational principles and challenges in single-cell data integration. *Nat. Biotechnol.* **39**, 1202–1215 (2021).
114. Efremova, M. & Teichmann, S. A. Computational methods for single-cell omics across modalities. *Nat. Methods* **17**, 14–17 (2020).
115. Veall, M. R. & Zimmermann, K. F. Evaluating Pseudo-R2's for binary probit models. *Quality & Quantity* vol. 28 151–164 Preprint at https://doi.org/10.1007/bf01102759 (1994).
116. Umeyama, S. Least-squares estimation of transformation parameters between two point patterns. *IEEE Transactions on Pattern Analysis and Machine Intelligence* vol. 13 376–380 Preprint at https://doi.org/10.1109/34.88573 (1991).
117. Chambless, L. E. & Diao, G. Estimation of time-dependent area under the ROC curve for long-term risk prediction. *Stat. Med.* **25**, 3474–3486 (2006).
118. Williams, M. J., Werner, B., Barnes, C. P., Graham, T. A. & Sottoriva, A. Identification of neutral tumor evolution across cancer types. *Nat. Genet.* **48**, 238–244 (2016).
119. Nik-Zainal, S. *et al.* The life history of 21 breast cancers. *Cell* **149**, 994–1007 (2012).

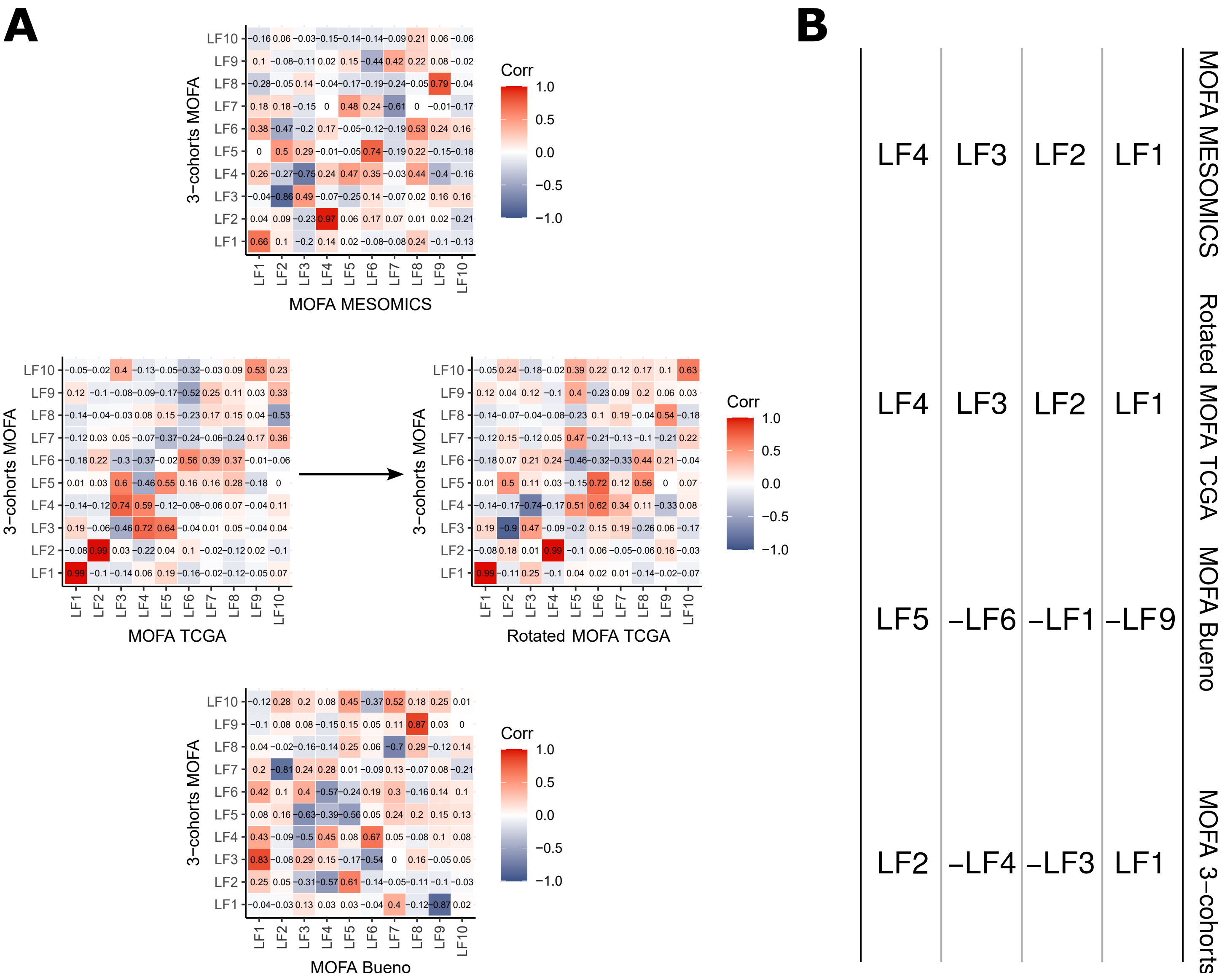

Figure S1. Multi-Omics Factor Analysis (MOFA) latent factors of the major MPM cohorts. A) Correlation between MOFA Latent Factors (LF) integrating the 3-cohorts and individuals cohorts. B) Matching for LF1, LF2, LF3, and LF4 (columns) in the different MOFA results across MPM cohorts. Corr: Correlation coefficient.

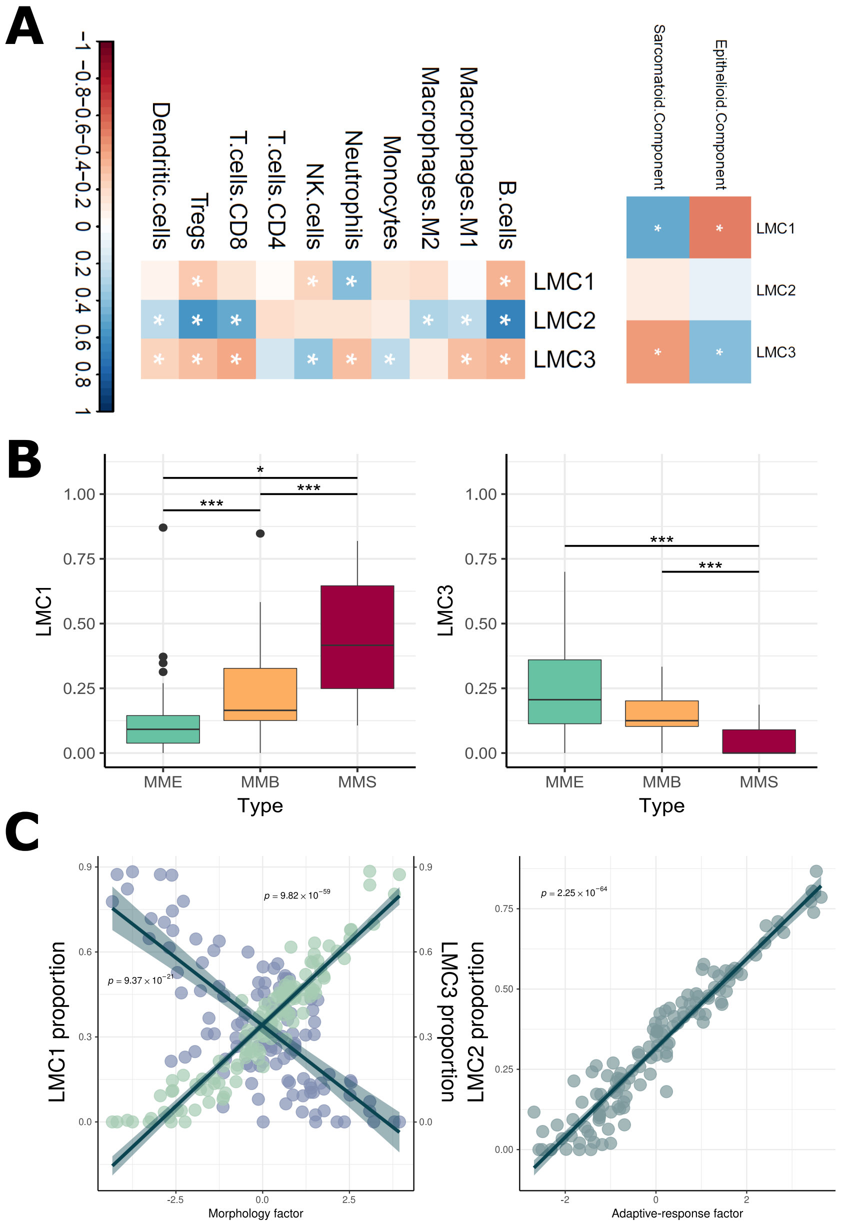

Figure S2. Reference-free deconvolution (MeDeCom) of MESOMICS enhancer DNA methylation data. A) Correlation between proportion of each inferred latent methylation component (LMC) and the proportion of cell types within a sample (Pearson's correlation test with Benjamini-Hochberg multiple testing correction, * : q-value < 0.05). B) Proportion of LMCs 1 and 3 were significantly associated with tumour type (two-sided t-test, * : p-value < 0.05, ** : p-value < 0.01, *** : p-value < 0.001, $n = 119$ biologically independent samples). LF2 and LMC1: $p = 9.37 \times 10^{-21}$; LF2 and LMC3: $p = 2.25 \times 10^{-64}$; LF3 and LMC2: $p = 9.82 \times 10^{-59}$. Boxplots represent the median and interquantile range and whiskers the maximum and minimum values, excluding outliers. C) Correlation between LMCs 1 and 3 and MOFA's Morphology factor (left), and LMC2 and MOFA's Adaptive-response factor (two-sided Pearson correlation test). The gray bands correspond to 95% confidence intervals. MME: epithelioid; MMB: biphasic; MMS: sarcomatoid.

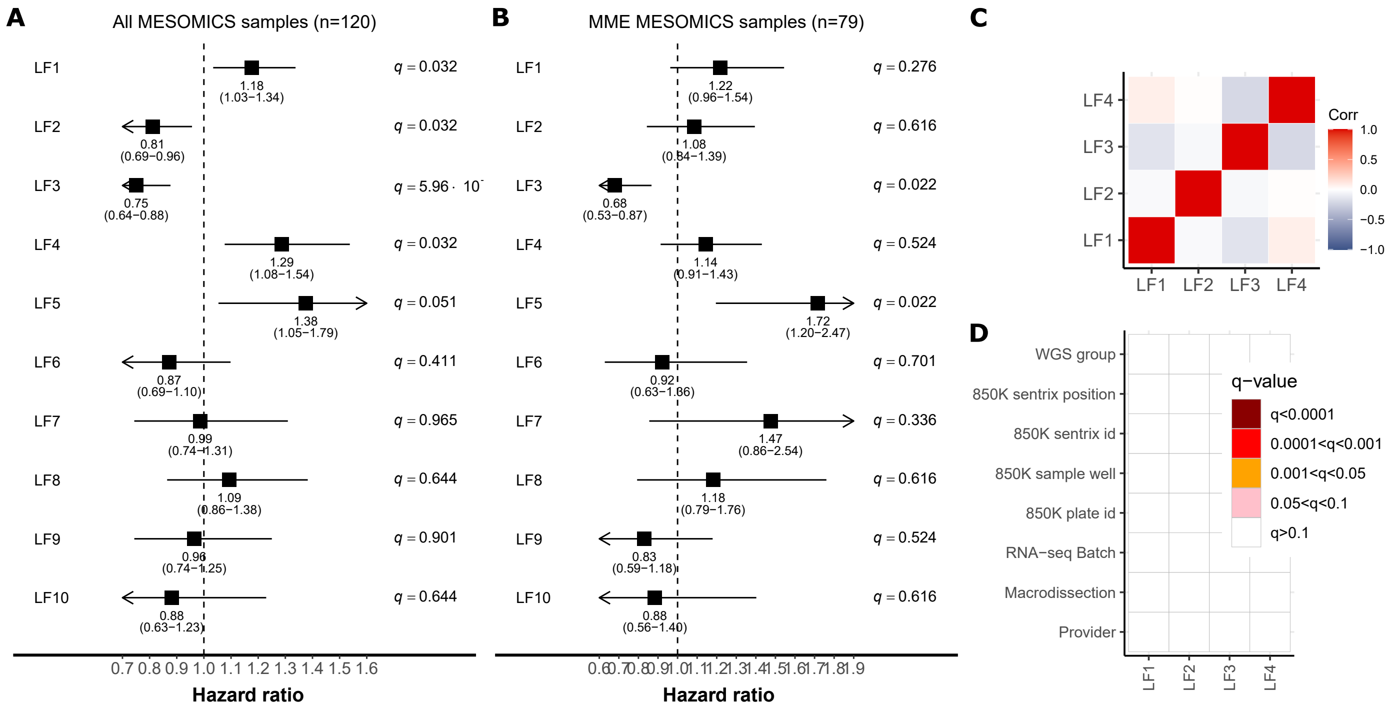

Figure S3. Association between survival and MOFA latent factors in the MESOMICS cohort. A) Forest plot of the survival analysis based on the ten MOFA latent factors (LFs), using a Cox proportional hazards model with LFs as continuous explanatory variables for (A) all 120 MESOMICS and (B) MME only samples ($n = 79$). The black square represents estimated hazard ratios and whiskers represent the 95% confidence intervals. Adjusted Wald test p-values are shown on the right. See corresponding data in Supplementary Table 15. C) Pearson correlation coefficients between factors. D) Linear regression test significance (q-value) between LFs (row) and each technical variable (column).

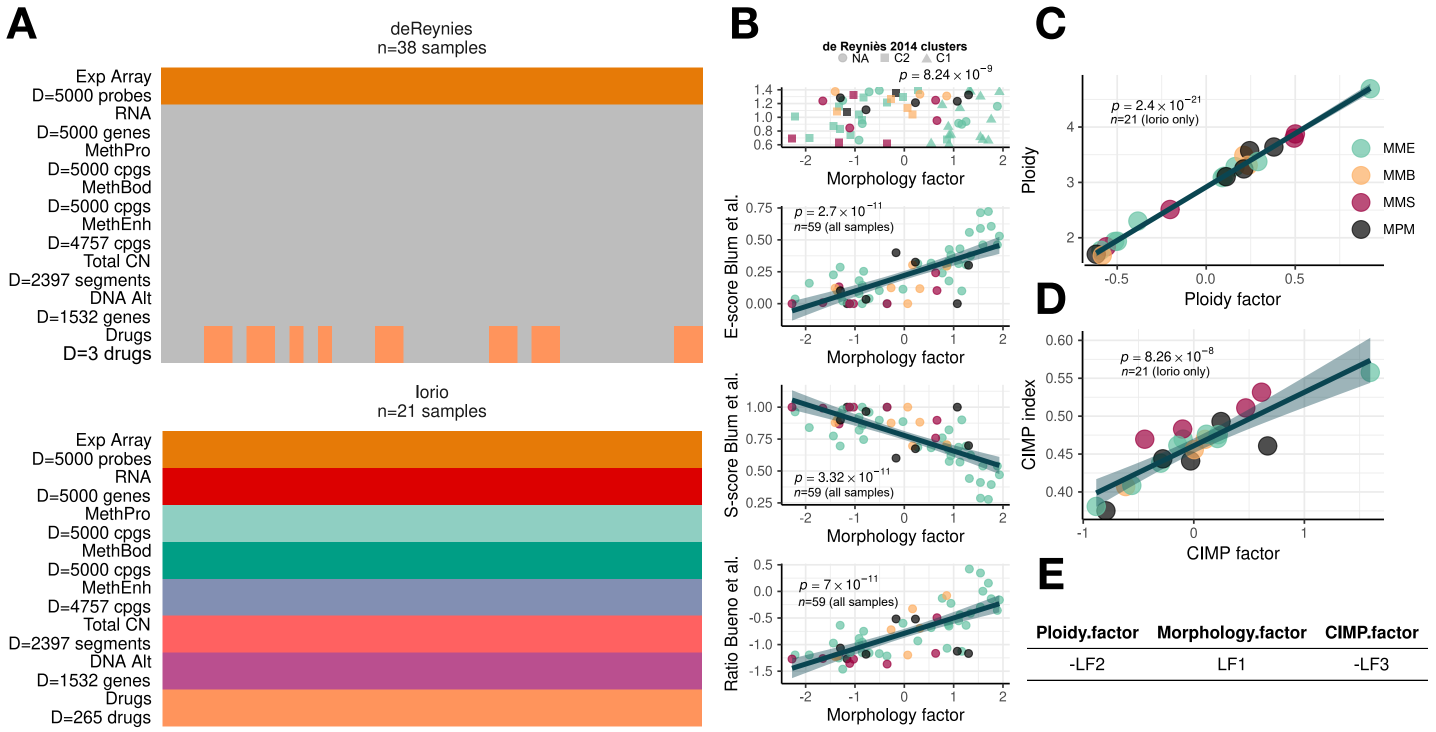

Figure S4. Overview of the MPM cell lines dataset for integration with MOFA. A) Overview of the omic data sets integrated into MOFA (design follows that of Figure S1). B) Association between MOFA cell lines morphological factor and the previously proposed molecular classifications. C-D) Association between ploidy and Ploidy factor, and between CpG island methylator phenotype (CIMP) index and CIMP factor, respectively. E) Correspondance between MOFA cell lines LFs and the MESOMICS MOFA LFs from Figure 1. Sample sizes (n) and cohort of origin (Iorio or de Reyniès) are mentioned in each scatter plot. Pearson correlation coefficients and the associated two-sided p-values are displayed in B,C and D, and gray bands correspond to 95% confidence intervals. The p-values in B top panel corresponds to an ANOVA F-test is presented. DNA Alt: rearrangements and mutations; Minor CN: minor segmental copy number; Total CN: total segmental copy number; MethEnh: DNA methylation level at enhancer regions; MethBod: DNA methylation level at body regions; MethPro: DNA methylation level at promoter regions; RNA: gene expression level.

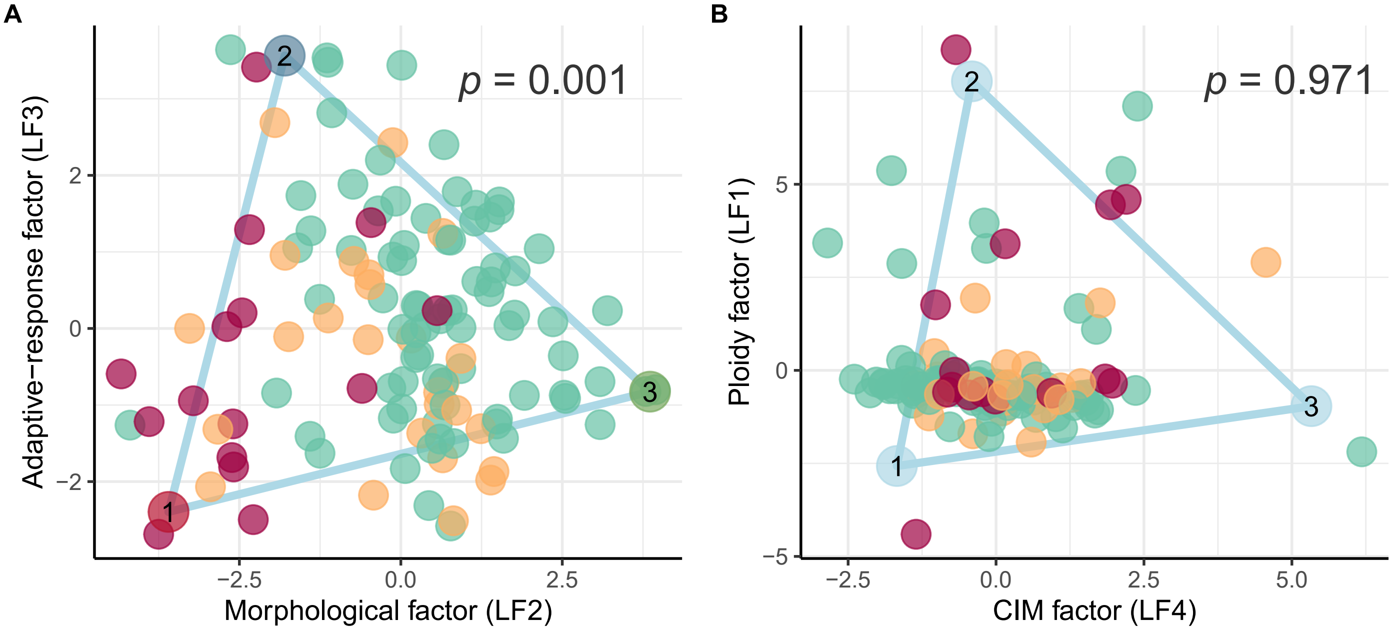

Figure S5. Comparison of the best Pareto fit in (A) morphological factor (LF2) and adaptive-response factor (LF3) space and (B) ploidy factor (LF1) and CIMP factor (LF4) space. The scatter plots represent sample positions along each factor and coloured vertices the position of the archetype defined by the ParetoTI algorithm in each space. *p*-values correspond to one-sided permutation tests on the *t*-ratio statistic. Green, orange, and red dots represent MME, MMB, and MMS samples respectively.

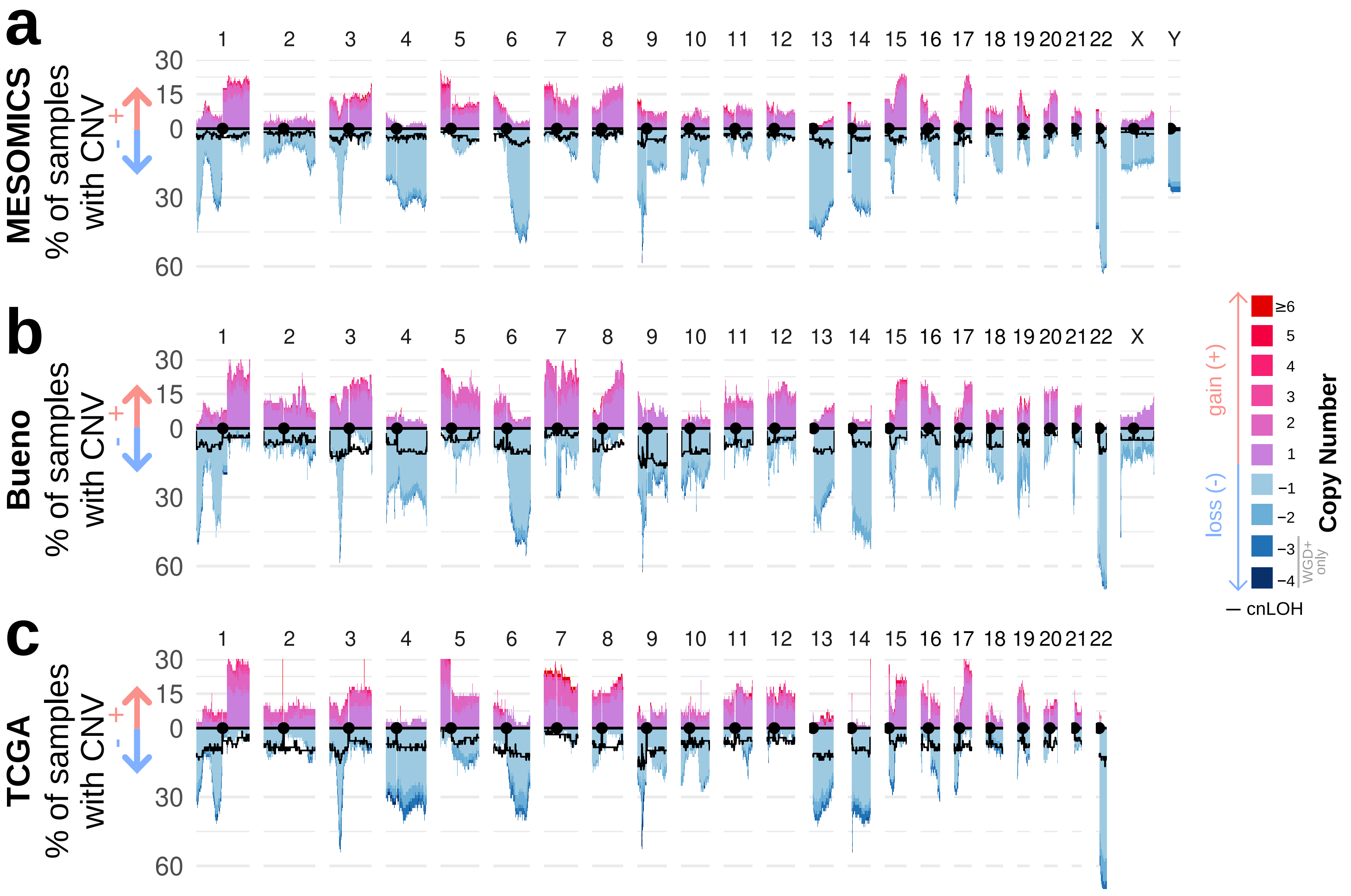

Figure S6. Copy Number profile for major MPM cohorts. Highly-consistent copy number variant (CNV) profiles across A) MESOMICS (called with purple from WGS data), B) BUENO (called with facets from WES data), and C) TCGA (called with ABSOLUTE from SNP6.0 arrays) MPM cohorts. Note that facets does not cover the Y chromosome and the TCGA study did not report sex chromosome CNVs.

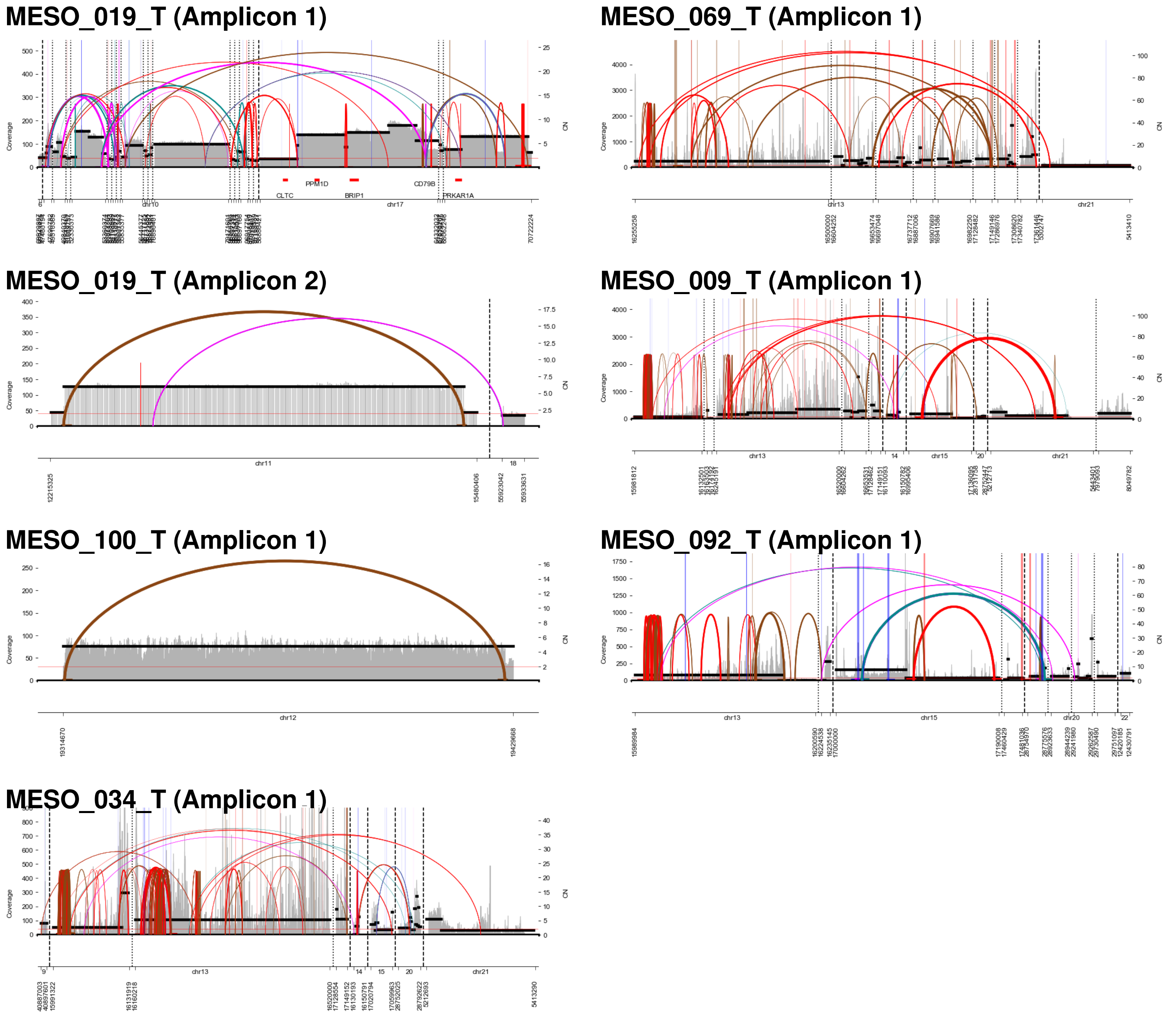

Figure S7. Extrachromosomal DNA (ecDNA) predictions for MESOMICS cohort. Amplicon Architect predicted a total of 6 posititive ecDNA samples. For each amplicon, we display the name of the sample and the identifier of the amplicon at the top. The x-axis represents the set of amplicon intervals, with vertical lines indicating break points (thin dotted line for intra-chromosomal breaks, thick dashed line for inter-chromosomal breaks), with left and right breakpoint positions indicated by the ticks. The y-axis represents both a histogram of window-based depth of coverage across (grey, left scale) and the copy number estimate of these segments (black horizontal segments, right scale). Discordant read pair clusters are represented as colored arcs, with color representing the orientation of the reads. Red: length discordant in expected orientation (forward-reverse); brown: inverted read pairs (reverse-forward); teal: both reads map to forward strand; magenta: both reads map to the reverse strand. Vertical blue lines indicate connections to source vertex. The bottom panel represent oncogene positions if any overlap with the segments.

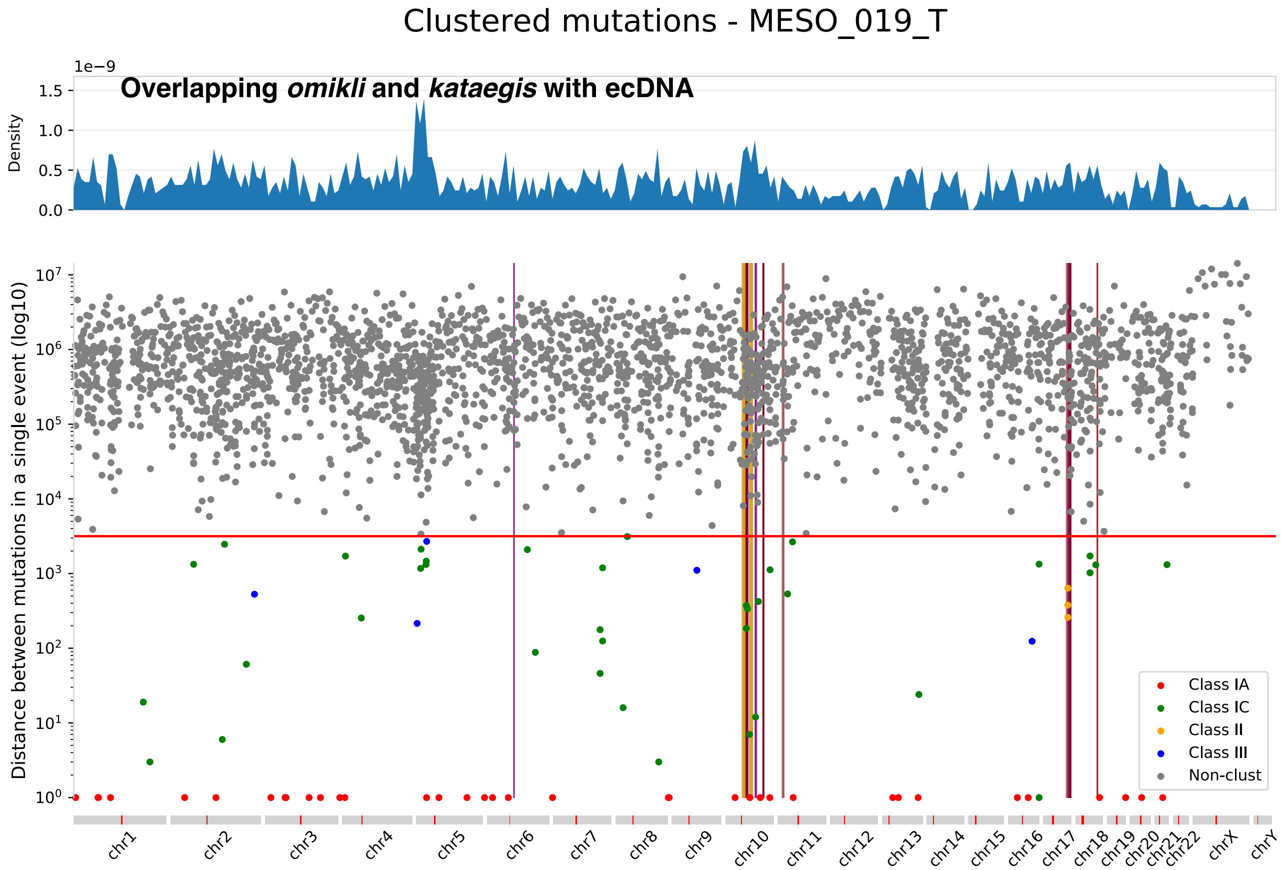

Figure S8. Example of co-occurrence of kataegis and ecDNA. Characterization and classification of clustered point mutations of one sample with co-occurrence of kataegis and ecDNA (MESO_019_T).

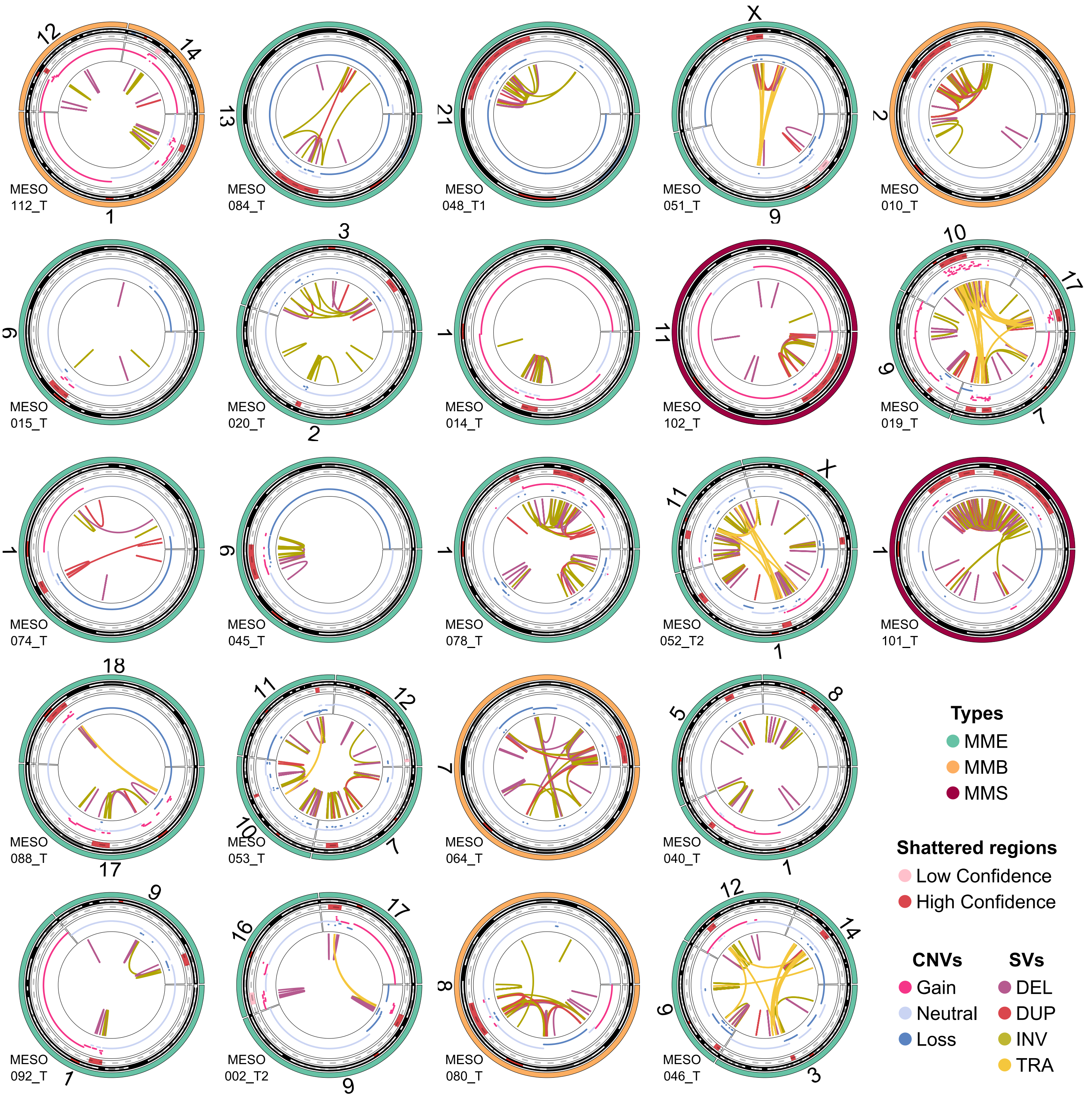

Figure S9. Chromothripsis analysis of MESOMICS cohort. Chromothripsis analysis combining Structural Variants (SVs) and Copy Number Variants (CNVs) detected a total of 23 positive samples, ordered from least number of CNs and SVs (top left) to most CNs and SVs (bottom right).

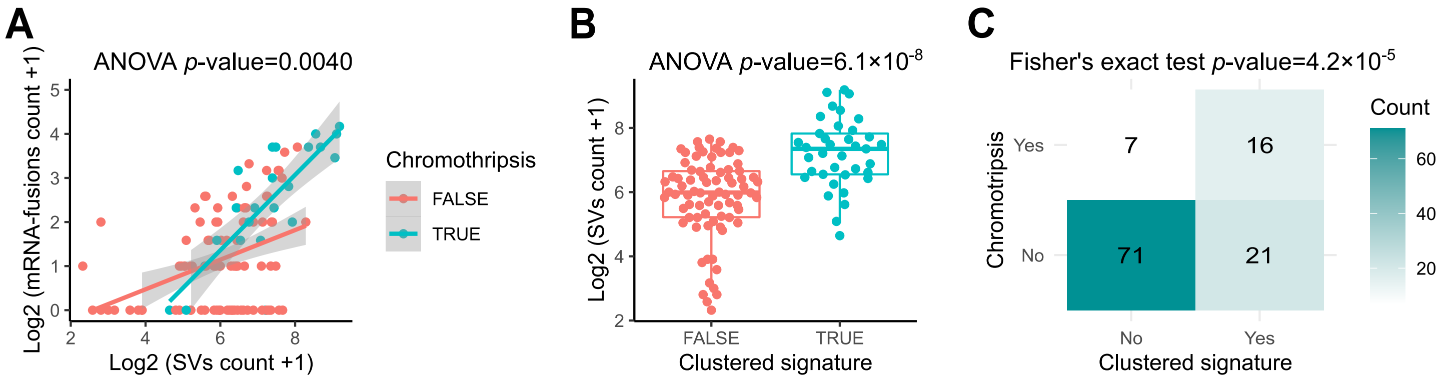

Figure S10. Chromothripsis, mRNA fusions, and clustered SV signature. A) MPM tumors with chromothripsis tend to have more mRNA fusions per structural variant. The p-value corresponds to an ANOVA F-test of log2(SV count +1) as a function of the log2(mRNA-fusion count +1) and the presence of chromothripsis across all $n = 115$ tumors of the MESOMICS cohort. Gray bands correspond to 95% confidence intervals. B) Association between clustered SV signature and SV load. The p-value corresponds to an ANOVA F-test of log2(SV count +1) as a function of the presence of the clustered SV signature across all 115 samples. Boxplots represent the median and interquantile range and whiskers the maximum and minimum values, excluding outliers. C) Association between clustered SV signature and chromothripsis. The p-value corresponds to a two-sided Fisher's exact test of the presence of the clustered SV signature and the presence of chromithripsis.

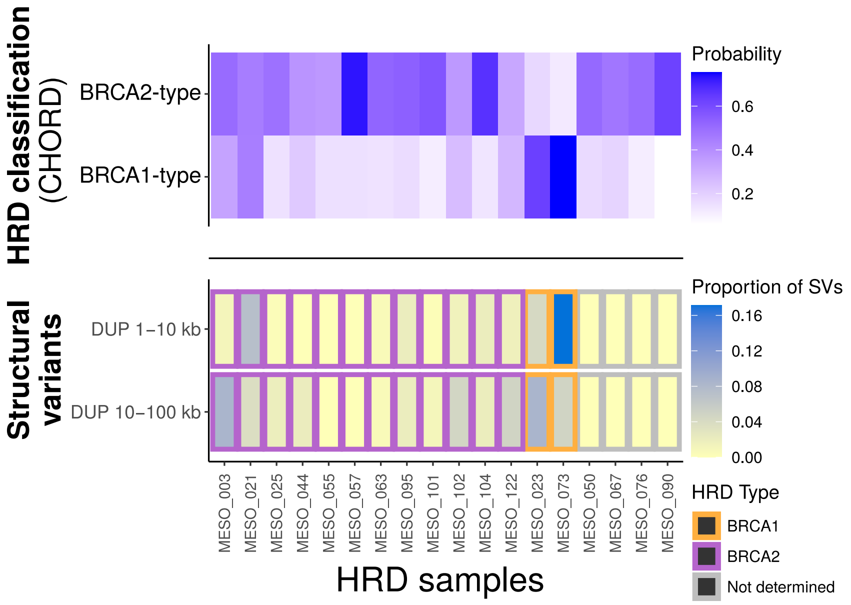

Figure S11. Overview of homologous recombination-deficiency (HRD) samples predicted by CHORD. The upper panel displays the probability for BRCA1 and BRCA2 HRD types. The lower panel indicates the proportions of duplications (DUP), binned by length (1-10kb and 10-100kb) out of all SVs, observed in each positive HRD sample and the predicted HRD type classes.

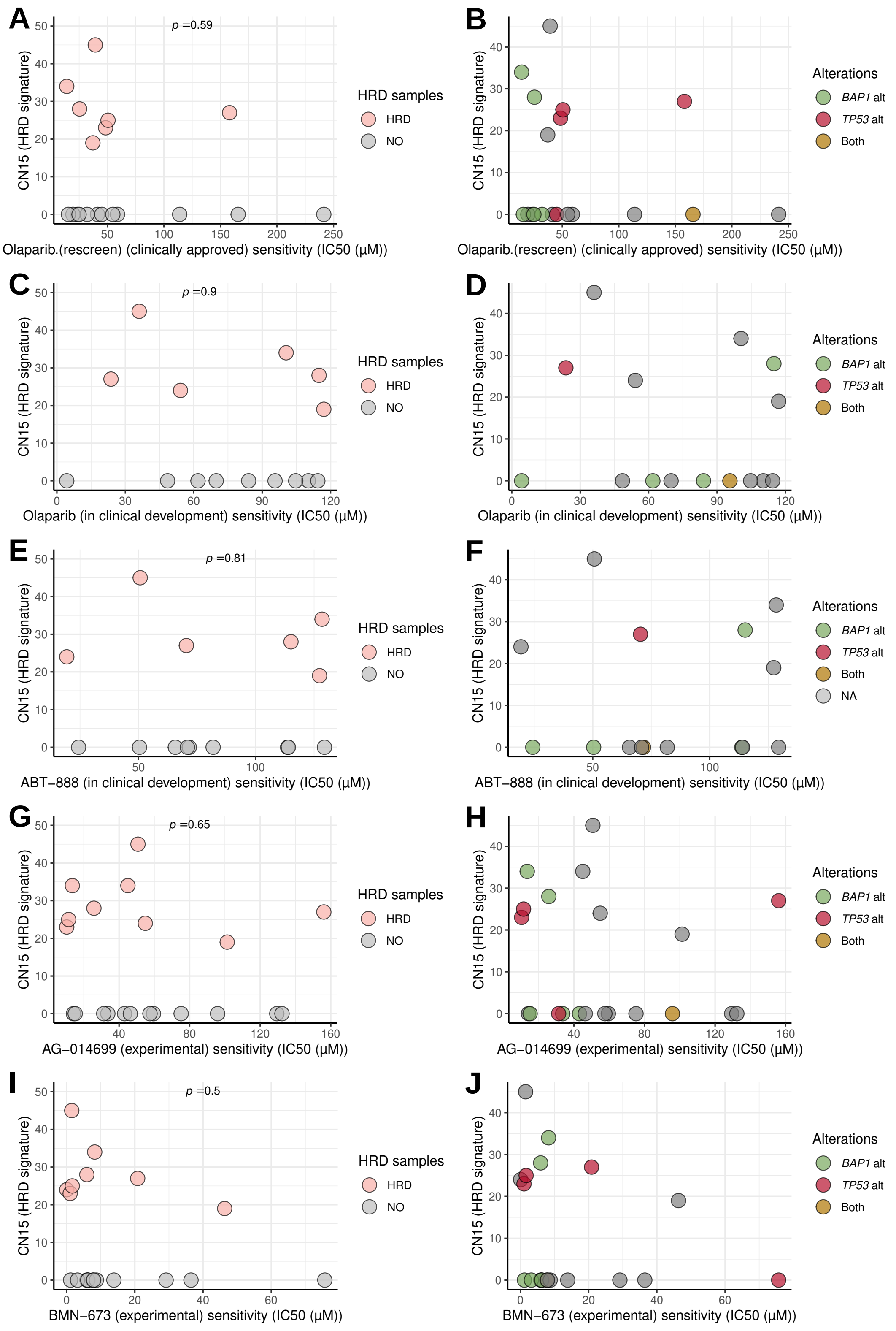

Figure S12. Homologous recombination-deficiency pattern in MPM cell lines and PARP inhibitor response (IC50 in uM). Association between PARP inhibitors (PARPi) sensitivity and the CN signature of homologous recombination-deficiency (HRD). Point colours in panels (A), (C), (E), (G), and (I) represent samples with an HRD pattern detected, and p-values correspond to linear regression (two-sided t-tests) between HRD status and PARPi response. Point colours in panels (B), (D), (F), (H), and (J) represent *BAP*1 and *TP*53 mutational status.

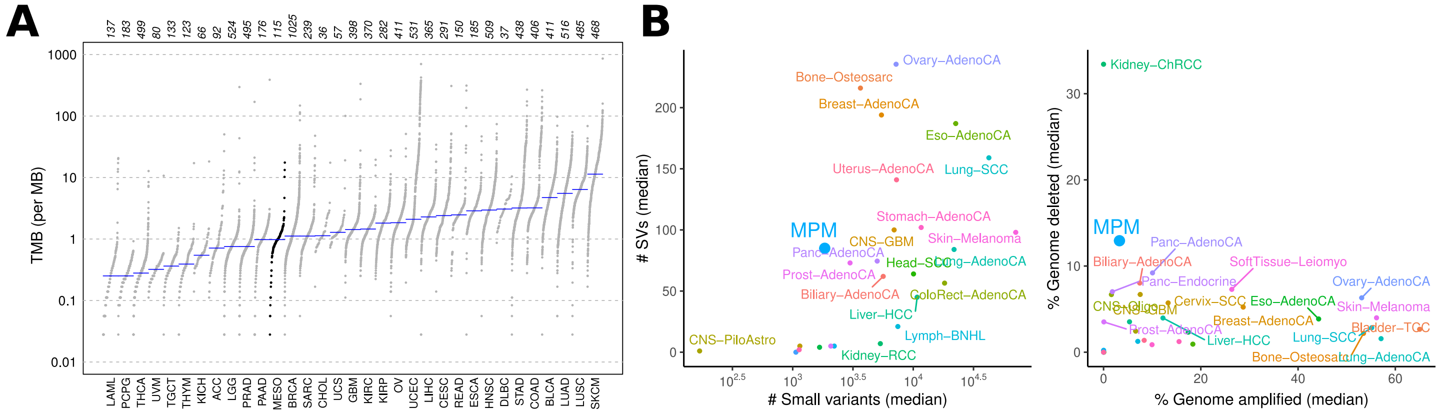

Figure S13. Comparative analysis of tumor mutational burden. A) Tumor Mutational Burden (TMB) of Mesothelioma and TCGA tumors. The number of independent biological samples is specified at the top (e.g., $n = 115$ for MESO). B) Comparison of the mutational load between mesothelioma ($n = 115$) and tumor-types from the Pan Cancer Analysis of Whole Genome data (25 cohorts from the PCAWG with at least 30 non-excluded samples, $n = 2622$). Left: median number of Structural Variants (SVs) as a function of the median number of small variants per tumor type. Right: median percentage of the genome affected by amplifications and deletions per tumor type.

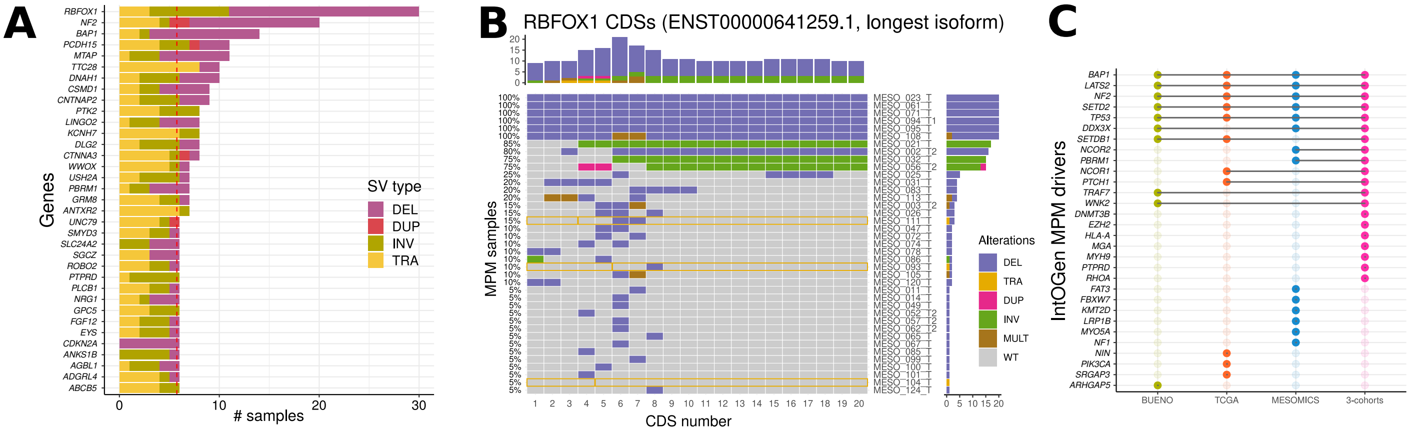

Figure S14. MPM drivers. A) Recurrent genes affected by SVs in the MESOMICS cohort. B) Structural and CNV variants affecting the coding regions of the *RBFOX1* gene. C) IntOGen MPM drivers (based on SNVs and small indels) identified within each individual MPM cohort (Bueno, TCGA, and MESOMICS) and in the pooled cohort (3-cohorts). The upset plot represents the intersections between the four sets of drivers.

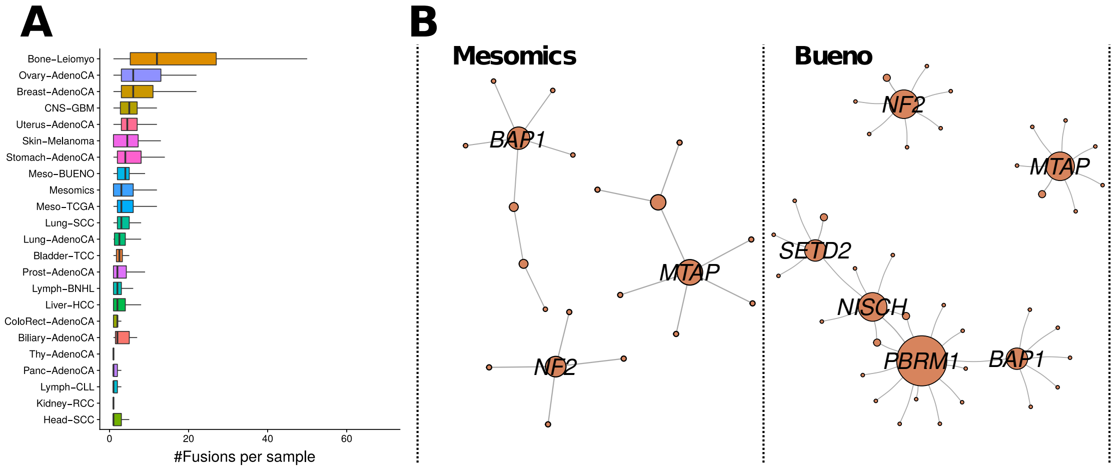

Figure S15. Comparative analysis of mRNA fusions per tumor types and mRNA-fusion network for major MPM cohorts. A) Number of mRNA fusions per tumor type in $n = 1454$ tumors from the PCAWG consortium. Boxplots represent the median and interquantile range and whiskers the maximum and minimum values, excluding outliers. B) recurrent mRNA-fusion network for MESOMICS ($n = 109$), Bueno ($n = 211$) and TCGA ($n = 73$) cohorts.

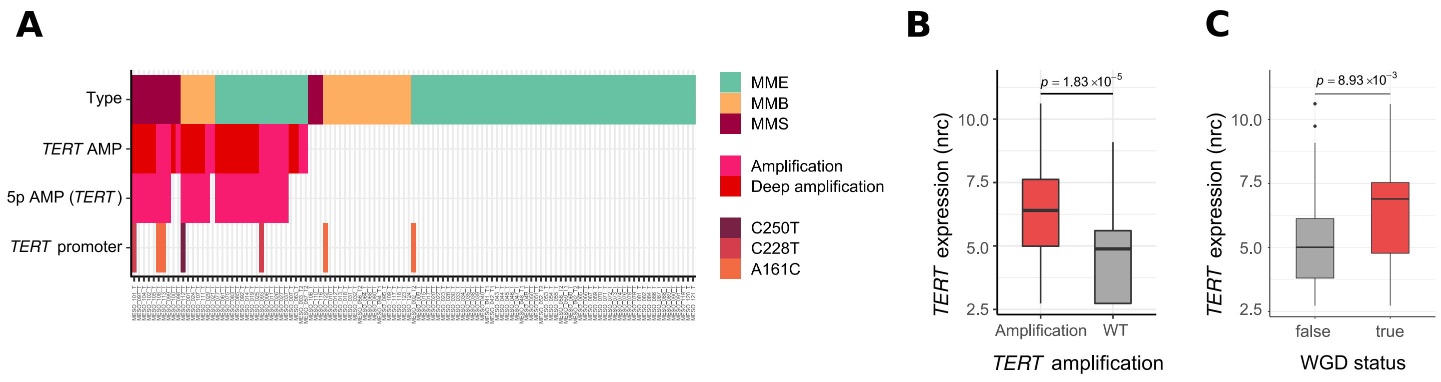

Figure S16. Overview of *TERT* alterations in the MESOMICS samples. A) Oncoplot of the different types of alterations affecting *TERT* and its chromosomal arm. B) Impact of *TERT* amplification on gene expression (in normalized read counts, nrc, $n = 99$). C) Impact of WGD on *TERT* gene expression ($n = 105$). In (B)-(C), boxplots represent the median and interquantile range and whiskers the maximum and minimum values, excluding outliers, and p-values correspond to two-sided t-tests.

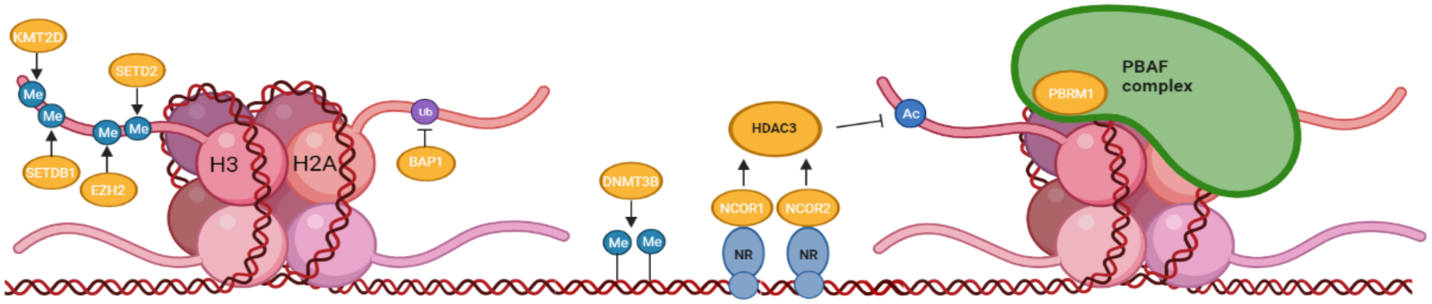

Figure S17. Schematic representation of mesothelioma driver epigenetic regulatory gene (ERG) functions. Histone methyltransferases KMT2D, SETDB1, EZH2 and SETD2 methylate histone positions H3K4, H3K9, H3K27 and H3K36 respectively (Me), DNA methyltransferase DNMT3B maintains DNA methylation marks (Me), BAP1 deubiquitinates H2AK119 (Ub), NCOR1 and NCOR2 bind to nuclear receptors (NR) as part of corepressor complexes and recruit histone deacetylase HDAC3, and PBRM1 binds acetylated histone tails (Ac) as part of the PBAF chromatin remodelling complex (created with BioRender).

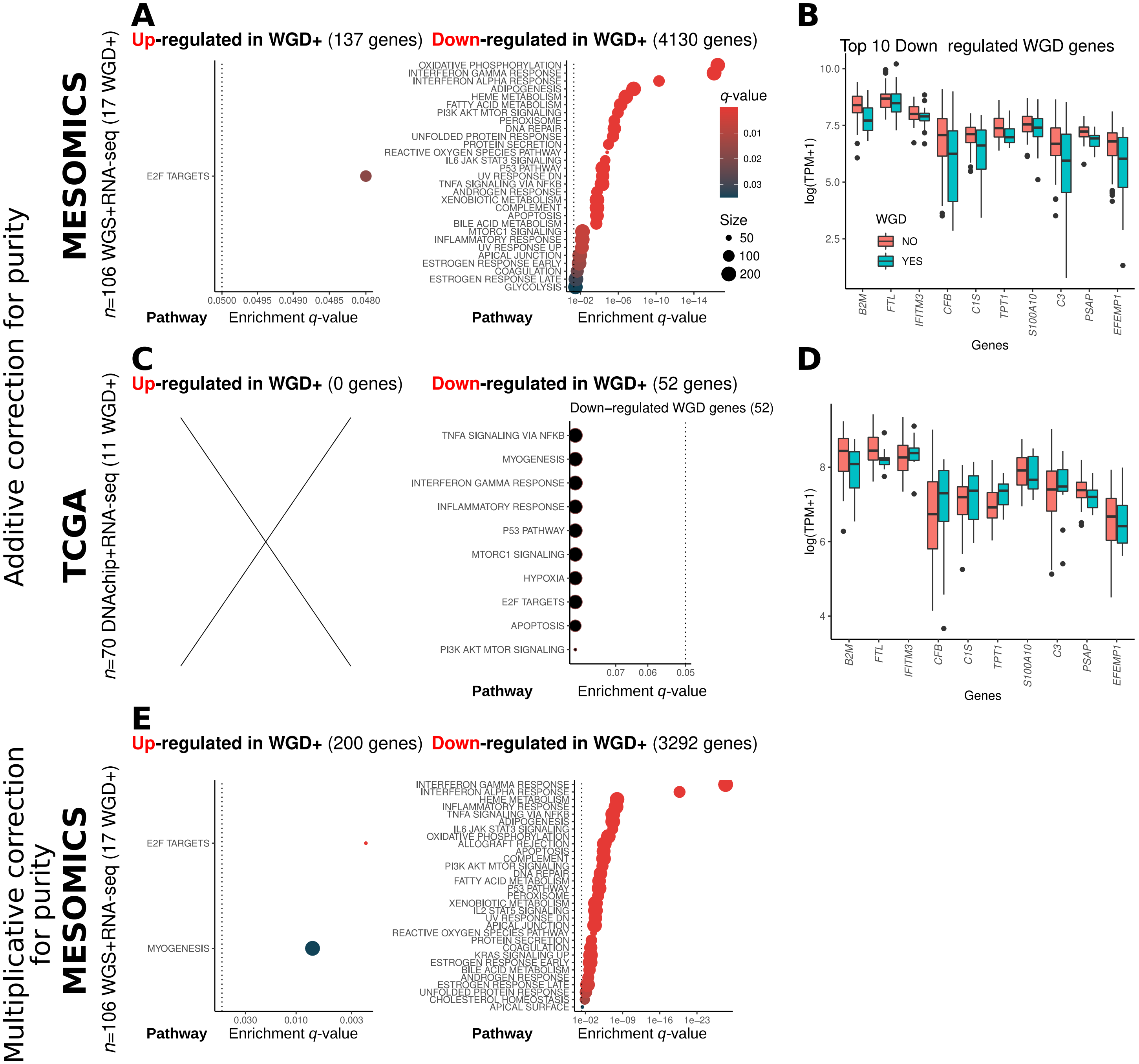

Figure S18. Pathways and top down-regulated genes of whole-genome doubling (WGD)+ samples. A) Pathway enrichment analysis (hypergeometric test) of differentially-expressed genes in WGD compared to non-WGD tumors from the MESOMICS cohort (106 samples with both WGS and RNA-seq). B) Top 10 down-regulated genes of WGD+ samples of MESOMICS cohort. C)-D) Replication of (A) and (B) in the TCGA cohort (70 samples with CNV calls from DNA chip and RNA-seq). Note that no genes/pathways were found to be up-regulated at the *q*-value threshold of 5%, so the left side of panel (C) is left empty. In (B) and (D), boxplots represent the median and interquantile range and whiskers the maximum and minimum values, excluding outliers. E) Same as panel (A), but accounting for multiplicative effects of purity on gene expression. TPM: Transcripts Per Kilobase Million.

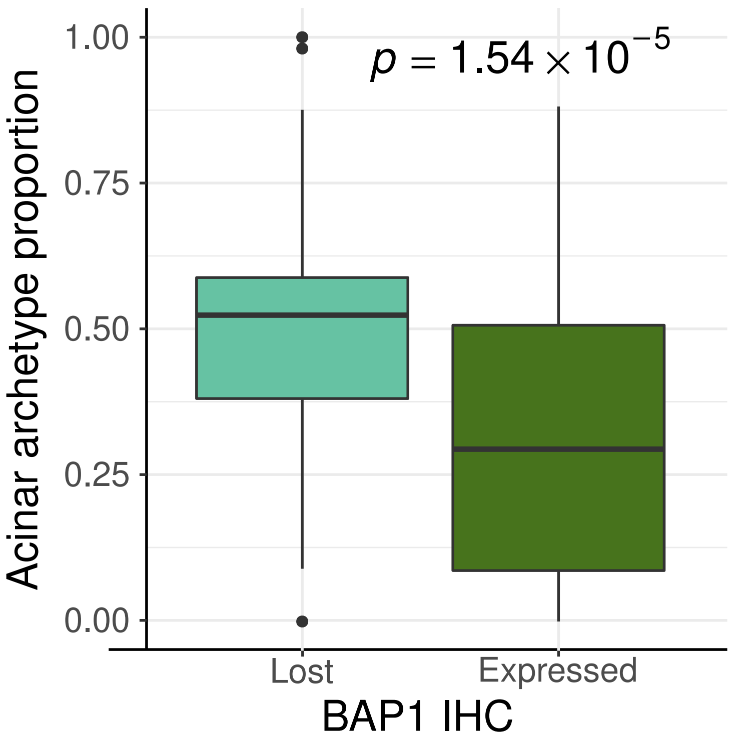

Figure S19. Relationship between acinar phenotype and BAP1 protein expression measured by immunohistochemistry (IHC). p-values correspond to an ANOVA F-test ($n = 110$ biologically independent samples). Boxplots represent the median and interquantile range and whiskers the maximum and minimum values, excluding outliers.

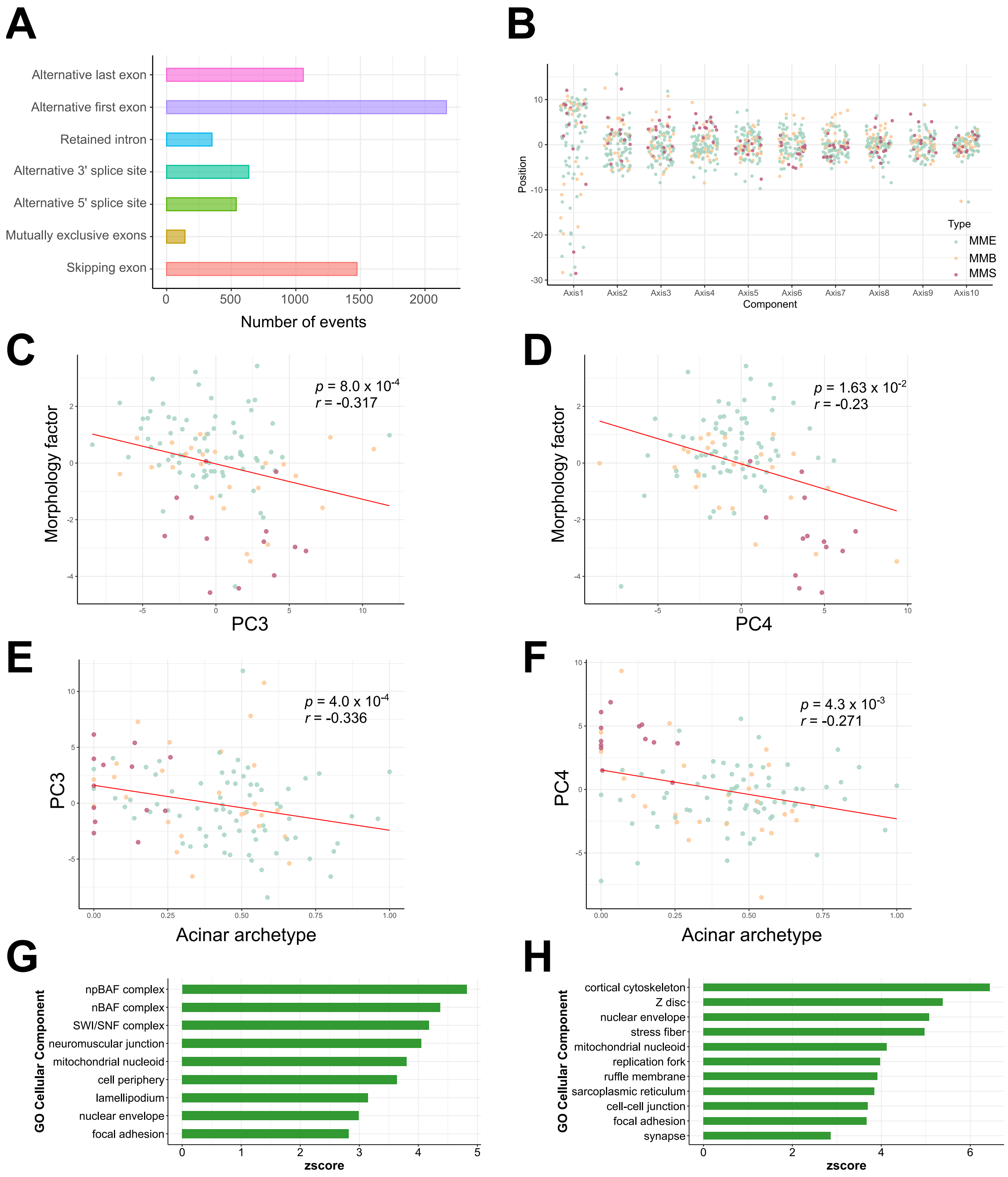

Figure S20. Alternative splicing in malignant pleural mesothelioma. The top most variable alternative splicing events (those which contributed 50% to total variation, n =6,366) within the MESOMICS cohort (n =109) were selected for principal components analysis. A) Distribution of the top 6,366 detected alternative splicing events (ASE) by category. B) Sample distribution along principal components 1-10 calculated from 6,366 ASEs, coloured by tumour type. C)-D) Association between principal components and MOFA Morphology factor, and (E)-(F) proportion of Acinar Archetype; p-values correspond to Pearson correlation tests. G)-H) Gene Ontology Cellular Component analysis of genes affected by ASEs contributing to principal components 3 (G) and 4 (H).

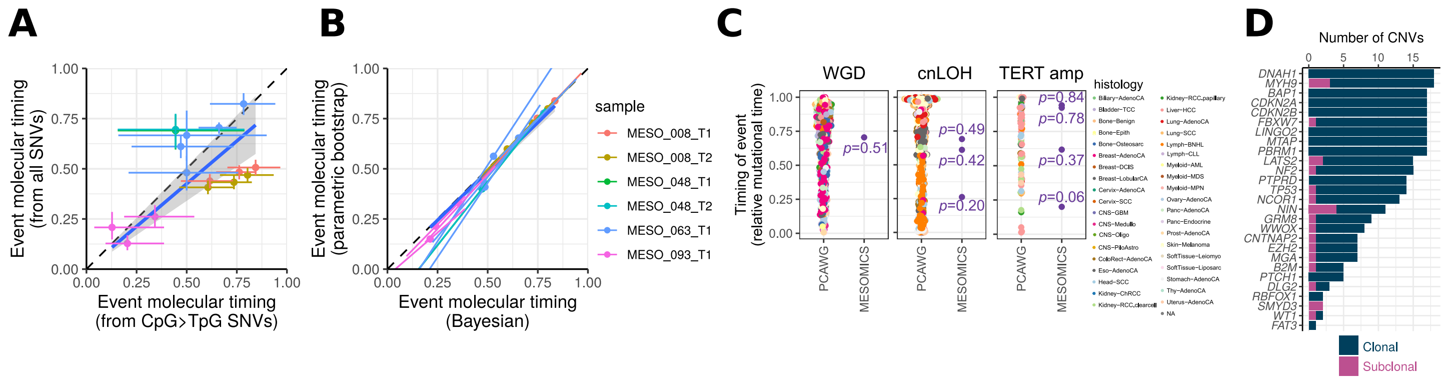

Figure S21. Timing of copy number gains in mutation time. A) Comparison of copy gain timing estimates based on CpG to TpG mutations and based on all mutations. Points represent point estimates for an event and a tumor sample, and segments 95% Bayesian confidence interval (CI). Samples included either underwent a WGD event (MESO_008 and MESO_063), or a large-scale loss of heterozygosity (LOH) allowing timing (MESO_048, MESO_093). B) Comparison of copy gain timing CI using Bayesian inference or parametric bootstrapping. Points represent the centers of CI, and segments 95% CI. Samples included correspond to that presented in panel D. In (A)-(B), gray bands correspond to 95% confidence intervals C) Timing of copy number gains in mutation time in the MESOMICS cohort compared to the >2500 tumors from the Pan-Cancer Whole-Genome Consortium (PCAWG, Gerstung *etal.* 2020). Points represent tumor sample estimates, and *p*-values the empirical *p*-values with the timing of event used as test statistic, and using the empirical distribution of timings of similar events in the PCAWG tumors as null distribution. D) Clonality of CNVs affecting driver MPM genes.

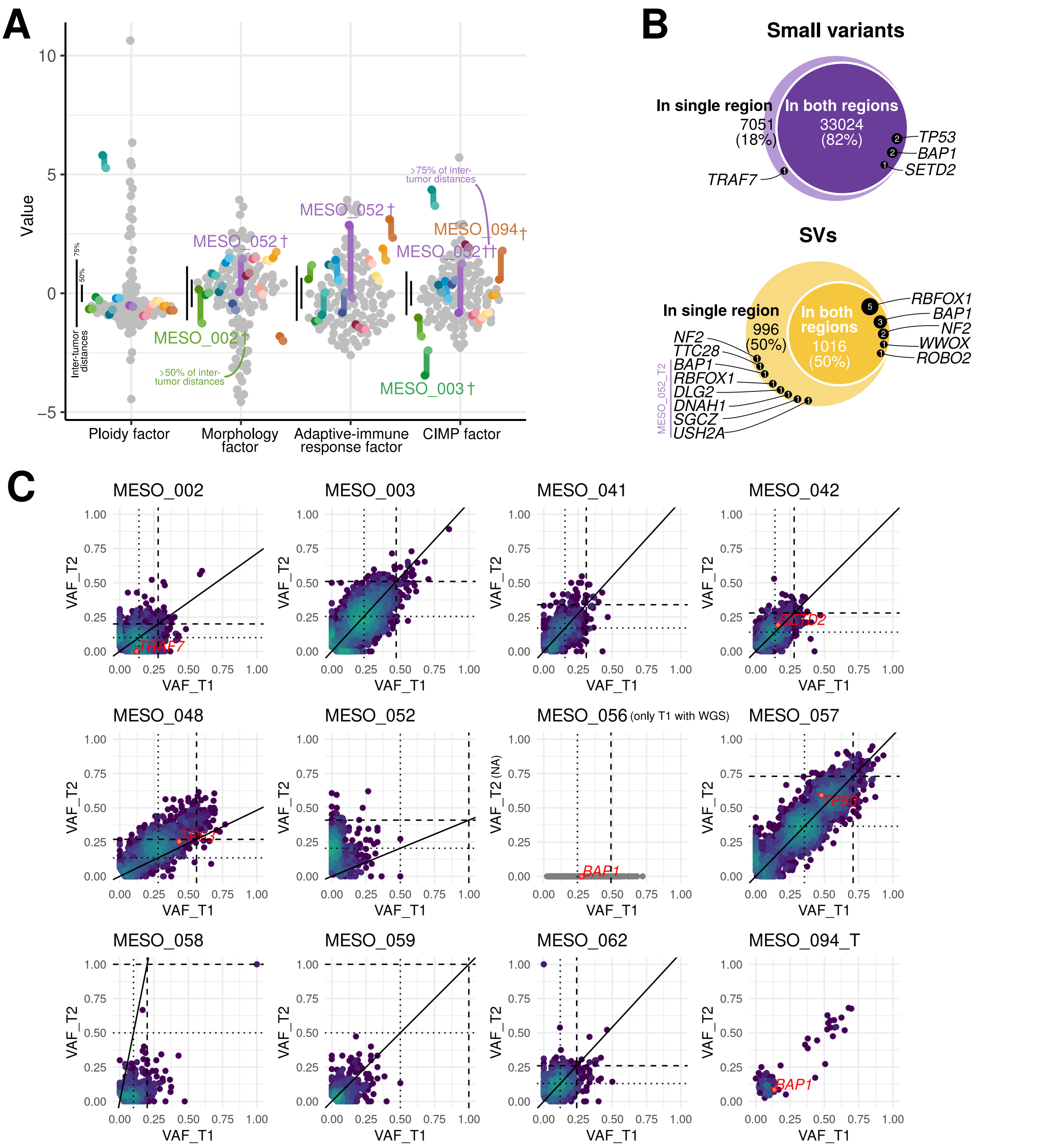

Figure S22. Patterns of intra-tumor heterogeneity in 13 samples. A) Position of multi-regional samples in MOFA LFs. Colors represent samples (gray: samples with a single region sequenced; colors: multi-regional samples), segments connect regions from the same tumor. Black bars on the left of each plot represent the empirical 50% and 75% percentiles of the distribution of pairwise inter-tumor distances, and tumors with an intra-tumor distance in the range [50%, 75%) and [75%, 100%) are annotated by a † and ††, respectively. Note that samples with missing genomic data are not represented on Factor 1. B) Venn-Euler diagrams of small variants (top) and SVs (bottom), indicating which alterations are found in a single or both regions. Driver mutations as highlighted in **Figure 4** are mentioned, with numbers corresponding to the number of such alterations among the 13 samples. C) Joint distribution of variant allele frequencies (VAFs) for the 12 multi-regional samples with WGS data (out of 13). Point colors: density measured by the number of points within a fixed radius. Damaging alterations in driver genes are represented by red points and text. Dashed lines: expected VAF for clonal alterations in diploid regions given tumor purity; dotted line: expected VAF for clonal alterations in haploid regions given tumor purity; solid line: ratio of tumor purities estimated from WGS data.

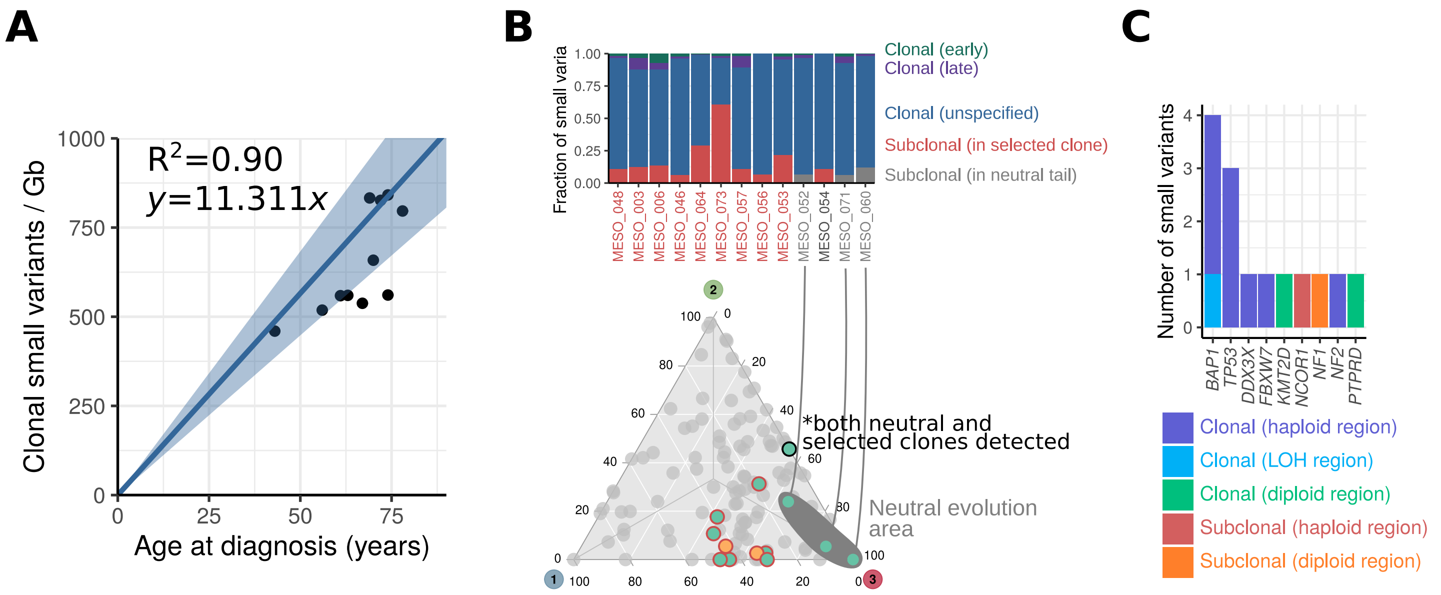

Figure S23. Timing of small variants. A) Analysis of the small variant mutational burden as a function of age at diagnosis. The line denotes the mean and standard error of a linear regression model without intercept (R^2 and best fit equation are mentioned in the plot). B) Neutral evolution detected from the VAF distribution, and corresponding proportions of small variants belonging to selected (red), neutral subclones (gray), or clonal (blue, purple, and green) (top), and position of samples in the Pareto front. C) Clonality of driver small variant mutations; "early" indicates that the alteration predates the LOH in the corresponding region. B)-(C) display 13 high-purity samples where clonal reconstruction was possible (see list in panel B).

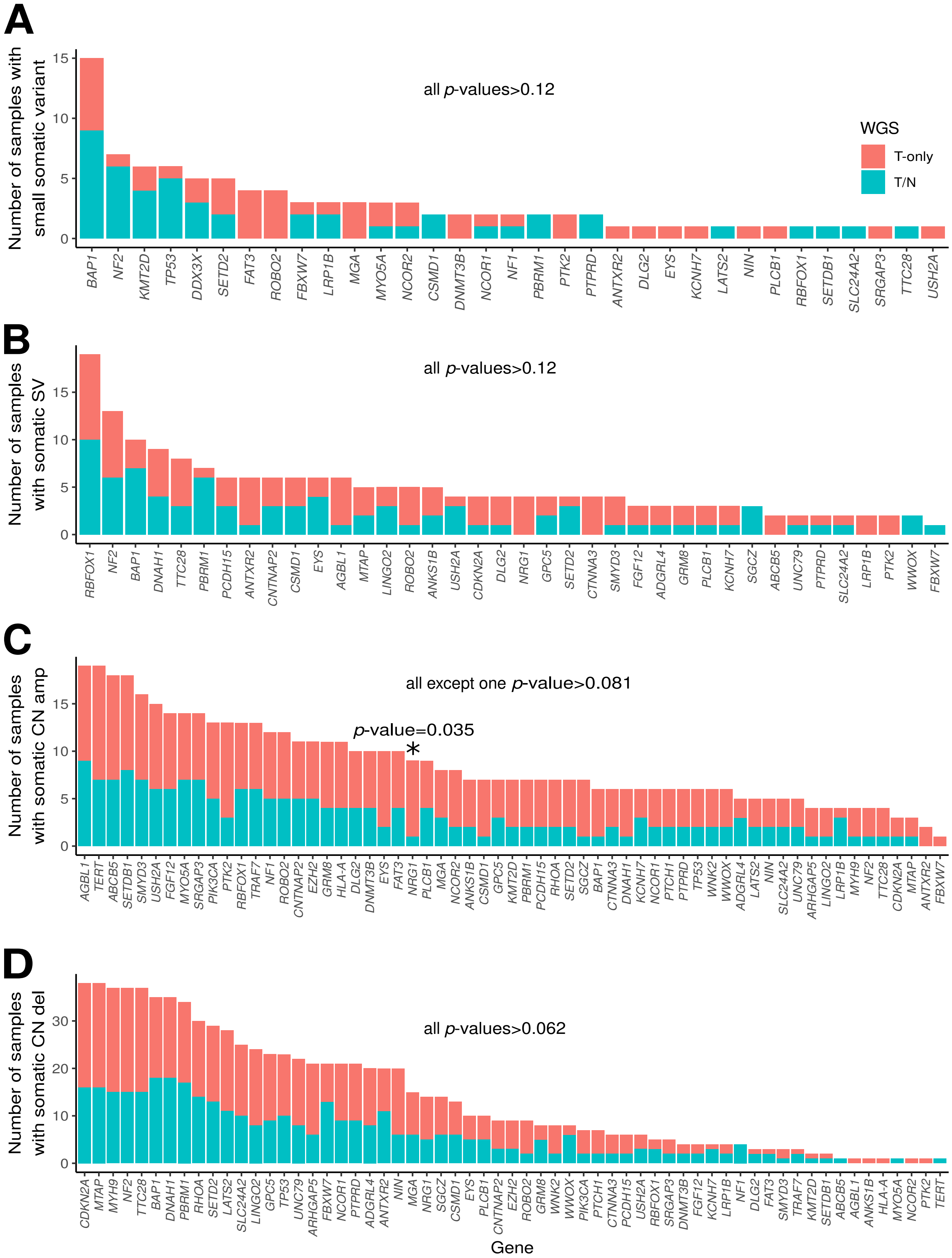

Figure S24. Comparison of the number of alterations in gene drivers between T/N matched samples and tumor-only samples of the epithelioid type (*n*=77 samples, 38 T-only and 39 T/N). A) Small variants. B) Structural variants. C) Copy number amplifications. D) Copy number deletions. *p*-values correspond to Fisher's exact tests.

3 List of Supplementary Tables

Table S1. MESOMICS cohort overview and comparison with TCGA and Bueno cohorts.
Table S2. MESOMICS samples overview.
Table S3. MESOMICS samples overview dictionary.
Table S4. MOFA MESOMICS and replication in the TCGA and Bueno cohorts
Table S5. Variance explained by MOFA latent factors in the MESOMICS cohort, and replication in the TCGA and Bueno cohorts
Table S6. Associations between MOFA and technical, clinical, morphological, exposure variables in the MESOMICS cohort, and replication in the TCGA and Bueno cohorts
Table S7: Molecular scores and features in the MESOMICS, Bueno, and TCGA cohorts.
Table S8: NMF of methylation data in the MESOMICS cohort and replication in the TCGA cohort.
Table S9. Differential expression analysis between WGD- and WGD+ samples in the MESOMICS cohort and replication in the TCGA cohort
Table S10. Gene Set Enrichment Analysis (GSEA) between WGD- and WGD+ samples in the MESOMICS cohort and replication in the TCGA cohort
Table S11. Tumor suppressor genes (TSG) and CIMP-index in the MESOMICS cohort and replication in the TCGA cohort
Table S12: Univariate survival analyses.
Table S13. Epithelioid subtypes and survival.
Table S14. Treatment and survival.
Table S15. MOFA factors association with survival.
Table S16. Prognostic scores from Bueno et al. and Blum et al.
Table S17. Survival model fits in the MESOMICs cohort.
Table S18. Survival model prediction tests in the TCGA cohort.
Table S19. Survival model AUC in the MESOMICS cohort using cross-validation.
Table S20. Survival model AUC in the TCGA cohort using bootstrapping.
Table S21. Summary of all p- and q-values from the discovery (MESOMICS) and replication cohorts
Table S22. Details of the replication of p-values and q-values listed in the text
Table S23. MOFA factors in cell lines.
Table S24. Variance explained by MOFA factors in cell lines.
Table S25. Weights of drug response in MOFA latent factors from cell lines.
Table S26. Association of drug response with MOFA latent factors in cell lines.
Table S27. Principal Component Analyses of the transcriptome in the MESOMICS cohort, and replication in the TCGA and Bueno cohorts
Table S28. Archetypes in the MESOMICS cohort, and replication in the TCGA and Bueno cohorts.
Table S29. Association of archetypes with technical, clinical, morphological, and exposure variables in the MESOMICS cohort, and replication in the TCGA and Bueno cohorts.
Table S30. Integrated Gene Set Enrichment Analysis (IGSEA) of archetypes in te MESOMICS cohort, and replication in the TCGA cohort.
Table S31. Amplification and deletion broad events (GISTIC2).
Table S32. Amplification and deletion peaks location (GISTIC2).
Table S33. Amplification and deletion peaks per sample (GISTIC2).
Table S34. Copy Number Variants (CNVs).
Table S35. Copy number variant burden and signatures.
Table S36. Amplifications and deletions in major driver genes.
Table S37. CNV clonality.
Table S38. Amplicons.
Table S39. Chromothripsis.
Table S40. Homologuous Recombination Deficiency (HRD), aneuploidy, and clustered SNVs.
Table S41. Structural Variants (SVs).
Table S42. SV burden and SV signatures.
Table S43. Fusion transcripts
Table S44. Small variants
Table S45. SNV burden and mutational signatures.
Table S46. Germline HRD mutations.
Table S47. Association between MOFA factors and genomic events and replication in the TCGA and Bueno cohorts

Table S48. Association between Archetype proportions and genomic events and replication in the TCGA and Bueno cohorts
Table S49. Microsatellite instability
Table S50. ITH Sample Overview
Table S51. ITH analyses
Table S52. ITH MOFA Variance explained
