# Supplementary material for: Multiomic analysis of malignant pleural mesothelioma identifies molecular axes and specialized tumor profiles driving intertumor heterogeneity
Source: Nat Genet. 2023 Mar 16;55(4):607–18. doi: 10.1038/s41588-023-01321-1 (PMC10101853; doi:10.1038/s41588-023-01321-1)
Supplement: Supplementary file 2 — Reporting Summary [file 41588_2023_1321_MOESM2_ESM.pdf]

nature portfolio

Corresponding author(s): Lynnette Fernandez-Cuesta & Matthieu Foll

Last updated by author(s): Dec 2, 2022

Reporting Summary

Nature Portfolio wishes to improve the reproducibility of the work that we publish. This form provides structure for consistency and transparency in reporting. For further information on Nature Portfolio policies, see our Editorial Policies and the Editorial Policy Checklist.

Statistics

For all statistical analyses, confirm that the following items are present in the figure legend, table legend, main text, or Methods section.

n/a	Confirmed	
☐	☒	The exact sample size (*n*) for each experimental group/condition, given as a discrete number and unit of measurement
☐	☒	A statement on whether measurements were taken from distinct samples or whether the same sample was measured repeatedly
☐	☒	The statistical test(s) used AND whether they are one- or two-sided<br>*Only common tests should be described solely by name; describe more complex techniques in the Methods section.*
☐	☒	A description of all covariates tested
☐	☒	A description of any assumptions or corrections, such as tests of normality and adjustment for multiple comparisons
☐	☒	A full description of the statistical parameters including central tendency (e.g. means) or other basic estimates (e.g. regression coefficient) AND variation (e.g. standard deviation) or associated estimates of uncertainty (e.g. confidence intervals)
☐	☒	For null hypothesis testing, the test statistic (e.g. *F*, *t*, *r*) with confidence intervals, effect sizes, degrees of freedom and *P* value noted<br>*Give P values as exact values whenever suitable.*
☐	☒	For Bayesian analysis, information on the choice of priors and Markov chain Monte Carlo settings
☒	☐	For hierarchical and complex designs, identification of the appropriate level for tests and full reporting of outcomes
☐	☒	Estimates of effect sizes (e.g. Cohen's *d*, Pearson's *r*), indicating how they were calculated

Our web collection on statistics for biologists contains articles on many of the points above.

Software and code

Policy information about availability of computer code

Data collection	No software was used for data collection.
Data analysis	WGS reads mapping: in-house workflow (github.com/IARCbioinfo/alignment-nf v1.0) that used bwa version 0.7.15, GATK version 4.0.12, samblaster version 0.1.24, sambamba version 0.6.6.<br>Variant calling: Mutect2 from GATK version 4.1.5, annotation with ANNOVAR version of April 16th 2018.<br>Copy number variant calling: PURPLE version 2.52 and Gistic2 version 2.20.23.<br>Structural variant calling: SVaba version 1.1.0, Delly version 0.8.3, Manta version 1.6.0, and SURVIVOR version 1.0.7.<br>Amplicon pattern calling: AmpliconArchitect version 1.2 and CNVkit version 0.9.7.<br>RNA-seq data processing: in-house workflow (github.com/IARCbioinfo/RNAseq-nf v2.3) that used Trim Galore (version 0.6.5 for expression quantification, and version 0.4.2 for alternative splicing analyses), STAR version 2.7.3a and StringTie version 2.1.2.<br>Quality control with FastQC version 0.11.9 and RSeQC version 3.0.1.<br>Fusion transcript discovery: realignment with STAR version 2.7.6a followed by Arriba version 2.1.0.<br>Immune contexture quantification: quanTIseq version of July 2020.<br>Statistical analyses with R version 4.0.3

For manuscripts utilizing custom algorithms or software that are central to the research but not yet described in published literature, software must be made available to editors and reviewers. We strongly encourage code deposition in a community repository (e.g. GitHub). See the Nature Portfolio guidelines for submitting code & software for further information.

Data

Policy information about availability of data

All manuscripts must include a data availability statement. This statement should provide the following information, where applicable:

- Accession codes, unique identifiers, or web links for publicly available datasets
- A description of any restrictions on data availability
- For clinical datasets or third party data, please ensure that the statement adheres to our policy

The genome sequencing data, RNA-seq data, and methylation data have been deposited in the European Genome-phenome Archive (EGA) database, which is hosted at the EBI and the CRG, under accession number EGAS00001004812. Because raw -omics datasets derived from humans are at risk of re-identification when combined with information from other public sources, access must be requested to the MESOMICS data access committee (DAC) as detailed at https://ega-archive.org/studies/EGAS00001004812. Minimum datasets of processed somatic alterations for genomic, transcriptomic, and epigenomic data, sufficient to reproduce, interpret and extend our main results, are publicly available at https://github.com/IARCbioinfo/MESOMICS_data/tree/main/phenotypic_map/MESOMICS. A data note manuscript detailing all quality controls of the dataset is available at https://www.biorxiv.org/content/10.1101/2022.07.06.499003v16. TCGA whole-exome sequencing, RNA-seq, and methylation array data are available from the GDC portal (TCGA-MESO cohort), the whole-exome sequencing and RNA-seq data from the Bueno and colleagues cohort are available from the European Genome-phenome Archive, EGA:EGAS00001001563. Small variants lists, RNA-seq, expression array, and methylation data for the Iorio and colleagues cohort are available from the GEO (GSE29354), EGA (EGAS00001000828), and SRA (PRJNA523380) websites, and corresponding drug responses are available from the cancerrxgene.org website (https://www.cancerrxgene.org/downloads/drug_data?tissue=MESO; accessed July 2021). Expression array data for the de Reyniès and colleagues cohorts are available from the ArrayExpress platform (E-MTAB-1719), and corresponding drug response data from the supplementary material of Blum et al. All the other data supporting the findings of this study are available within the article and its supplementary information files.

Field-specific reporting

Please select the one below that is the best fit for your research. If you are not sure, read the appropriate sections before making your selection.

☒ Life sciences ☐ Behavioural & social sciences ☐ Ecological, evolutionary & environmental sciences

For a reference copy of the document with all sections, see nature.com/documents/nr-reporting-summary-flat.pdf

Life sciences study design

All studies must disclose on these points even when the disclosure is negative.

Sample size

This study first includes an unsupervised analysis of genomes, RNA-seq, and methylation data using group factor analysis (software MOFA). For such multivariate analyses, no simple power calculations are available, but recommendations for stable latent factors and weights such that the results from the sample can accurately be generalized to the population suggest n>100 (Saccenti and Timmerman J.Proteom. Res. 2016), which is in line with the sample size in our study (total of n=120). This also ensured that the Pareto task inference relying on these factors had a sufficient sample size.
All omic and clinical data were available for at least n=100 samples, ensuring that correlation tests have a power of 80% to detect non-zero coefficients r>0.277 given a type I error rate of 5% (Hulley et al. Lippincott Williams & Wilkins 2013), and thus ensuring that downstream analyses such as gene set enrichment analysis relying on correlation test p-values are also properly powered.
Combining the MESOMICS, Bueno, and TCGA cohorts allows to reach n=300 samples with exonic sequencing data, allowing to detect recurrent alterations with a prevalence of 1% in malignant pleural mesothelioma in at least 2 samples with a power of 80% (based on the binomial distribution).

Data exclusions

Pre-established exclusion criteria were as follows: samples were excluded if they were derived from metastasis or a participant who had undergone chemotherapy for the treatment of malignant pleural mesothelioma. These samples were excluded as we aimed to identify whether particular molecular patterns or profiles in primary tumours were associated with disease progression and patient prognosis, therefore we examined only the molecular profile of primary untreated mesothelioma samples obtained at diagnosis.

Replication

We replicated our main findings independently on three external datasets: two of similar malignant pleural mesothelioma cohorts of sizes n=181 from Bueno et al. Nat. Genet. 2016 and n=73 from Hmeljak et al. 2019, and one of n=59 cancer cell lines from de Reynies et al. Clin. Cancer Res. 2014 and Iorio et al. Cell 2016.

Randomization

Samples were assigned a histopathological class through central pathological review. We assessed the importance of covariables like age, sex, smoking status, and asbestos exposure in multivariate analyses using regression analyses. We controlled for these variables in statistical analyses by adding them as covariables in the regressions when appropriate.

Blinding

The investigators were not blinded to the histopathological class during the unsupervised analyses, but this was unnecessary because these analyses do not take into account any sample information except the genomic alterations, gene expression, and methylation levels. The investigators could not be blinded to the subsequent differential analyses, which required knowledge of the group membership for each sample.

Reporting for specific materials, systems and methods

We require information from authors about some types of materials, experimental systems and methods used in many studies. Here, indicate whether each material, system or method listed is relevant to your study. If you are not sure if a list item applies to your research, read the appropriate section before selecting a response.

Materials & experimental systems

n/a	Involved in the study	
☐	☒	Antibodies
☒	☐	Eukaryotic cell lines
☒	☐	Palaeontology and archaeology
☒	☐	Animals and other organisms
☐	☒	Human research participants
☒	☐	Clinical data
☒	☐	Dual use research of concern

Methods

n/a	Involved in the study	
☒	☐	ChIP-seq
☒	☐	Flow cytometry
☒	☐	MRI-based neuroimaging

Antibodies

Antibodies used	Santa Cruz BAP1 antibody (cloneC-4) catalog number sc-28383
Validation	BAP1 antibody was purchased from Santa Cruz (catalog number sc-28383). Immunohistochemistry was performed on FFPE tumor sections 3µm tissue sections after pretreatment using a Ventana's Immunostainer (BenchMark Ultra). After pre-treatment (CC1 solution), primary antibody was applied (dilution 1:50) during 30 min. The detection kit ultra-view (Universal DAB Detection kit, Ventana) was used following manufacturer's recommendations. BAP1 (clone C4) (Santa Cruz: dilution one to 50) nuclear staining was considered positive (when nuclear expression was retained) or negative (complete loss of staining of all tumor cells with a positive internal control on the slides [fibroblast, lymphocytes, etc.]).

Human research participants

Policy information about studies involving human research participants

Population characteristics	Age: median of 67.5<br>Sex: 32 females and 88 males<br>n = 120 chemonaive at the moment of sample collection and three no chemonaive removed from the analyses.<br>The samples used in this study belong to the virtual biorepository French MESOBANK, whose guidelines include obtaining the informed consent from all subjects and no participant compensation.
Recruitment	There has not been a prospective recruitment of patients specifically done for this study. The biological specimens were previously collected for clinical routine and stored in the biobanks of the collaborative hospitals, which made the de-identified samples available for research.
Ethics oversight	International Agency for Research on Cancer ethics committee

Note that full information on the approval of the study protocol must also be provided in the manuscript.
